# Discovery of [1,2,4]triazolo[1,5‐*a*]pyrimidine‐Imatinib Hybrids With Selective Cytotoxic Activity: A Mechanistically Divergent Series From Direct BCR‐ABL1 Inhibition

**DOI:** 10.1002/cmdc.202501100

**Published:** 2026-04-19

**Authors:** Stefany Castro Bazan Moura, Andressa Paula de Oliveira, Joao de Mello Rezende Neto, Rafael Ferreira Dantas, Floriano Paes Silva‐Jr, Luiz Claudio Pimentel, Mayara Salles do Nascimento Carvalho, Debora Inacio Leite, Daiane Vitoria da Silva, Monica Macedo Bastos, Nubia Boechat

**Affiliations:** ^1^ Laboratorio de Sintese de Farmacos Instituto de Tecnologia em Farmacos‐Farmanguinhos Rio de Janeiro Brazil; ^2^ Programa de Pos‐graduação em Farmacologia e Quimica Medicinal Instituto de Ciencias Biomedicas Centro de Ciencias da Saude Rio de Janeiro Brazil; ^3^ Laboratorio de Bioquimica Experimental e Computacional de Farmacos Instituto Oswaldo Cruz Rio de Janeiro Brazil

**Keywords:** BCR‐ABL1, chronic myeloid leukemia, drug discovery, enzymes, medicinal chemistry

## Abstract

Chronic myeloid leukemia treatment faces the challenge of resistance to BCR‐ABL1 tyrosine kinase inhibitors. To address this, we rationally designed six new imatinib‐derived compounds **2(a–f)** by replacing the quinoline moiety with a [1,2,4]triazolo[1,5‐*a*]pyrimidine scaffold via classical bioisosterism. The compounds were efficiently synthesized and characterized. Biological evaluation revealed that compound **2a** exhibited cytotoxic activity in BCR‐ABL1‐positive K562 cells (47% viability inhibition at 10 µM, IC_50_ = 9.7 µM). However, enzymatic assays demonstrated that **2a** does not directly inhibit the wild‐type ABL1 kinase, unlike IMT. This finding, coupled with its cytotoxicity in nontumorigenic WSS‐1 cells, indicates an alternative, off‐target mechanism. A clear structure–activity relationship identified the detrimental effect of a ‐CF_3_ substitution. Overall, this work applies bioisosteric replacement to generate a new chemotype and uncovers a mechanistically divergent lead. The distinct, ABL1‐independent mechanism of compound **2a** establishes a solid foundation for future optimization and highlights its potential as a starting point for developing novel antimyeloproliferative agents with a different therapeutic profile.

## Graphical abstract

Discovery of imatinib–triazolopyrimidine hybrids with selective cytotoxicity and a mechanism distinct from classical BCR‐ABL1 inhibition. This bioisosteric strategy introduces a novel compounds, paving the way for innovative antileukemic therapies with differentiated profiles.

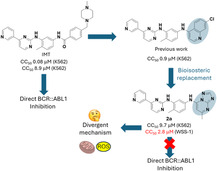

## 1 Introduction

Chronic myeloid leukemia (CML) is characterized by the presence of the Philadelphia chromosome (Ph+), resulting from the reciprocal translocation t(9;22)(q34;q11). This genetic rearrangement generates the oncogenic BCR‐ABL1 fusion gene, which encodes a constitutively active tyrosine kinase protein (TKP). This dysregulated kinase activity drives the pathogenesis of CML and represents a promising molecular target for therapy, as it is absent in healthy cells [1, 2].

Protein kinases (PKs) are enzymes whose dysregulation can lead to severe alterations in cellular processes and is frequently associated with oncogenesis [3]. Structurally, kinase domains contain several conserved regions, including the N‐terminal and C‐terminal lobes, a “hinge” region, a “gatekeeper” residue, and the ATP‐binding site [4].

BCR‐ABL1 tyrosine kinase inhibitors (TKIs) are the first‐line therapy for most CML patients. These drugs target the kinase domain by competitively binding to the adenosine triphosphate (ATP) binding site [5]. Imatinib (IMT), a pioneering BCR‐ABL1 inhibitor, revolutionized CML treatment and became the standard therapy for Ph+ CML [6]. It binds specifically to the inactive conformation (DFG‐out) of the kinase domain, blocking ATP‐dependent substrate phosphorylation, interrupting downstream signaling, and ultimately inducing apoptosis in leukemic cells [7].

**FIGURE 1:**
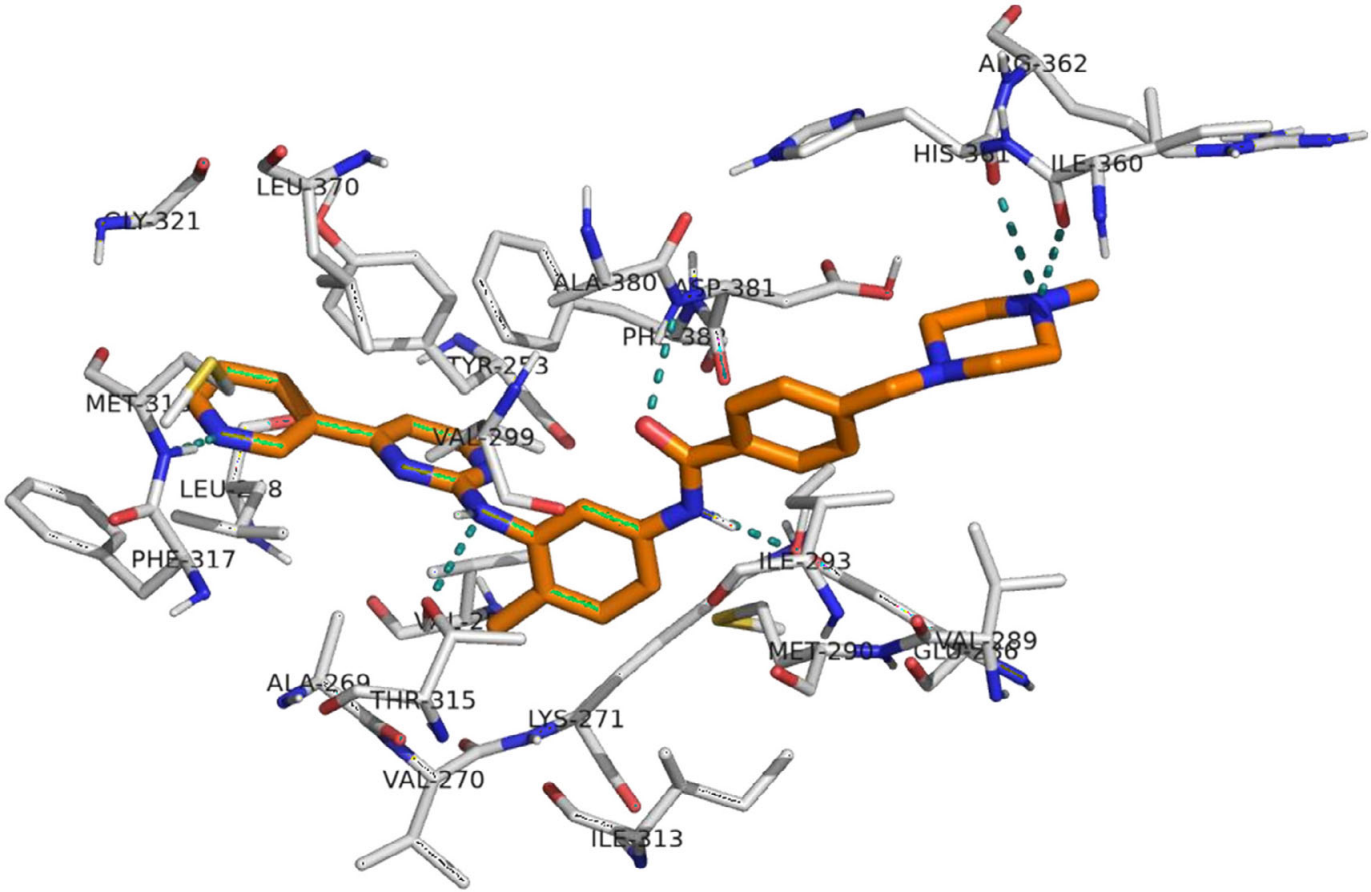
Intermolecular interactions performed by IMT at the active site (Adapted from: http://www.rcsb.org/structure/2HYY).

The structural genesis of IMT began with the phenylaminopyrimidine (PAP) scaffold. Through high‐throughput screening and subsequent medicinal chemistry optimization, the introduction of a pyridine ring led to the phenylaminopyrimidine‐pyridine (PAPP) pharmacophore, which demonstrated significantly increased inhibitory activity against BCR‐ABL1 [8]. Structural analysis of IMT cocrystallized with ABL1 (PDB: 2HYY) reveals that the PAPP moiety forms key hydrogen‐bonding interactions with critical residues in the hinge region, stabilizing the inactive DFG‐out conformation (Figure 1) [9]. Due to this central role, the PAPP scaffold remains a fundamental pharmacophore widely explored in the rational design of new TKIs for CML treatment [10, 11, 12].

Although IMT is preferred as an initial treatment due to its safety profile [13], more than 50% of treated patients develop resistance or intolerance over time [14].

**FIGURE 2:**
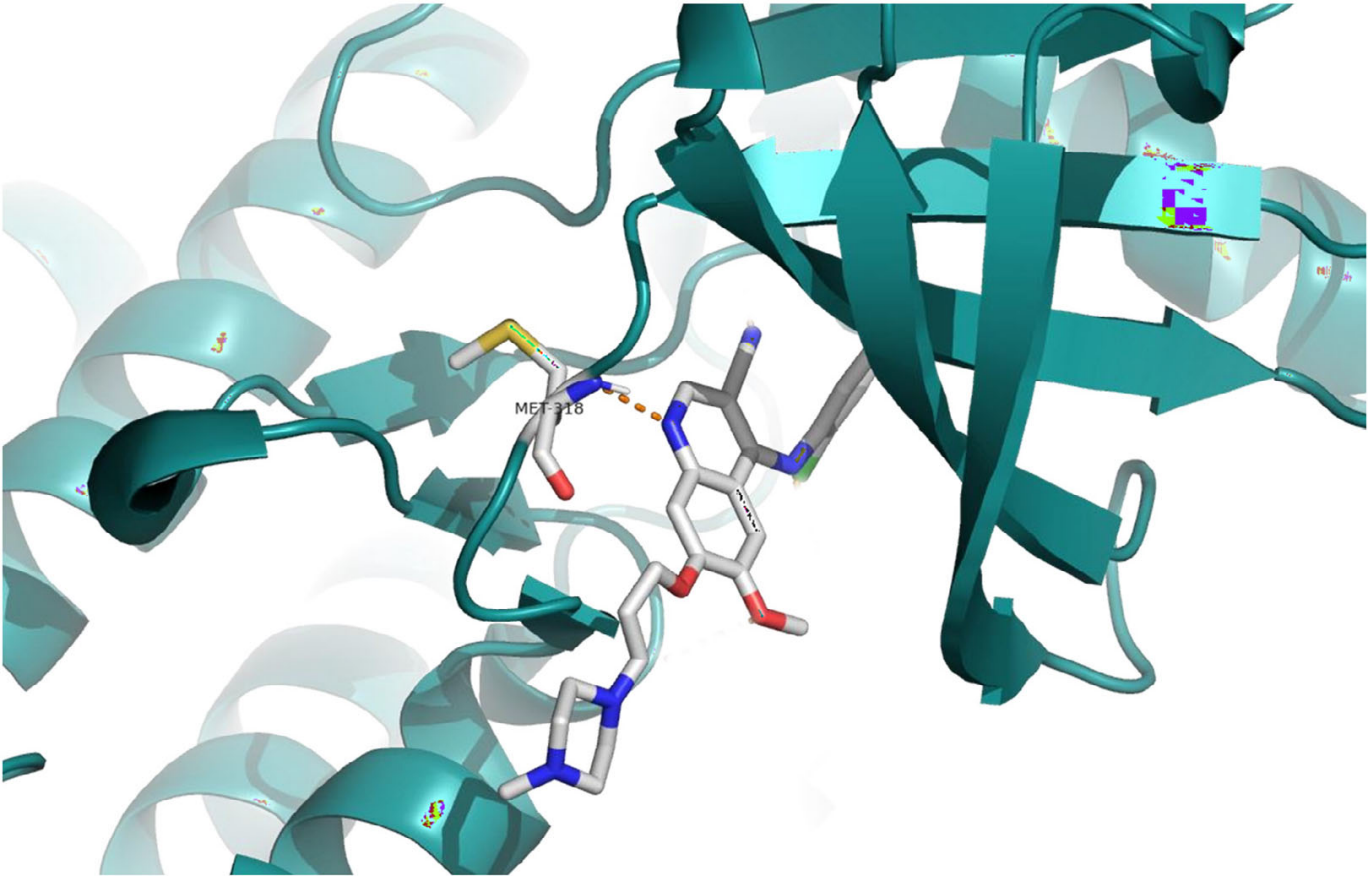
Intermolecular interactions (highlighted with Met318 residue) performed by bosutinib at the active site (Adapted from: http://www.rcsb.org/structure/3UE4).

Due to resistance caused by specific lesions, second‐generation inhibitors have been developed with a rational design to relax binding and maintain specificity, successfully overcoming almost all alterations in ABL1. This second generation includes nilotinib (NLT), dasatinib (DAT), and bosutinib (BST), all designed to circumvent IMT resistance in CML [15, 16]. Among these inhibitors, the BST stands out for possessing the quinoline nucleus, a structure widely found in natural and synthetic products, which has been associated with various biological activities and therapeutic applications [17, 18]. In the case of BST, the quinoline nucleus functions as an essential pharmacophoric fragment, forming a hydrogen bond with the Met318 residue of the hinge region (PDB: 3UE4) (Figure 2) [19], which has been used in the design of new prototypes.

Newer BCR‐ABL1 inhibitors developed for CML treatment have demonstrated faster and deeper molecular responses than IMT, along with efficacy against key resistance mutations. However, treatment outcomes remain suboptimal due to resistance mechanisms and significant adverse effects, particularly arterio‐occlusive events [20, 21]. Therefore, the development of novel BCR‐ABL1 inhibitors that effectively circumvent resistance and present a reduced risk of adverse events is a paramount objective in CML therapeutics.

In pursuit of TKIs with antimyeloproliferative activity, our research group has dedicated efforts to developing compounds that inhibit the central molecular target in CML, the BCR‐ABL1 TKP [10, 11, 12, 22]. A recurring structural feature among our previously synthesized active compounds is the PAPP backbone—a core pharmacophore in IMT and nilotinib (NLT). This fragment interacts effectively within the ATP‐binding site of ABL1, forming essential hydrogen bonds and van der Waals interactions to inhibitory activity (Figure 1) [10].

Among these, derivative **1** emerged as particularly promising. Designed via molecular hybridization, it links the PAPP core to a quinoline ring, a key pharmacophore of bosutinib (BST), combining features of two reference TKIs. Initial screening against K562 cells showed it inhibited viability by 46% at 1 µM and 96% at 10 µM. For comparison, under the same conditions, IMT showed 53% and 58% inhibition, while BST showed 63% and 74% inhibition, respectively. Although less potent at 1 µM, compound **1** demonstrated superior efficacy at 10 µM. Its cytotoxic concentration (CC_50_) in K562 cells was 0.9 µM, compared to 0.08 µM for IMT. However, in nontumor WSS‐1 cells, its CC_50_ was 0.2 µM, markedly lower than that of IMT (8.9 µM), resulting in a reduced selectivity index (SI). This finding motivated further structural optimization [12].

**FIGURE 3:**
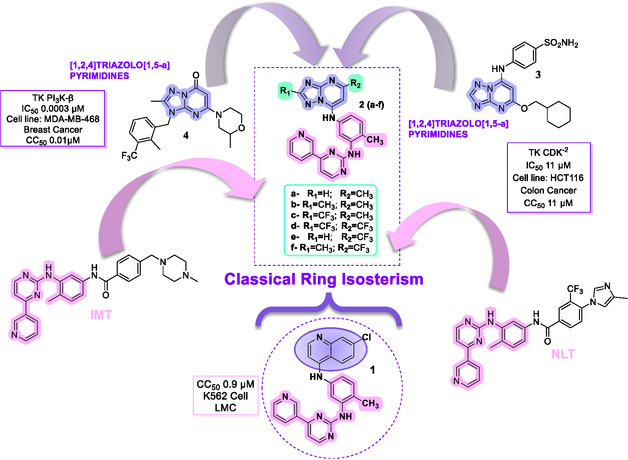
Rational design of novel compounds **2**(**a–f**) using classical ring isosterism as a Medicinal Chemistry strategy.

Building on these results, compound **1** served as the prototype for the rational design of a new series of derivatives, **2a–f**. In these hybrids, the PAPP scaffold was retained, while the quinoline nucleus was strategically replaced by a [1,2,4]triazolo[1,5‐*a*]pyrimidine system. This nucleous was selected not only for its bioisosteric properties but also because it represents a recognized privileged scaffold in medicinal chemistry, known for its versatility in generating bioactive molecules across multiple target classes [23]. The modification applies the principle of classical aromatic ring bioisosterism (Figure 3), aiming to maintain spatial and electronic properties while exploring potential pharmacokinetic and pharmacodynamic advantages. The nitrogen‐rich heterocycle is expected to improve aqueous solubility and modulate the binding profile [24, 25].

It is noteworthy that TKPs possess a highly conserved hinge region. The [1,2,4]triazolo[1,5‐*a*]pyrimidine scaffold has demonstrated significant molecular interactions within this domain in structure–activity relationship (SAR) studies targeting kinases such as CDK2, EGFR, and VEGFR‐2 [26]. However, its application in the design of BCR‐ABL1 inhibitors remains relatively unexplored, highlighting the relevance of the present investigation. We anticipate that while the triazolopyrimidine core may interact with the conserved hinge region of various kinases, pharmacological selectivity for BCR‐ABL1 will be mediated by the PAPP pharmacophore, which is rationally designed for specific interactions with this target [10].

**FIGURE 4:**
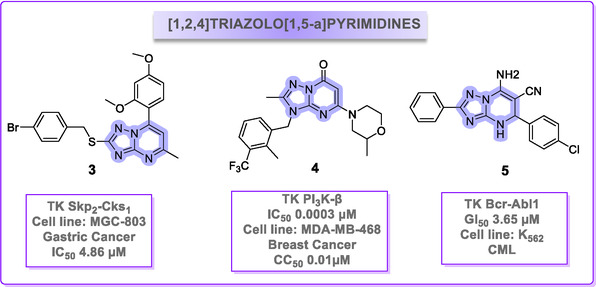
[1, 2, 4]triazolo[1,5‐*a*]pyrimidine derivatives (**3**, **4** and **5**) tested as ITKs.

Recent studies underscore the therapeutic potential of the [1,2,4]triazolo[1,5‐*a*]pyrimidine scaffold. For instance, Hu and colleagues developed a series of derivatives as novel inhibitors of S‐phase kinase‐associated protein 2 (Skp2) [27]. The most promising compound **(3)** exhibited strong binding to Skp2 (IC_50_ = 4.86 µM) and potent antitumor activity in MGC‐803 and PC‐3 cell lines. Molecular docking suggested this affinity stems from a π‐stacking interaction between its C7 phenyl group and the PHE226 residue of Skp2. The compound inhibited proliferation, migration, and induced S‐phase arrest in vitro, and suppressed tumor growth in a xenograft model (Figure 4).

Employing a different rationale, compound **4** was developed to selectively target PTEN‐null tumor cells [28]. It functions as a potent and selective PI3K‐β inhibitor (IC_50_ = 3 nM), effectively suppressing AKT phosphorylation and cell proliferation in PTEN‐deficient MDA‐MB‐468 cells [29].

Of particular relevance to this work, Eldeeb et al. demonstrated that triazolopyrimidine derivatives can act as core hinge‐binders, forming essential hydrogen bonds with key residues (e.g., Met769 of EGFR, Leu83 of CDK2) [26]. Among these, derivative **5** exhibited promising broad‐spectrum anticancer activity, including against the K562 (CML) cell line (GI_50_ = 3.6 µM). Notably, the compound demonstrated low cytotoxicity against normal MCF‐10A cells (IC_50_ = 80.3 µM), resulting in a promising selectivity index (SI) (>22). This result highlights the inherent potential of the triazolopyrimidine scaffold for conferring selective anticancer activity. This underscores the scaffold's potential for achieving selective cytotoxicity.

Based on this evidence, we employed the [1,2,4]triazolo[1,5‐*a*]pyrimidine scaffold to design the new derivatives **2(a–f)**. This strategy aimed to modulate molecular interactions at the catalytic site of BCR‐ABL1, potentially yielding new inhibitors with optimized profiles. Herein, we report the synthesis, full characterization, and comprehensive biological evaluation of this series against K562 and WSS‐1 cell lines, as well as in a wild‐type ABL1 enzymatic assay. While our design was rooted in kinase inhibition, the pharmacological profiling revealed unexpected and insightful results, leading to the identification of a cytotoxic compound with a mechanism distinct from direct BCR‐ABL1 targeting.

## 2 Results and Discussion

### 2.1 Chemistry

**SCHEME 1:**
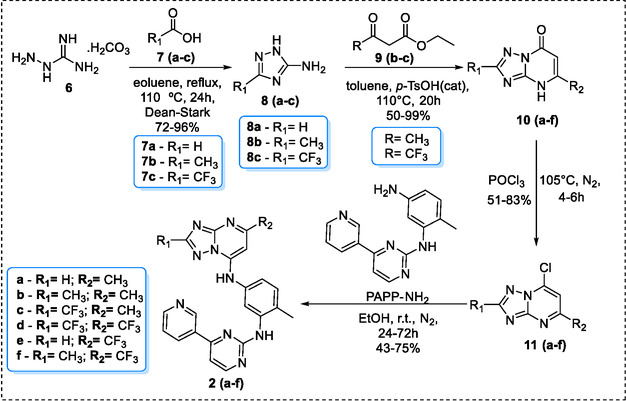
Synthesis of products **2(a–f)**.

The synthesis of target molecules **2(a–f)** was achieved using a four‐step linear route (Scheme 1). The first step involved a reaction between aminoguanidine bicarbonate (**6**) and various carboxylic acids (**7a–c**). The initial condensation, followed by an intramolecular cyclization over 24 h, yielded the 5‐amino‐1*H*−1,2,4‐triazole intermediates (**8a–c**) with yields of 72%–96%.

Subsequently, a *p*‐toluenesulfonic acid‐catalyzed condensation of aminotriazoles **8(a–c)** with either ethyl acetoacetate (**9a**) or ethyl 4,4,4‐trifluoroacetoacetate (**9b**) afforded the target [1,2,4]triazolo[1,5‐*a*]pyrimidin‐7(4*H*)‐one derivatives **10(a–f)**. The products were isolated as white solids in 50%–99% yield after recrystallization from water. Although a keto‐enol tautomeric equilibrium has been previously reported for this heterocyclic system [25, 30], spectroscopic evidence suggested the predominance of the keto form. The FT‐IR spectra of compounds **10(a–f)** exhibited a strong carbonyl stretching vibration (ν) in the 1698–1660 cm^−1^ range, with a notable absence of the O–H stretching band expected for the enol tautomer around 3500 cm^−1^. This observation indicates that the keto form is the major tautomer in the solid state.

The preparation of the key chlorinated derivatives **11(a–f)** consisted of treating the precursors **10(a–f)** with phosphorus oxychloride (POCl_3_) at 105°C for 4–6 hr, resulting in yields of 35%–83%. The absence of axial deformation related to carbonyl and the appearance of absorption in the range between 882–771 cm‐1, corresponding to the axial deformations of C‐Cl, demonstrated that the substitution had occurred.

The target IMT analogs **2(a–f)** were synthesized via a nucleophilic aromatic substitution between the amine PAPP and the key intermediates **11(a–f)**. The reaction was conducted in absolute ethanol at room temperature. Upon completion, the in situ generated HCl was neutralized by adding 20% aqueous NaOH, and subsequent addition of ice‐cold water induced the precipitation of the products, which were isolated in yields of 43%–75%. Formation of the desired C—N bond was confirmed by ^1^H NMR spectroscopy through the appearance of a new, broad singlet between δ 9.04 and 9.11 ppm, assigned to the secondary amine (N–H) proton. This signal was readily distinguished from that of the primary amine protons of the starting material PAPP, observed at δ 10.40 ppm. The structures of all derivatives were unambiguously characterized by FT‐IR, multinuclear NMR (^1^H, ^13^C, ^19^F), and high‐resolution mass spectrometry (HRMS‐TOF). The purity of the final compounds was determined by high‐performance liquid chromatography (HPLC).

The analysis of the ^13^C NMR spectra of compounds **2(a–f)** enabled the identification of methyl and aromatic carbon signals. Methyl carbons exhibited characteristic chemical shifts, while the C‐6 carbon of the heterocyclic rings was observed in the range of 85.7–91.6 ppm. Carbons located between two nitrogen atoms, such as C‐2 and C‐3a, were more deshielded, showing signals between 146.7 and 166.0 ppm. The quaternary carbon C‐7 displayed chemical shifts in the 143.2–148.9 ppm range. Aromatic carbons of the PAPP core were observed between 107.9 and 159.3 ppm, whereas the corresponding quaternary carbons appeared in regions consistent with substituted aromatic structures. Specifically, quaternary carbons were assigned at 129.2–130.8 ppm (C‐9), 133.2–134.5 ppm (C‐13), 138.3–138.6 ppm (C‐14), 160.7–160.8 ppm (C‐16), 161.4–161.8 ppm (C‐20), and 131.9–132.2 ppm (C‐22).

### 2.2 In Vitro Biological Evaluation

Biological evaluations of compounds **2(a–f)** were performed in K562 and WSS‐1 cells. As previously described, K562 is a CML cell line, and WSS‐1 is a healthy human cell line. WSS‐1 cells were used as a reference for the calculation of the selectivity index (SI).

### 2.3 Cytotoxic Effects in K562 and WSS‐1 Cells

**FIGURE 5:**
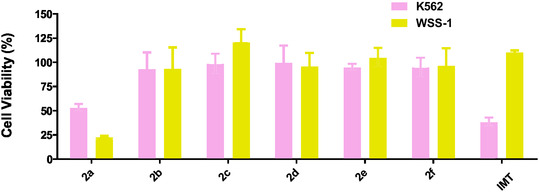
Screening of IMT and its **2(a–f)** derivatives at concentrations of 10 μM against human cell lines K562 (in pink) and nontumoral cells WSS‐1 (in yellow). Bars represent the mean ± standard deviation.

Initial screening of the synthesized compounds **2(a–f)** and the **IMT** standard was performed. The cytotoxic activity results at 10 µM, presented in Figure 5, highlight derivative **2a** as the most promising candidate within the series.

The anti‐leukemic activity against the K562 cell line is presented in Table 1. Compound **2a** exhibited **a** 47% inhibition of cell viability, while the other derivatives showed very low activity (≤7%), indicating no significant effect.

**TABLE 1:** Screening of compound **2(a–f)** and IMT for antiproliferative activity in the K562 cell line.

Compound	Anti‐Leukemic activity (K562 cells) (%)
2a	47
2b	7
2c	1
2d	0
2e	6
2f	6
IMT	62

Although this value is lower than that of the reference standard **IMT** (62% inhibition under the same conditions), compound **2a** demonstrates pharmacological potential within the series, emerging as the only promising candidate for future optimization. This difference suggests that, while the compound does not surpass the clinical standard, it validates the applied bioisosteric strategy and justifies further studies to improve its potency and selectivity.

Subsequently, a concentration–response curve was generated for compound **2a**, with an IC_50_ value of 9.7 μM (**IMT**, IC_50_ = 0.08 μM). These results are summarized in Table 2.

**TABLE 2:** CC_50_ in K‐562 and WSS‐1 cell lines, confidence interval (CI), and selectivity index (SI) of IMT and derivative 2a.

**CC**_**50**_ **(μM)**					Selectivity Index
**Compound**	K‐562	CI	WSS‐1	CI	WSS‐1/K‐562
**IMT**	0.08	0.05 to 0.10	8.9	7.8 to 10.0	111.25
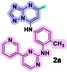	9.7	8.17 to 11.5	2.8	2.5 to 3.2	0.28

*CI – 95% Confidence Interval; SI = CC_50_ (WSS‐1) / CC_50_ (K‐562).

In WSS‐1 cells, compound **2a** showed an IC_50_ of 2.8 µM, while the reference standard **(IMT)** had an IC_50_ of 8.9 µM. This resulted in a selectivity index (SI) of 0.28 for **2a**, compared to 111.2 for IMT. These results indicate that **2a** is more cytotoxic than IMT in this nontumorigenic cell line. Since the BCR‐ABL1 kinase is not present in WSS‐1 cells, this cytotoxicity likely occurs through a nonspecific mechanism. Consequently, although **2a** exhibited the highest inhibition of viability in K562 cells, it was markedly less selective than IMT (Table 1).

**FIGURE 6:**
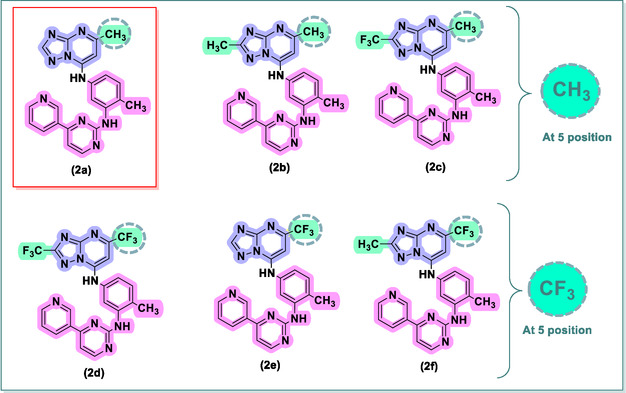
Cytotoxic activity of compounds **2**(**a–f**) in K562 cells.

Regarding the chemical structure of the new derivatives **2(a–f)**, an analysis of the triazolo[1,5‐*a*]pyrimidine core reveals important structure–activity relationships. Compounds **2a**, **2b**, and **2c** share a methyl group at position 5 but differ at position 2 with substituents ‐H (**2a**), ‐CH_3_ (**2b**), and ‐CF_3_ (**2c**), respectively. The highest activity in K562 cells was observed for the unsubstituted derivative, **2a**. The ‐CF_3_ substituted derivative, **2c**, showed no activity, indicating that this group abolishes activity at this position. Furthermore, derivatives **2d**, **2e**, and **2f**, which also possess the ‐CF_3_ group, equally showed no cytotoxic activity against K562 cells. This confirms the deleterious impact of the ‐CF_3_ substitution on activity for this series (Figure 6).

### 2.4 ABL1 Enzymatic Assays

**FIGURE 7:**
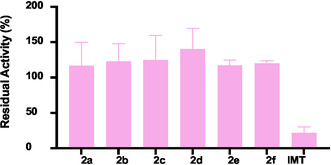
Enzymatic evaluation of compounds **2(a–f)** at a concentration of 100 μM against the wild‐type ABL1 enzyme. Bars represent the mean ± standard deviation.

The new compounds **2(a–f)** were evaluated for their inhibitory activity against the wild‐type ABL1 kinase using the ADP‐Glo assay. The results indicate that none of the compounds exhibited significant inhibitory activity against this target, suggesting that the mechanism responsible for the moderate cellular activity of compound **2a** in K562 assays does not involve direct inhibition of the ABL1 kinase. In contrast, the reference inhibitor **IMT** demonstrated potent inhibition, showing only 22% residual enzymatic activity under the same conditions (Figure 7).

The clear disconnect between the cellular activity of **2a** in K562 cells and its inactivity in the ABL1 kinase assay necessitates a mechanistic hypothesis beyond direct target inhibition. The potent cytotoxicity observed in the nontumorigenic WSS‐1 cell line (IC_50_ = 2.8 µM) further supports a mechanism independent of the BCR‐ABL1 oncogene. This profile suggests that **2a** may act through off‐target effects that induce cellular stress or apoptosis. Potential mechanisms could include the disruption of mitochondrial function, induction of endoplasmic reticulum (ER) stress, or interference with essential metabolic pathways. In this context, Wang and colleagues reported that triazolopyrimidine derivatives can induce mitochondrial apoptosis via reactive oxygen species generation [29]. Alternatively, **2a** might inhibit a different kinase or protein target crucial for K562 cell survival. Notably, off‐target cytotoxicity is a recognized phenomenon for kinase inhibitors, where cellular efficacy can be maintained independently of the intended target's activity [31]. Future chemical proteomics or transcriptomics studies will be essential to deconvolute its precise mode of action. Regardless of the specific target, this divergent mechanism is of significant interest, as it could potentially circumvent common resistance pathways associated with ATP‐competitive BCR‐ABL1 inhibitors.

## 3 Conclusion

In conclusion, we have successfully designed, synthesized, and characterized six new imatinib‐derived compounds **2(a–f)** incorporating the [1, 2, 4]triazolo[1,5‐*a*]pyrimidine scaffold as a bioisosteric replacement for the quinoline ring in this study. The synthetic route proved efficient and reproducible. Biological evaluation identified compound **2a** as the sole derivative with appreciable cytotoxic activity in K562 CML cells. A well‐defined SAR was established, unequivocally demonstrating that the introduction of a ‐CF_3_ group at the triazolopyrimidine core is highly detrimental to activity.

The most significant finding of this study is the mechanistic divergence of the lead compound. Contrary to the initial design hypothesis, comprehensive profiling revealed that compound **2a** showed activity not mediated through direct inhibition of the BCR‐ABL1 kinase, distinguishing it from the parental drug IMT. This discovery transforms the narrative from a structure‐based optimization for a known target to the serendipitous identification of a novel cytotoxic chemotype with an alternative mechanism.

This work establishes a chemical foundation and provides biological insights for future optimization. The promising cytotoxicity of **2a**, despite its current lack of selectivity, warrants further investigation. Future work will therefore focus on elucidating the precise molecular target and mechanism of action of **2a** using phenotypic and ’omics’ approaches. Subsequently, medicinal chemistry campaigns should focus on structural modifications aimed at improving its selectivity window, potentially by exploring different substituents on the triazolopyrimidine core or further modifying the PAPP fragment. Evaluating **2a** against a broader panel of cancer cell lines may also uncover specific therapeutic vulnerabilities. Thus, this study not only validates the application of bioisosteric principles but also opens a new avenue for discovering antimyeloproliferative agents that operate outside the classical BCR‐ABL1 inhibition paradigm.

## 4 Experimental Section

### 4.1 Chemistry

The Sigma–Aldrich reagents purchased were used without prior purification. The solvents purchased from Aldrich, Merck and Tedia were dried and stored under a nitrogen atmosphere using the methodologies described. The reactions were monitored using aluminum sheets coated with silica gel 60 F254 (Merck) TLC plates, and visualization was carried out using a UV lamp (254 and 366 nm).

The melting points (m.p.) were determined using a Fisatom model 430 apparatus and are uncorrected. The FT‐IR absorption spectra was recorded on a Thermo Scientific model Nicolet 6700. Low‐resolution mass spectra were obtained by electrospray ionization (ESI‐MS) in a Bruker AmaZon SL. HRMS analysis was performed by Bruker's Compact (Q‐TOF) device.

The ^1^H and ^13^C NMR analyses were performed in a Bruker HC device at 400 MHz for hydrogen, 100 MHz for carbon, and 376 MHz for fluorine. Tetramethyl silane was used as an internal standard. The chemical shifts (δ) are reported in ppm, and the coupling constants (*J*) are reported in Hertz (Hz). The spin multiplicities are reported as singlet (s), doublet (d), triplet (t), quartet (q), doublet of doublets (dd), doublet of doublet of doublets (ddd), doublet of triplets (dt), and multiplet (m).

HPLC analyses were obtained in a Shimadzu apparatus (VP) with pumps model LC‐20ADXR, degasser DGU‐20A5R, automatic injector SIL‐30AC, oven CTO20AC, controller CBM20A, photodiode array detector (DAFD) SPD‐M20A. The separation was obtained in reversed‐phase columns ACE‐C18 PFP and ACE 3‐C18‐PFP, 150 mm x 3 mm, with a particle diameter of 3 μm. The chromatographic analyses were followed by scanning from 190 nm to 800 nm, and the oven temperature was 30°C.

### 4.2 General Procedure for Synthesis of 5‐Amino‐1*H*‐1,2,4‐Triazole Derivatives (8a–c)

In a two‐necked round‐bottom flask, equipped with a magnetic stir bar, 6.8 g (0.05 mol) of aminoguanidine bicarbonate (**6**) was added along with 0.066 mol of the corresponding acid (**7a‐c**). The reaction mixture was stirred at 60°C for 30 min to allow complete evolution of carbon dioxide. Subsequently, 100 mL of toluene was added, and a Dean‐Stark apparatus fitted with a reflux condenser was attached to the system. The mixture was heated to 110°C under continuous stirring for 24 h. Completion of the reaction was monitored by thin‐layer chromatography (TLC). Upon reaction completion, precipitation was observed. The reaction was cooled in an ice– acetone bath for 1 h. The resulting solid was collected by vacuum filtration and washed with 50 mL of cold toluene.

#### 4.2.1 5‐amino‐1H−1,2,4‐triazole (8a)

Intermediate **8a** was purified by recrystallization from water in the presence of activated charcoal, yielding a white solid and it was obtained in 72% yield from **7a**. m.p. 153–154°C; IR (KBr): 3155, 1683, 1545, 1296 cm^−1^. ESI‐MS ([M+H]^+^, m/z, %): 85.27 (100).

#### 4.2.2 5‐amino‐3‐methyl‐1H−1,2,4‐triazole (8b)

Intermediate **8b** was washed with methanol to afford a pale‐yellow solid, and it was obtained in 96% yield from **7b**. m.p. 146–147°C. IR (KBr): 3422, 3305, 1713, 1419, 1254 cm^−1^. HRMS (ESI) [M+H]^+^, calcd for C_3_H_6_N_4_: 99.0671, found: 99.0661 (Mass error: 4.6 ppm).

#### 4.2.3 5‐amino‐3‐(trifluoromethyl)‐1H−1,2,4‐triazole (8c)

Intermediate **8c** was obtained as a white solid and used in the subsequent step without further purification, and it was obtained in 88% yield from **7c**. m.p. 199°C. IR (KBr): 3488, 3353, 1638, 1488, 1359, 1176, 759 cm^−1^. HRMS (ESI) [M+H]^+^, calcd for C_3_H_3_F_3_N_4_: 153.0389, found: 153.0381 (Mass error: 0.8 ppm).

### 4.3 General procedure for synthesis of [1,2,4]triazolo[1,5‐*a*]pyrimidin‐7(4*H*)‐one derivatives 10a–f

In a 100 mL two‐necked round‐bottom flask equipped with a reflux condenser, 0.01 mol of the corresponding aminotriazole (**8a‐c**) or commercially available 3‐methyl‐1*H*‐ pyrazol‐5‐amine was added along with 13 mL of ethyl acetoacetate or ethyl 4,4,4‐trifluoroacetoacetate (**9b‐c**) (0.11 mol). The reaction mixture was magnetically stirred for 30 min to ensure homogenization. Then, 15 mL of toluene and 0.62 g of *p*‐ toluenesulfonic acid were added. The mixture was refluxed under magnetic stirring for 20 h. Reaction progress was monitored by TLC. After cooling, a precipitate was observed, collected by vacuum filtration, and washed with 50 mL of cold toluene. Intermediates **10a–f** were recrystallized from water in the presence of activated charcoal, yielding white solids in yields ranging from 50% to 99%.

#### 4.3.1 5‐methyl‐[1,2,4]triazolo[1,5‐*a*]pyrimidin‐7(4*H*)‐one (10a)

Intermediate **10a** was obtained as a white solid in 76% yield. m.p. 278–279°C. IR (KBr): 2804, 1662, 1611, 1332 cm^−1^. HRMS (ESI) [M+Na]^+^, calcd for C_6_H_6_N_4_O: 173.0439, found: 173.0439 (Mass error: 2.5 ppm).

#### 4.3.2 2,5‐dimethyl‐[1,2,4]triazolo[1,5‐*a*]pyrimidin‐7(4*H*)‐one (10b)

Intermediate **10b** was obtained as a white solid in 89% yield. m.p. 314–316°C. IR (KBr): 2705, 1692, 1641, 1323 cm^−1^. HRMS (ESI) [M+Na]^+^, calcd for C_7_H_8_N_4_O: 187.0595, found: 187.0586 (Mass error: 2 ppm).

5‐methyl‐2‐(trifluoromethyl)‐[1,2,4]triazolo[1,5‐*a*]pyrimidin‐7(4*H*)‐one (**10c**) Intermediate **10c** was obtained as a white solid in 99% yield. m.p. 265–266°C. IR (KBr): 2873, 1698, 1608, 1401, 753 cm^−1^. HRMS (ESI) [M+Na]^+^, calcd for C_7_H_5_F_3_N_4_O: 241.0312, found: 241.0305 (Mass error: 1.2 ppm).

#### 4.3.3 2,5‐bis(trifluoromethyl)‐[1,2,4]triazolo[1,5‐*a*]pyrimidin‐7(4*H*)‐one (10d)

Intermediate **10d** was obtained as a white solid in 50% yield. m.p. 174–176°C. IR (KBr): 2825, 1698, 1605, 1407, 876, 753 cm^−1^. HRMS (ESI) [M+Na]^+^, calcd for C_7_H_2_F_6_N_4_O: 295.003, found: 295.0023 (Mass error: 0.6 ppm).

#### 4.3.4 5‐(trifluoromethyl)‐[1,2,4]triazolo[1,5‐*a*]pyrimidin‐7(4*H*)‐one (10e)

Intermediate **10e** was obtained as a white solid in 55% yield. m.p. 239–240°C. IR (KBr): 2684, 1698, 1662, 1452, 825, 708 cm^−1^. HRMS (ESI) [M+Na]^+^, calcd for C_6_H_3_F_3_N_4_O: 227.0156, found: 227.0161 (Mass error: −4.4 ppm).

#### 4.3.5 2‐methyl‐5‐(trifluoromethyl)‐[1,2,4]triazolo[1,5‐*a*]pyrimidin‐7(4*H*)‐one (10f)

Intermediate **10f** was obtained as a white solid in 74% yield. m.p. 340–342°C. IR (KBr): 2750, 1698, 1662, 1398, 744 cm^−1^. HRMS (ESI) [M+Na]^+^, calcd for C_7_H_5_F_3_N_4_O): 241.0312, found: 241.0302 (Mass error: 2.2 ppm).

### 4.4 General procedure for synthesis of 7‐chloro‐[1,2,4]triazolo[1,5‐*a*]pyrimidines derivatives (11a‐f)

In a 50 mL two‐necked round‐bottom flask equipped with a reflux condenser 0.006 mol of the corresponding carbonyl intermediate (**11a–f**) and 6 mL (0.0641 mol) of POCl_3_ were added. The reaction mixture was stirred under an inert atmosphere at 105°C for 4–6 h. Reaction completion was monitored by TLC.

After completion, the reaction mixture was poured in crushed ice. The pH was adjusted to neutral with a chilled aqueous solution of 20% NaOH. The resulting precipitate was collected by vacuum filtration and washed with cold distilled water. The aqueous phase was extracted with ethyl acetate or dichloromethane, and the combined organic layers were evaporated. Intermediates **11a–f** were obtained as solids with yields ranging from 35% to 83% and were used in the subsequent step without prior purification.

#### 4.4.1 7‐chloro‐5‐methyl‐[1,2,4]triazolo[1,5‐*a*]pyrimidine (11a)

Intermediate **11a** was obtained as an orange solid in 60% yield = 60%. m.p. 146–148°C. IR (KBr): 3071, 2924, 1608, 1500, 771 cm^−1^. ESI‐MS ([M+H]^+^, m/z, %): 168.79 (99.86).

#### 4.4.2 7‐chloro‐2,5‐dimethyl‐[1,2,4]triazolo[1,5‐*a*]pyrimidine (11b)

Intermediate **11b** was obtained as a white solid in 72% yield. m.p. 150–151°C. IR (KBr): 3065, 2930, 1611, 1524, 861 cm^−1^. ESI‐MS ([M+H]^+^, m/z, %): 182.83 (99.88).

#### 4.4.3 7‐chloro‐5‐methyl‐2‐(trifluoromethyl)‐[1,2,4]triazolo[1,5‐*a*]pyrimidine (**11c**)

Intermediate **11c** was obtained as an orange solid in 83% yield. m.p. 103–105°C. IR (KBr): 3062, 1614, 1524, 876, 750 cm^−1^. ESI‐MS ([M+Na]^+^, m/z, %): 258.84 (99.94).

#### 4.4.4 7‐chloro‐2,5‐bis(trifluoromethyl)‐[1,2,4]triazolo[1,5‐*a*]pyrimidine (11d)

Intermediate **11d** was obtained as a beige solid in 59% yield. m.p. 109–110°C. IR

(KBr): 2924, 1659, 1536, 813, 753, 705 cm^−1^.

#### 4.4.5 7‐chloro‐5‐(trifluoromethyl)‐[1,2,4]triazolo[1,5‐*a*]pyrimidine (11e)

Intermediate **11e** was obtained as a yellow solid in 43% yield. m.p. 233–235°C. IR (KBr): 1638, 1535, 814, 717 cm^−1^.

#### 4.4.6 7‐chloro‐2‐methyl‐5‐(trifluoromethyl)‐[1,2,4]triazolo[1,5‐*a*]pyrimidine (**11f**)

Intermediate **11f** was obtained as an orange solid in 35% yield. m.p. 193–195°C. IR (KBr): 3074, 1608, 1527, 882, 732 cm^−1^. ESI‐MS ([M+Na]^+^, m/z, %): 258.83 (99.94).

### 4.5 General Procedure for Synthesis of *N*1‐([1,2,4]Triazolo[1,5‐*a*]Pyrimidin‐7‐Yl)‐4‐Methyl‐*N*3‐(4‐(Pyridin‐3‐Yl)Pyrimidin‐2‐Yl)Benzene‐1,3‐Diamines (**2a**‐f)

In a 100 mL single‐neck round‐bottom flask, 0.003 mol of the respective chlorinated intermediates **11a–f**, 1 equivalent of the key intermediate (FAPP, kindly provided by Cristália S.A., Rio de Janeiro, Brazil, 2017), and 40 mL (7.2 mol) of anhydrous ethanol were added under an inert atmosphere. The reaction mixture was stirred at room temperature for 24–72 h. Reaction progress was monitored by TLC. Upon completion, the excess solvent was removed under reduced pressure using a rotary evaporator. The pH of the resulting medium was adjusted to 7 with a 20% aqueous NaOH solution, followed by the addition of cold water until precipitation occurred. The solid was collected by vacuum filtration and washed with water.

#### 4.5.1 4‐Methyl‐*N* ^1^‐(5‐methyl‐[1,2,4]triazolo[1,5‐*a*]pyrimidin‐7‐yl)‐*N* ^3^‐(4‐(pyridin‐3‐*yl*)pyrimidin‐2‐yl)benzene‐1,3‐diamine (**2a**)

Compound **2a** was purified by washing with hot water and obtained as an orange solid in 75% yield. m.p. 233–235°C. IR (KBr): 3248, 1608, 1578, 1335 cm^−1^. HRMS (ESI) [M+Na]^+^, calcd. for C_22_H_19_N_9_: 432.1660, found: 432.1650. Mass error: 1.3 ppm. ^1^H NMR (400 MHz, DMSO‐d_6_, δ ppm): 2.31 (s, 3H, CH_3_‐13′), 2.37 (s, 3H, CH_3_‐5′), 6.45 (s, 1H, H‐6), 7.15 (dd, *J* = 8.06, 2.14 Hz, 1H, H‐11), 7.35 (d, *J* = 8.24 Hz, 1H, H‐12), 7.49 (d, *J* = 5.17 Hz, 1H, H‐19), 7.55 (dd, *J* = 7.88, 4.92 Hz, 1H, H‐26), 7.74 (d, *J* = 2.04 Hz, 1H, H‐10), 8.48 (d, *J* = 8.0 Hz, 1H, H‐27), 8.55 (d, *J* = 5.08 Hz, 1H, H‐18), 8.55 (s, 1H, H‐2), 8.72 (dd, *J* = 4.74, 1.22 Hz, 1H, H‐25), 9.09 (s, 1H, NH‐8), 9.28 (d, *J* = 1.72 Hz, 1H, H‐23), 10.39 (s, 1H, NH‐15); ^13^C NMR (75 MHz, DMSO‐d_6_, δ ppm): 17.6 (C‐13′), 24.3 (C‐5′), 89.2 (C‐6), 108.0 (C‐19), 120.3 (C‐11), 120.4 (C‐10), 123.9 (C‐26), 129.6 (C‐9), 131.0 (C‐12), 132.2 (C‐22), 134.1 (C‐13), 134.8 (C‐27), 138.4 (C‐14), 146.1 (C‐7), 147.4 (C‐23), 150.7 (C‐25), 153.9 (C‐2), 154.6 (C‐3a), 159.3 (C‐18), 160.8 (C‐16), 161.4 (C‐20), 163.7 (C‐5); HPLC: 100%.

#### 4.5.2 *N* ^1^‐(2,5‐Dimethyl‐[1,2,4]triazolo[1,5‐*a*]pyrimidin‐7‐yl)‐4‐methyl‐*N* ^3^‐(4‐(pyridin‐3‐yl)pyrimidin‐2‐yl)benzene‐1,3‐diamine (2b)

Compound **2b** was purified by washing with hot water and obtained as a beige solid in 70% yield. m.p. 179–180°C. IR (KBr): 3422; 2972; 1608; 1572; 1392, 1359 cm^−1^. HRMS (ESI) [M+H]^+^, calcd for C_23_H_21_N_9_^+^ 424.1999; found 424.1981. Mass error: 2.8 ppm.

^1^H NMR (400 MHz, DMSO‐d_6_, δ ppm): 2.30, 2.32, 2.47 (3 × s, 3H each, CH_3_‐13′, CH_3_‐5′, CH_3_‐2′), 6.37 (s, 1H, H‐6), 7.13 (dd, *J* = 8.08, 2.16 Hz, 1H, H‐11), 7.32 (d, *J* = 8.24 Hz, 1H, H‐12), 7.46 (d, *J* = 5.16 Hz, 1H, H‐19), 7.48 (dd, *J* = 7.64, 4.52 Hz, 1H, H‐26), 7.72 (d, *J* = 2.08 Hz, 1H, H‐10), 8.41 (dt, *J* = 6.16, 1.19 Hz, 1H, H‐27), 8.54 (d, *J* = 5.16 Hz, 1H, H‐18), 8.69 (d, *J* = 3.36 Hz, 1H, H‐25), 9.04 (s, 1H, NH‐8), 9.26 (s, 1H, H‐23), 10.10 (s, 1H, NH‐15); ^13^C NMR (75 MHz, DMSO‐d_6_, δ ppm): 14.6 (C‐2^′^), 17.6 (C‐13’), 24.6 (C‐C5’), 88.6 (C‐6), 107.9 (C‐19), 120.1 (C‐11), 120.2 (C‐10), 123.6 (C‐26), 129.2 (C‐9), 130.9 (C‐12), 132.0 (C‐22), 134.1 (C‐27), 134.5 (C‐13), 138.3 (C‐14), 145.3 (C‐7), 148.0 (C‐23), 151.3 (C‐25), 155.8 (C‐3a), 159.3 (C‐18), 160.8 (C‐16), 161.6 (C‐20), 163.3 (C‐2), 163.4 (C‐5); HPLC: 100%.

#### 4.5.3 4‐methyl‐*N* ^1^‐(5‐methyl‐2‐(trifluoromethyl)‐ [1,2,4]triazolo[1,5‐*a*]pyrimidin‐7‐yl)‐*N*‐(4‐(pyridin‐3‐yl)pyrimidin‐2‐yl)benzene‐1,3‐diamine (2c)

Compound **2c** was purified by washing with hot methanol and obtained as a yellow solid in 55% yield. m.p. 281°C dec. IR (KBr): 3387, 2984, 1629, 1530, 1371, 729, 702 cm^−1^. HRMS (ESI) [M+Na]^+^ calcd for C_23_H_18_F_3_N_9_: 500.1534, found: 500.1522. Mass error: 1.6 ppm. ^1^H NMR (400 MHz, DMSO‐d_6_, δ ppm): 2.31 (s, 3H, CH_3_‐13′), 2.39 (s, 3H, CH_3_‐5′), 6.54 (s, 1H, H‐6), 7.15 (dd, *J* = 8.0, 2.0 Hz, 1H, H‐11), 7.35 (d, *J* = 8.16 Hz, 1H, H‐12), 7.46 (d, *J* = 5.2 Hz, 1H, H‐19), 7.49 (dd, *J* = 7.88, 4.8 Hz, 1H, H‐26), 7.74 (d, *J* = 1.8 Hz, 1H, H‐10), 8.41 (dt, *J* = 6.16, 1.8 Hz, 1H, H‐27), 8.54 (d, *J* = 5.16 Hz, 1H, H‐18), 8.69 (dd, *J* = 4.76, 1.44 Hz, 1H, H‐25), 9.06 (s, 1H, NH‐8), 9.26 (d, *J* = 1.64 Hz, 1H, H‐23), 10.50 (s, 1H, NH‐15); ^13^C NMR (75 MHz, DMSO‐d_6_, δ ppm): 17.6 (C‐13′), 24.7 (C‐5′), 90.9 (C‐6), 108.0 (C‐19), 119.4 (q, *J* = 268.9 Hz, C‐2′), 120.5 (C‐11), 120.6 (C‐10), 123.7 (C‐26), 129.9 (C‐9), 131.0 (C‐12), 132.0 (C‐22), 133.9 (C‐13), 134.2 (C‐27), 138.4 (C‐14), 146.6 (C‐7), 147.9 (C‐23), 151.3 (C‐25), 154.1 (q, *J* = 38.1 Hz, C‐2), 155.6 (C‐3a), 159.3 (C‐18), 160.8 (C‐16), 161.6 (C‐20), 166.0 (C‐5); ^19^F NMR (376 MHz, DMSO‐d_6_, δ ppm): −64.31 and −64.67 (CF_3_); HPLC: 97.1%.

#### 4.5.4 *N* ^1^‐(2,5‐bis(trifluoromethyl)‐[1,2,4]triazolo[1,5‐*a*]pyrimidin‐7‐yl)‐4‐methyl‐*N* ^3^‐(4‐(pyridin‐3‐yl)pyrimidin‐2‐yl)benzene‐1,3‐diamine (2d)

Compound **2d** was purified by by vacuum filtration using silica gel 60‐flash (0.040– 0.063 mm) in a sintered glass funnel with chloroform/methanol (9.8:0.2) as the eluent. It was obtained as a yellow solid in 59% yield. m.p. 249°C dec. IR (KBr): 3445, 2920, 1603, 1577, 739, 703 cm^−1^. HRMS (ESI) [M+Na]^+^ calcd for C_23_H_15_F_6_N_9_: 554.1252, found: 554.1245. Mass error: 0.4 ppm. ^1^H NMR (400 MHz, DMSO‐d_6_, δ ppm): 2.33 (s, 3H, CH_3_‐13′), 6.83 (s, 1H, H‐6), 7.17 (dd, *J* = 8.02, 1.74 Hz, 1H, H‐11), 7.41 (d, *J* = 8.12 Hz, 1H, H‐12), 7.48 (d, *J* = 5.2 Hz, 1H, H‐19), 7.50 (dd, *J* = 7.98, 4.94 Hz, 1H, H‐26), 7.78 (d, *J* = 1.48 Hz, 1H, H‐10), 8.42 (d, *J* = 8.0 Hz, 1H, H‐27), 8.48 (d, *J* = 5.16 Hz, 1H, H‐18), 8.69 (d, *J* = 3.8 Hz, 1H, H‐25), 9.11 (s, 1H, NH‐8), 9.27 (s, 1H, H‐23), 11.42 (s, 1H, NH‐15); ^13^C NMR (75 MHz, DMSO‐d_6_, δ ppm): 17.7 (CH_3_‐13′), 87.9 (C‐6), 108.1 (C‐19), 119.2 (q, *J* = 269.1 Hz, CF_3_‐C‐2′), 120.5 (q, *J* = 273.7 Hz, CF_3_‐C‐5′), 120.5 (C‐11), 120.6 (C‐10), 123.7 (C‐26), 130.8 (C‐9), 131.3 (C‐12), 131.9 (C‐22), 133.2 (C‐13), 134.2 (C‐27), 138.6 (C‐14), 148.0 (C‐23), 148.9 (C‐7), 151.3 (q, *J* = 34.8 Hz, CF_3_‐C‐5), 151.3 (C‐25), 155.2 (q, *J* = 38.4 Hz, CF_3_‐C‐2), 155.4 (C‐3a), 159.1 (C‐18), 160.8 (C‐16), 161.8 (C‐20); ^19^F NMR (376 MHz, DMSO‐d_6_, δ ppm): –64.52 and –67.92 (s, CF_3_); HPLC: 98.2%.

#### 4.5.5 4‐methyl‐*N* ^3^‐(4‐(pyridin‐3‐yl)pyrimidin‐2‐yl)‐*N* ^1^‐(5‐(trifluoromethyl)‐[1,2,4]triazolo[1,5‐*a*]pyrimidin‐7‐yl)benzene‐1,3‐diamine (2e)

Compound **2e** was purified by washing with hot water and obtained as a pink solid in 51% yield. m.p. 223–226°C. IR (KBr): 3437, 3068, 1605, 1581, 702 cm^−1^. HRMS (ESI) [M+Na]^+^ calculated for C_22_H_16_F_3_N_9_: 486.1378, found: 486.1351. Mass error: 1.7 ppm. ^1^H NMR (400 MHz, DMSO‐d_6_, δ ppm): 2.34 (s, 3H, CH_3_‐ 13′); 6.74 (s, 1H, H‐6); 7.19 (dd, *J* = 8.06, 2.10 Hz, 1H, H‐11); 7.40 (d, *J* = 8.16 Hz, 1H, H‐12); 7.48 (d, *J* = 5.12 Hz, 1H, H‐19); 7.50 (dd, *J* = 8.08, 4.84 Hz, 1H, H‐26); 7.80 (d, *J* = 1.96 Hz, 1H, H‐10); 8.42 (dt, *J* = 6.18, 1.8 Hz, 1H, H‐27); 8.49 (d, *J* = 5.16 Hz, 1H, H‐18); 8.69 (dd, *J* = 4.6, 1.26 Hz, 1H, H‐25); 8.77 (s, 1H, H‐2); 9.10 (s, 1H, NH‐8); 9.27 (d, *J* = 1.6 Hz, 1H, H‐23); 11.08 (s, 1H, NH‐15); ^13^C NMR (75 MHz, DMSO‐d_6_, δ ppm):17.6 (CH_3_‐13′); 85.7 (C‐6); 108.0 (C‐19); 120.6 (C‐11); 120.7 (q, *J* = 273.7 Hz, CF_3_‐5′); 120.8 (C‐10); 123.6 (C‐26); 130.5 (C‐9); 131.2 (C‐12); 131.9 (C‐22); 133.4 (C‐12); 134.2 (C‐27); 138.6 (C‐14); 148.0 (C‐23, C‐7); 150.1 (q, *J* = 34.2 Hz, CF_3_−5); 151.3 (C‐25); 154.9 (C‐3a); 155.8 (C‐2); 159.1 (C‐18); 160.8 (C‐16); 161.7 (C‐20); ^19^F NMR (376 MHz, DMSO‐d_6_, δ ppm): –67.65 (CF_3_); HPLC: 98.1%.

#### 4.5.6 4‐methyl‐*N* ^1^‐(2‐methyl‐5‐(trifluoromethyl) [1,2,4]triazolo[1,5‐a]pyrimidin‐7‐yl)‐*N* ^3^‐(4‐(pyridin‐3‐yl)pyrimidin‐2‐yl)benzene‐1,3‐diamine (2f)

Compound **2f** was purified by column chromatography using dichloromethane/methanol (9.8:0.2) as the mobile phase. It was obtained as a beige solid in 65% yield. m.p. 152–154°C. IR (KBr): 3367, 2919, 1607, 1576, 1365, 799, and 706 cm^−1^. HRMS (ESI) [M+Na]^+^ calculated for C_23_H_18_F_3_N_9_: 500.1534, found: 500.1550. Mass error: –4.1 ppm. ^1^H NMR (400 MHz, DMSO‐d_6_, δ ppm): 2.32 (s, 3H, CH_3_‐13′); 2.56 (s, 3H, CH_3_‐2′); 6.69 (s, 1H, H‐6); 7.16 (dd, *J* = 8.6, 2.06 Hz, 1H, H‐11); 7.38 (d, *J* = 8.2 Hz, 1H, H‐12); 7.47 (d, *J* = 5.28 Hz, 1H, H‐19); 7.49 (dd, *J* = 8.28, 5.16 Hz, 1H, H‐26); 7.77 (d, *J* = 1.92 Hz, 1H, H‐10); 8.41 (dt, *J* = 6.3, 1.74 Hz, 1H, H‐27); 8.48 (d, *J* = 5.16 Hz, 1H, H‐18); 8.69 (d, *J* = 3.56 Hz, 1H, H‐25); 9.08 (s, 1H, NH‐8); 9.26 (d, *J* = 1.36 Hz, 1H, H‐23); 10.99 (s, 1H, NH‐15); ^13^C NMR (75 MHz, DMSO‐d_6_, δ ppm): 14.7 (CH_3_‐2′); 17.6 (CH_3_‐13′); 85.5 (C‐6); 108.0 (C‐19); 120.2 (q, *J* = 273.5 Hz, C‐5′); 120.5 (C‐11); 120.6 (C‐10); 123.7 (C‐26); 130.4 (C‐9); 131.2 (C‐12); 131.9 (C‐22); 133.5 (C‐13); 134.2 (C‐27); 138.5 (C‐14); 147.2 (C‐7); 148.0 (C‐23); 149.6 (q, *J* = 34.2 Hz, C‐5); 151.3 (C‐25); 155.3 (C‐3a); 159.1 (C‐18); 160.8 (C‐16); 161.7 (C‐20); 165.3 (C‐5); ^19^F NMR (376 MHz, DMSO‐d_6_, δ ppm): –67.62 (CF_3_); HPLC: 100%.

### 4.6 Cytotoxicity Assays in K562 and WSS‐1 Cells

The K562 cell line (Rio de Janeiro Cell Bank, BCRJ: 0126, Rio de Janeiro, RJ, Brazil), derived from human CML and characterized by BCR‐ABL1 expression, was cultured in RPMI‐1640 medium (Merck, Darmstadt, Hesse, Germany) containing 10% heat‐inactivated fetal bovine serum (FBS; Vitrocell, Campinas, SP, Brazil). The WSS‐1 human kidney epithelial cell line (ATCC CRL‐2029) was grown in high‐glucose DMEM (Vitrocell), also supplemented with 10% FBS (Vitrocell). All cultures were maintained at 37°C with 5% CO_2_ in a humidified CO_2_ incubator equipped with a water jacket (Forma Series II, Thermo Fisher Scientific, Waltham, MA, USA).

The cytotoxic potential of IMT and its derivatives were evaluated using a fluorometric assay based on the resazurin‐to‐resorufin conversion, which reflects cellular metabolic activity. For this, K562 cells (2 × 10^4^ cells/well) and WSS‐1 cells (5 × 10^4^ cells/well) were seeded into 96‐well plates and treated with 10 µM of compound for 48 h at 37°C. Resazurin (final concentration: 0.01 mg/mL; Merck) was added 1 h before the end of the incubation for K562, and 2 h before for WSS‐1. Fluorescence readings were taken immediately after adding the reagent (t_0_) and again at the end of the incubation (t_*n*_) using a FlexStation 3 microplate reader (Molecular Devices, San Jose, CA, USA) with excitation/emission settings of 560/590 nm. The increase in resorufin fluorescence was determined by subtracting the t_0_ value from t_*n*_. Cell viability was calculated as a percentage relative to the DMSO‐treated control group, which was set as 100%. All experiments were carried out in triplicate.

Additionally, concentration–response curves with 8–10 points were constructed—starting at a maximum concentration of 100 µM—using the same fluorometric assay to determine CC_50_ values, which represent the concentration at which cell viability is reduced by 50% [10].

### 4.7 Kinase Inhibition Assays

The inhibitory effect on ABL1 kinase was assessed using the ABL1 Kinase Enzyme System in combination with the ADP‐Glo Kinase Assay Kit (Promega, Madison, WI, USA), according to the manufacturer's instructions. In summary, each reaction (total volume of 50 μL) contained 2.5 ng of kinase, 1 μg of substrate, 25 μM ATP, and 100 μM of the test compound, all prepared in the provided buffer. Control reactions without enzyme and without compound were included to establish the assay's background luminescence (0% activity) and the reference for full kinase activity (100%), respectively.

The reactions were conducted in 384‐well plates and incubated at room temperature for 1 h. To stop the enzymatic activity and eliminate residual ADP, 5 μL of ADP‐Glo reagent was added, followed by a 40‐minute incubation. Next, 10 μL of the kinase detection reagent was introduced, and the mixture was incubated for an additional 30 min to allow luminescence signal development. All experimental conditions were performed in triplicate.

Luminescence was recorded using a FlexStation 3 multimode plate reader. Kinase activity (KA, %) was determined by first subtracting the mean luminescence of the no‐enzyme control wells from all wells containing kinase. These corrected values were then normalized against the signal from the no‐compound control, which was set as 100% activity. The percentage of inhibition (I, %) was calculated as 100 minus the KA value. IMT at 100 μM was used as a positive control for kinase inhibition.

### 4.8 Statistical Analysis

CC_50_ values were determined by fitting the data to a four‐parameter logistic regression model using GraphPad Prism version 8 for Windows (GraphPad Software, San Diego, CA, USA; www.graphpad.com).

## 5 Author Contributions

**Stefany de Castro Bazan Moura:** investigation (lead), methodology (lead), validation (lead), writing – original draft (lead), writing – review and editing (equal). **Andressa Paula de Oliveira:** visualization (supporting), writing – original draft (supporting), writing – review and editing (supporting). **Joao de Mello Rezende Neto:** formal analysis (supporting), validation (supporting). **Rafael Ferreira Dantas:** data curation (supporting), investigation (supporting), validation (supporting). **Floriano Paes Silva‐Jr:** conceptualization (supporting), formal analysis (supporting), investigation (supporting), methodology (supporting), supervision (supporting). **Luiz Claudio Pimentel:** methodology (supporting), writing – original draft (supporting), writing – review and editing (supporting). **Mayara Salles do Nascimento Carvalho:** writing – original draft (equal), writing – review and editing (equal). **Debora Inacio Leite:** visualization (supporting), writing – original draft (supporting), writing – review and editing (supporting). **Daiane Vitoria da Silva:** writing – review and editing (equal). **Monica Macedo Bastos:** conceptualization (lead), methodology (lead), supervision (lead), visualization (lead), writing – original draft (supporting), writing – review and editing (supporting). **Nubia Boechat:** funding acquisition (lead), project administration (lead), resources (lead), supervision (lead), writing – review and editing (lead).

## Funding

This study was supported by FAPERJ (E‐26.200.861.2021, E‐26/204.033/2024).

## Conflicts of Interest

The authors declare no conflicts of interest.

## 6 Supporting Information

Additional supporting information can be found online in the Supporting Information section. **Supporting Fig. S1**: FT‐IR of compound **2a**. **Supporting Fig. S2**: HRMS of compound **2a**. **Supporting Fig. S3**: ^1^H NMR of compound **2a**. **Supporting Fig. S4**: ^13^C NMR of compound **2a**. **Supporting Fig. S5**: HPLC‐DAD of compound **2a**. **Supporting Fig. S6**: FT‐IR of compound **2b**. **Supporting Fig. S7**: HRMS of compound **2b**. **Supporting Fig. S8**: ^1^H NMR of compound **2b**. **Supporting Fig. S9**: ^13^C NMR of compound **2b**. **Supporting Fig. S10**: HPLC‐DAD of compound **2b**. **Supporting Fig. S11**: FT‐IR of compound **2c**. **Supporting Fig. S12**: ESI‐MS of compound **2c**. **Supporting Fig. S13**: HRMS of compound **2c**. **Supporting Fig. S14**: ^1^H NMR of compound **2c**. **Supporting Fig. S15**: 13C NMR of compound **2c**. **Supporting Fig. S16**: ^19^F NMR of compound **2c**. **Supporting Fig. S17**: HPLC‐DAD of compound **2c**. **Supporting Fig. S18**: FT‐IR of compound **2d**. **Supporting Fig. S19**: ESI‐MS of compound **2d**. **Supporting Fig. S20**: HRMS of compound **2d**. **Supporting Fig. S21**: ^1^H NMR of compound **2d**. **Supporting Fig. S22**: 13C NMR of compound **2d**. **Supporting Fig. S23**: ^19^F NMR of compound **2d**. **Supporting Fig. S24**: HPLC‐DAD of compound **2d**. **Supporting Fig. S25**: FT‐IR of compound **2e**. **Supporting Fig. S26**: ESI‐MS of compound **2e**. **Supporting Fig. S27**: HRMS of compound **2e**. **Supporting Fig. S28**: ^1^H NMR of compound **2e**. **Supporting Fig. S29**: ^13^C NMR of compound **2e**. **Supporting Fig. S30**: ^19^F NMR of compound **2e**. **Supporting Fig. S31**: HPLC‐DAD of compound **2e**. **Supporting Fig. S32**: FT‐IR of compound **2f**. **Supporting Fig. S33**: ESI‐MS of compound **2f**. **Supporting Fig. S34**: HRMS of compound **2f**. **Supporting Fig. S35**: ^1^H NMR of compound **2f**. **Supporting Fig. S36**: ^13^C NMR of compound **2f**. **Supporting Fig. S37**: ^19^F NMR of compound **2f**. **Supporting Fig. S38**: HPLC‐DAD of compound **2f**.

## Supporting information

Supplementary Material

## Data Availability

The data that support the findings of this study are available in the supplementary material of this article.

## References

[1] T. Braun , C. Eide , and B. Druker , “Response and Resistance to BCR‐ABL1‐ Targeted Therapies,” Cancer Cell 37, no. 4 (2020): 530–542, 10.1016/j.ccell.2020.03.006. 32289275 PMC7722523

[2] M. Nascimento , S. Moura , L. Parra , et al., “Ponatinib: A Review of the History of Medicinal Chemistry behind Its Development,” Pharmaceuticals 17 (2024): 1361, 10.3390/ph17101361. 39459001 PMC11510555

[3] J. Cicenas , E. Zalyte , A. Bairoch , and P. Gaudet , “Kinases and Cancer,” Cancers 10, no. 3 (2018): 63, 10.3390/cancers10030063. 29494549 PMC5876638

[4] F. Pauli , E. Barreiro , and M. Barbosa , “Características Estruturais Das Proteínas Cinases e Seus Inibidores Em Uso Clínico,” Revista Virtual De Química 10, no. 5 (2018): 1280–1303, 10.21577/1984-6835.20180088.

[5] R. Roskoski , “Properties of FDA‐Approved Small Molecule Protein Kinase Inhibitors: A. 2023 Update,” Pharmacological Research 187 (2023): 106552, 10.1016/j.phrs.2022.106552. 36403719

[6] D. Fabbro , S. Jacob , and H. Moebitz , “Ten Things You Should Know about Protein Kinases: IUPHAR Review 14,” British Journal of Pharmacology 172, no. 11 (2015): 2675–2700, 10.1111/bph.13096. 25630872 PMC4439867

[7] M. Atwood , D. Fabbro , A. Sokolov , S. Knapp , and H. Schioth , “Trends in Kinase Drug Discovery: Targets, Indications and Inhibitor Design,” Nature Reviews Drug Discovery 20, no. 11 (2021): 839–861, 10.1038/s41573-021-00252-y. 34354255

[8] E. Barreiro and C. Fraga , Chapter 3″ Química Medicinal: As Bases Moleculares DA ação Dos fármacos. Third Ed., (ArtMed Ltd., 2015).

[9] S. Cowan‐Jacob , G. Fendrich , A. Floersheimer , et al., “Structural Biology Contributions to the Discovery of Drugs to Treat Chronic Myelogenous Leukaemia,” Acta Crystallographica Section D: Biological Crystallography 63, no. 1 (2007): 80–93, 10.1107/S0907444906047287. 17164530 PMC2483489

[10] A. Oliveira , S. Moura , L. Pimentel , J. Neto , R. Dantas F. Silva , et al., “New Imatinib Derivatives with Antiproliferative Activity against A549 and K‐562 Cancer Cells,” Molecules 27, no. 3 (2022): 750, 10.3390/molecules27030750. 35164014 PMC8838532

[11] L. Pimentel , A. Cunha , L. Hoelz , et al., “Phenylamino‐Pyrimidine (PAP) Privileged Structure: Synthesis and Medicinal Applications,” Current Topics In Medicinal Chemistry 20 (2020): 227–243, 10.2174/1568026620666200124094949. 31976834

[12] C. Santos , L. Pimentel , H. Canzian , et al., “Hybrids of Imatinib with Quinoline: Synthesis, Antimyeloproliferative Activity Evaluation, and Molecular Docking,” Pharmaceuticals 15, no. 3 (2022): 309, 10.3390/ph15030309. 35337107 PMC8950477

[13] W. Padula , R. Larson , S. Dusetzina , J. Apperley , R. Hehlmann , and etal M.Baccarani , “Cost‐Effectiveness of Tyrosine Kinase Inhibitor Treatment Strategies for Chronic Myeloid Leukemia in Chronic Phase after Generic Entry of Imatinib in the United States,” Journal Of the National. Cancer Institute 108, no. 7 (2016): djw003, 10.1093/jnci/djw003. 26944912 PMC4948567

[14] J. Cortes , and F. Lang , “Third‐Line Therapy for Chronic Myeloid Leukemia: Current Status and Future Directions,” Journal of Hematology & Oncology 14, no. 1 (2021): 1–18, 10.1186/s13045-021-01055-9. 33736651 PMC7976694

[15] E. Weisberg , P. Manley , W. Breitenstei , et al., “Characterization of AMN107, a Selective Inhibitor of Native and Mutant Bcr‐Abl,” Cancer Cell 7, no. 2 (2005): 129–141, 10.1016/j.ccr.2005.01.007. 15710326

[16] F. Rossari , F. Minutolo , and E. Orciuolo , “Past, Present, and Future of Bcr‐Abl Inhibitors: From Chemical Development to Clinical Efficacy,” Journal of Hematology and Oncology 11, no. 1 (2018): 14, 10.1186/s13045-018-0624-2. 29925402 PMC6011351

[17] B. Matada , R. Pattanashettar , and N. Yernale , “A Comprehensive Review on the Biological Interest of Quinoline and Its Derivatives,” Bioorganic & Medicinal Chemistry 32 (2021): 115973, 10.1016/j.bmc.2020.115973. 33444846

[18] P. Yadav , and K. Shah , “Quinolines, a Perpetual, Multipurpose Scaffold in Medicinal Chemistry,” Bioorganic Chemistry 109 (2021): 104639, 10.1016/j.bioorg.2021.104639. 33618829

[19] N. Levinson and S. Boxer , “Structural and Spectroscopic Analysis of the Kinase Inhibitor Bosutinib and an Isomer of Bosutinib Binding to the Abl Tyrosine Kinase Domain,” PLoS ONE 7, no. 4 (2012): e29828, 10.1371/journal.pone.0029828. 22493660 PMC3320885

[20] Y. Li , P. Zeng , J. Xiao , P. Huang , and P. Liu , “Modulation of Energy Metabolism to Overcome Drug Resistance in Chronic Myeloid Leukemia Cells through Induction of Autophagy,” Cell Death Discovery 8, no. 1 (2022): 1–10, 10.1038/s41420-022-00991-w. 35443725 PMC9021256

[21] M. Shoukier , M. Kubiak , and J. Cortes , “Review of New‐Generation Tyrosine Kinase Inhibitors for Chronic Myeloid Leukemia,” Current Oncology Reports 23, no. 8 (2021): 1–8, 10.1007/s11912-021-01087-x. 34125316

[22] L. Azeredo , J. Coutinho , V. Jabor , et al., “Evaluation of 7‐Arylaminopyrazolo[1,5‐a]pyrimidines as Anti‐Plasmodium Falciparum, Antimalarial, and Pf‐Dihydroorotate Dehydrogenase Inhibitors,” European Journal of Medicinal Chemistry 126 (2017): 72–83, 10.1016/j.ejmech.2016.09.073. 27744189

[23] T. Felicetti , M. Pismataro , V. Cecchetti , O. Tabarrini , and S. Massari , “Triazolopyrimidine Nuclei: Privileged Scaffolds for Developing Antiviral Agents with a Proper Pharmacokinetic Profile,” Current Medicinal Chemistry 29, no. 8 (2022): 1379–1407, 10.2174/0929867328666210526120534. 34042030

[24] K. Oukoloff , B. Lucero , K. Francisco , K. Bruden , and C. Ballatore , “2,4‐Tri*a*zolo[1,5‐a]pyrimidines in Drug Design,” European Journal of Medicinal Chemistry 165 (2019): 332–346, 10.1016/j.ejmech.2019.01.027. 30703745 PMC6394845

[25] F. Silveira , “Comparative study of the anti‐*Plasmodium falciparum* activity of triazolopyrimidine, pyrazolopyrimidine, and quinoline derivatives: Identification of new inhibitors of the PfDHODH enzyme” (Doctoral Thesis (Ph.D. in Organic Chemistry – Universidade Federal do Rio de Janeiro‐UFRJ), 2020), 209. 10.1016/j.ejmech.2020.112941 33158577

[26] A. Eldeeb , M. Shahin , M. Jaballah , et al., “Design, Synthesis and Biological Evaluation of Triazolo[1,5‐a]pyrimidine Derivatives as New Antiproliferative Agents with Multikinase Inhibitory Activity,” Bioorganic Chemistry 165 (2025): 108899, 10.1016/j.bioorg.2025.108899. 40913964

[27] K. Hu , Y. Luo , P. Miao , et al., “Discovery of Novel [1,2,4]Triazolo[1,5‐a]pyrimidine Derivatives as Novel Potent S‐Phase Kinase‐Associated Protein 2 (SKP2) Inhibitors for the Treatment of Cancer,” Journal of Medicinal Chemistry 67, no. 18 (2024): 16435–16454, 10.1021/acs.jmedchem.4c01283. 39285177

[28] S. Wang , X. Ma , X. Yuan , B. Yu , Y. Xu , and H. Liu , “Discovery of New [1,2,4] Triazolo[1,5‐a]pyrimidine Derivatives that Kill Gastric Cancer Cells via the Mitochondria Pathway,” European Journal Medicinal Chemistry 203 (2020): 112630, 10.1016/j.ejmech.2020. 32683165

[29] R. M. Sanchez , K. Erhard , M. A. Hardwicke , et al., “Synthesis and Structure–activity Relationships of 1,2,4‐Triazolo[1,5‐a]pyrimidin 7(3H)‐Ones as Novel Series of Potent b Isoform Selective Phosphatidylinositol 3‐Kinase Inhibitors,” Bioorganic & Medicinal Chemistry Letters 22, no. 9 (2012): 3198–3202, 10.1016/j.bmcl.2012.03.039. 22475557

[30] M. O. Gandi , “Síntese de novos derivados triazolopirimidínicos hidrazonas como possíveis agentes ANTI‐plasmodium e inibidores da enzima di‐hidroorotato desidrogenase” (Dissertação (Mestrado em Ciências Biológicas, Farmacologia e Química Medicinal– Universidade Federal do Rio de Janeiro‐UFRJ), 2020), 169f.

[31] A. Lin , C. Giuliano , A. Palladino , et al., “Off‐Target Toxicity Is a Common Mechanism of Action of Cancer Drugs Undergoing Clinical Trials,” Science Translational Medicine 11, no. 509 (2019): eaaw8412, 10.1126/scitranslmed.aaw8412. 31511426 PMC7717492

