# Supplementary material for: Discovery of [1,2,4]triazolo[1,5‐a]pyrimidine‐Imatinib Hybrids With Selective Cytotoxic Activity: A Mechanistically Divergent Series From Direct BCR‐ABL1 Inhibition
Source: ChemMedChem. 2026 Apr 19;21(8):e202501100. doi: 10.1002/cmdc.202501100 (PMC13092336; doi:10.1002/cmdc.202501100)
Supplement: Supplementary file 1 — Supplementary Material [file CMDC-21-e202501100-s001.pdf]

Discovery of [1,2,4]triazolo[1,5-*a*]pyrimidine-Imatinib Hybrids with Selective Cytotoxic Activity: A Mechanistically Divergent Series from Direct BCR-ABL1 Inhibition

Stefany Castro Bazan Moura[1,2], Andressa Paula de Oliveira[1], Joao de Mello Rezende Neto[3], Rafael Ferreira Dantas[3], Floriano Paes Silva-Jr[2,3] Luiz Claudio Pimentel[1], Mayara Salles do NascimentoCarvalho[1], Debora Inacio Leite[1], Daiane Vitoria da Silva[1], Monica Macedo Bastos[1,2] and Nubia Boechat[1,2]

[1]Laboratorio de Sintese de Farmacos, Instituto de Tecnologia em Farmacos-Farmanguinhos, Rua Sizenando Nabuco 100, Manguinhos, Rio de Janeiro 21041-250, RJ, Brazil.

[2]Programa de Pos-graduação em Farmacologia e Quimica Medicinal, Instituto de Ciencias Biomedicas, Centro de Ciencias da Saude, Bloco J, Ilha do Fundão, Rio de Janeiro 21941-902, RJ, Brazil.

[3]Laboratorio de Bioquimica Experimental e Computacional de Farmacos, Instituto Oswaldo Cruz, FIOCRUZ, Av. Brasil 4365, Manguinhos, Rio de Janeiro 21040-360, RJ, Brazil.

* Correspondence: nubia.boechat@fiocruz.br; nboechat@gmail.com; Tel.: +55-21-3977-2465

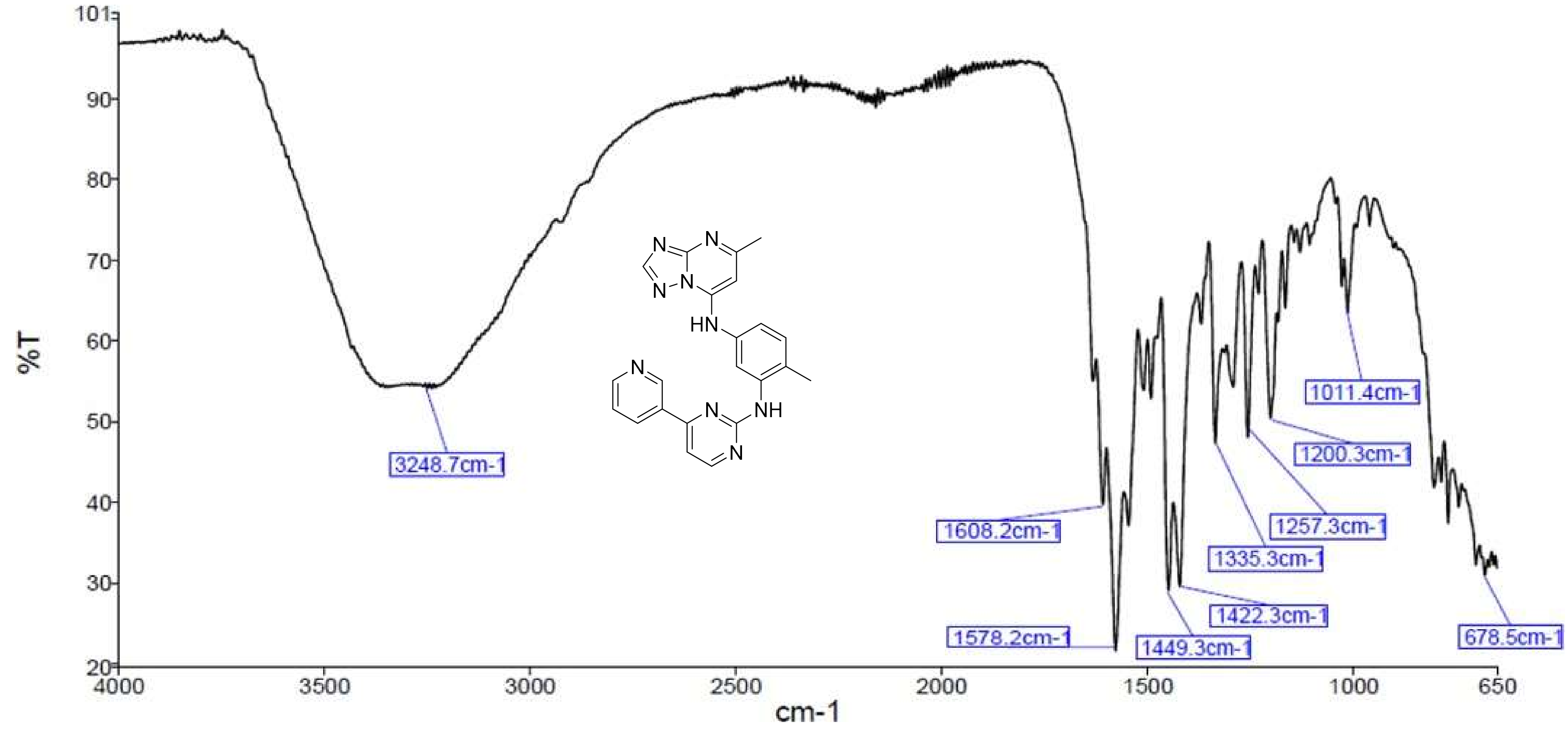

Fig-S.1: FT-IR of compound **2a**.

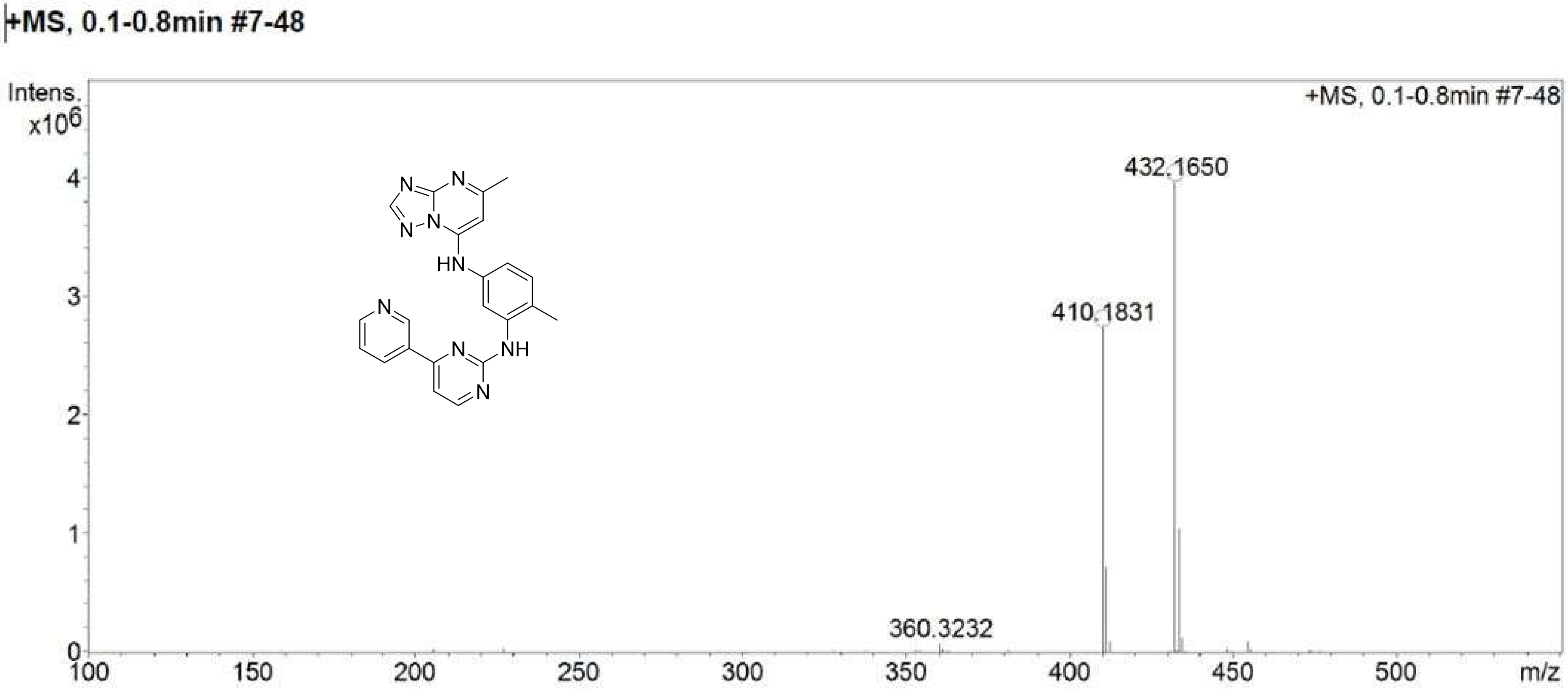

Meas. m/z	#	Ion Formula	m/z	err [ppm]	Mean err [ppm]	rdb	N-Rule	e⁻ Conf	mSigma	Std I	Std Mean m/z	Std I VarNorm	Std m/z Diff	Std Comb Dev
410.183141	1	$C_{22}H_{20}N_9$	410.183618	1.2	0.9	17.5	ok	even	5.5	8.9	n.a.	n.a.	n.a.	n.a.
	2	$C_{21}H_{24}N_5O_4$	410.182281	-2.1	-2.0	12.5	ok	even	8.9	12.0	n.a.	n.a.	n.a.	n.a.
432.165017	1	$C_{22}H_{19}N_9Na$	432.165562	1.3	1.2	17.5	ok	even	6.5	11.3	n.a.	n.a.	n.a.	n.a.
	2	$C_{21}H_{23}N_5NaO_4$	432.164225	-1.8	-1.6	12.5	ok	even	9.3	12.3	n.a.	n.a.	n.a.	n.a.

Fig-S.2: HRMS of compound **2a**.

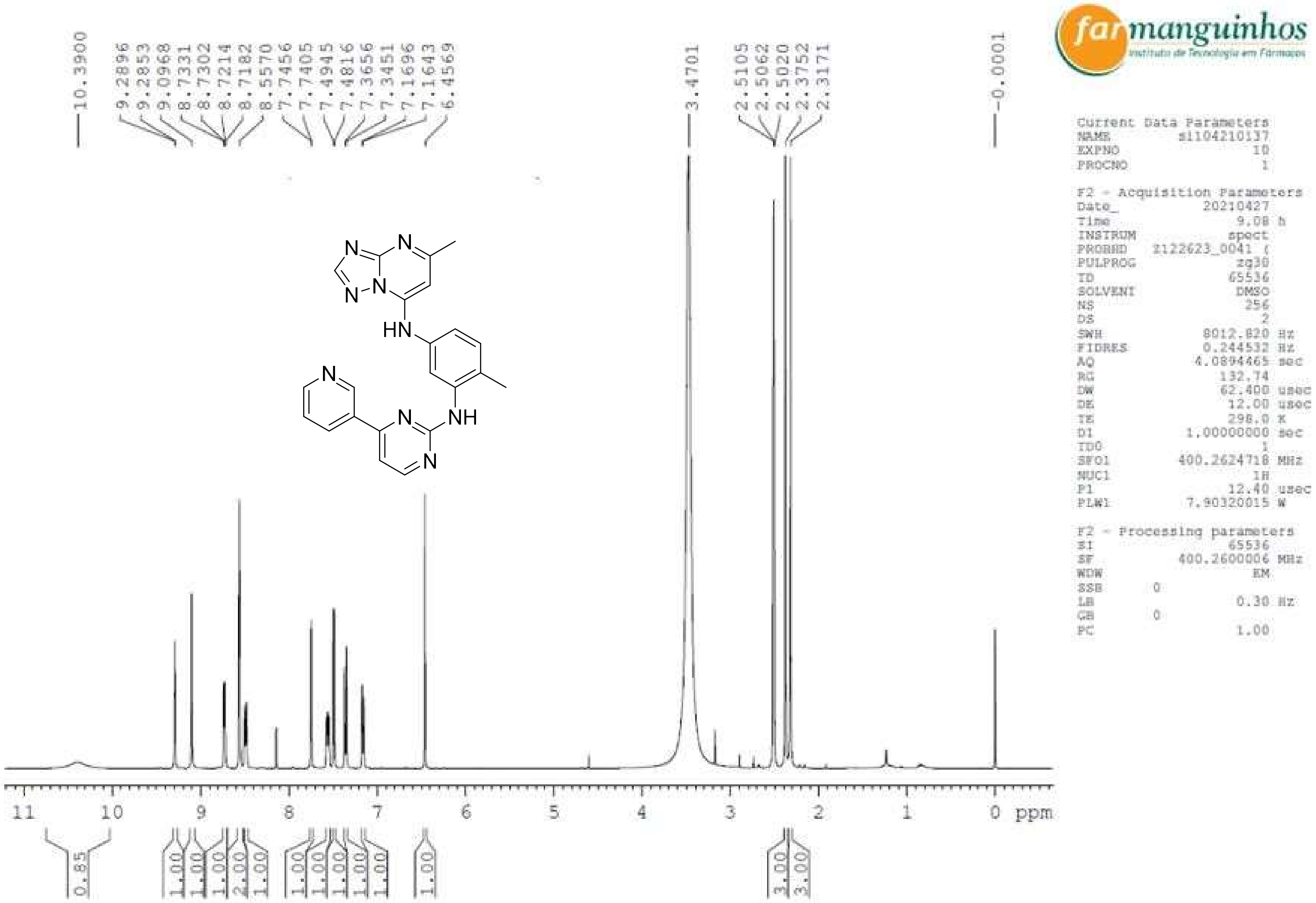

Fig-S.3: ^{1}H NMR of compound **2a**.

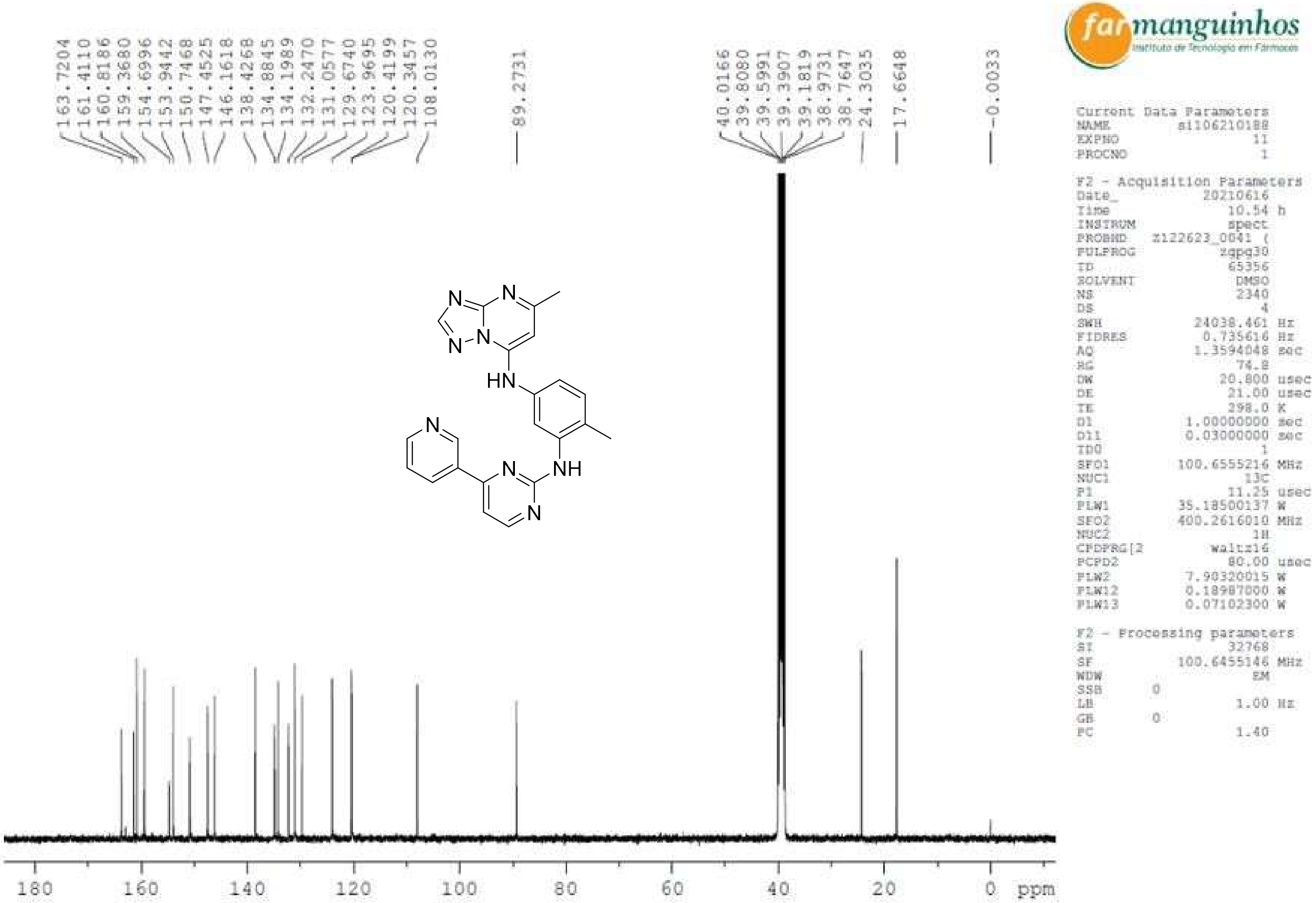

Fig-S.4: ^{13}C NMR of compound **2a**.

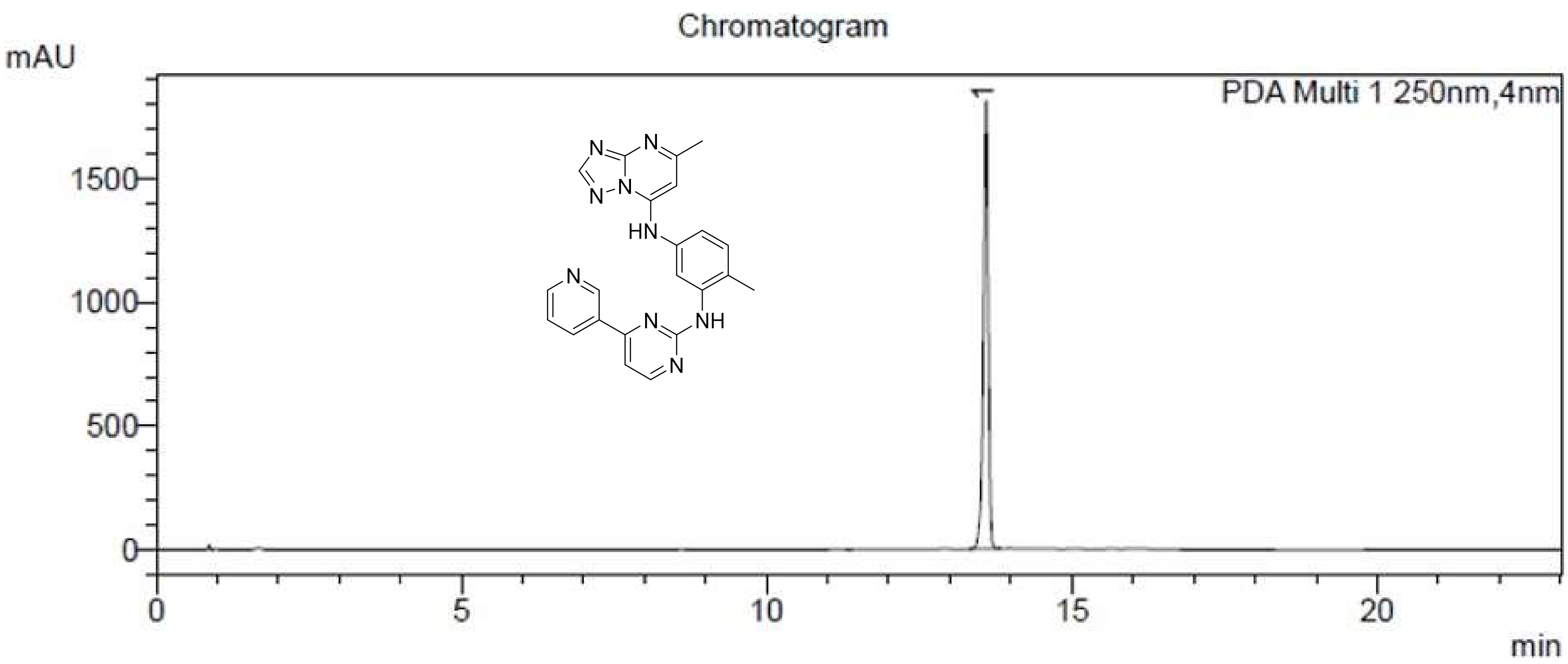

Peak Table

PDA Ch1 250nm

Peak#	Ret. Time	Name	Area	Area%	Theoretical Plates/meter(USP)	Tailing Factor	Resolution(USP)	Capacity Factor(k')
1	13,60		9893690	100,0	787402	0,860	--	--
Total			9893690	100,0				

Fig-S.5: HPLC-DAD of compound **2a**.

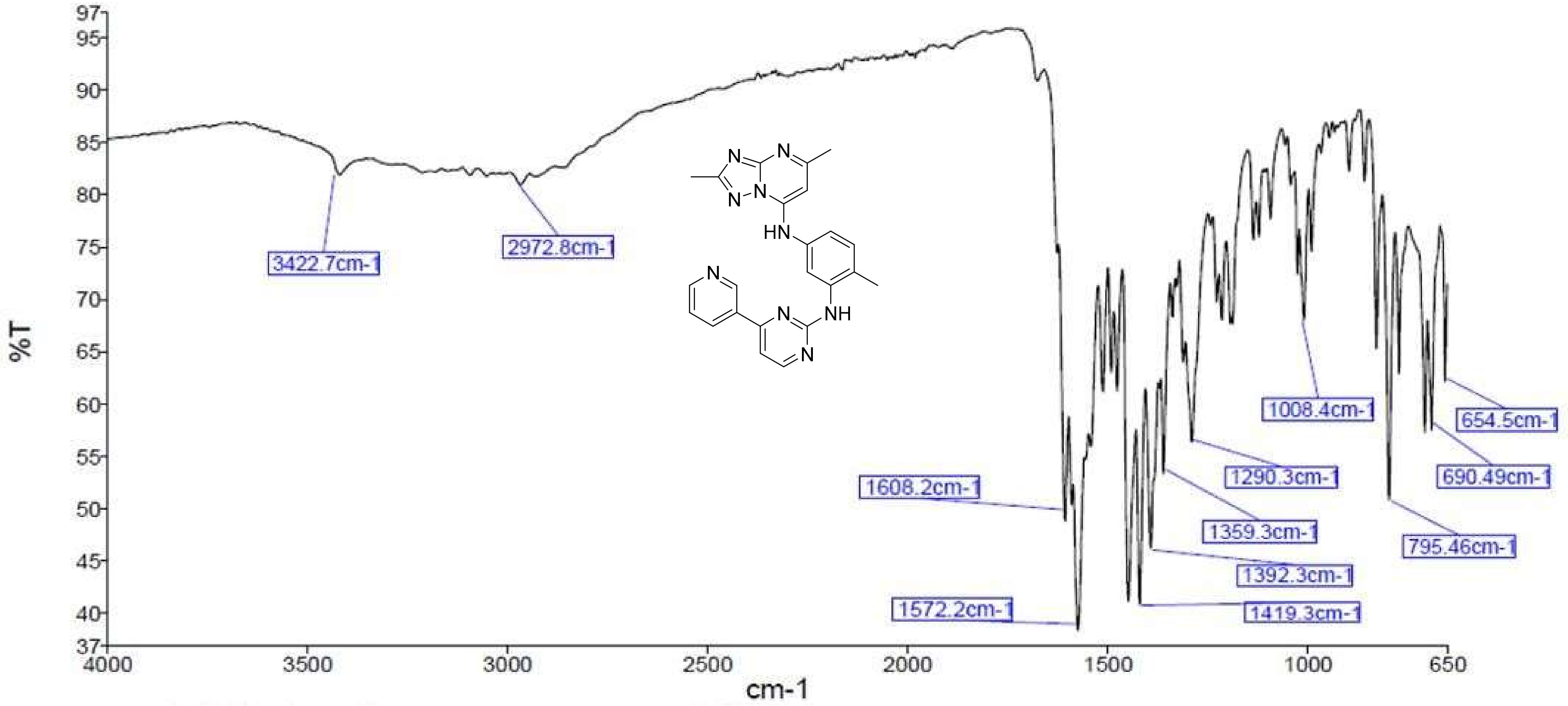

Fig-S.6: FT-IR of compound **2b**.

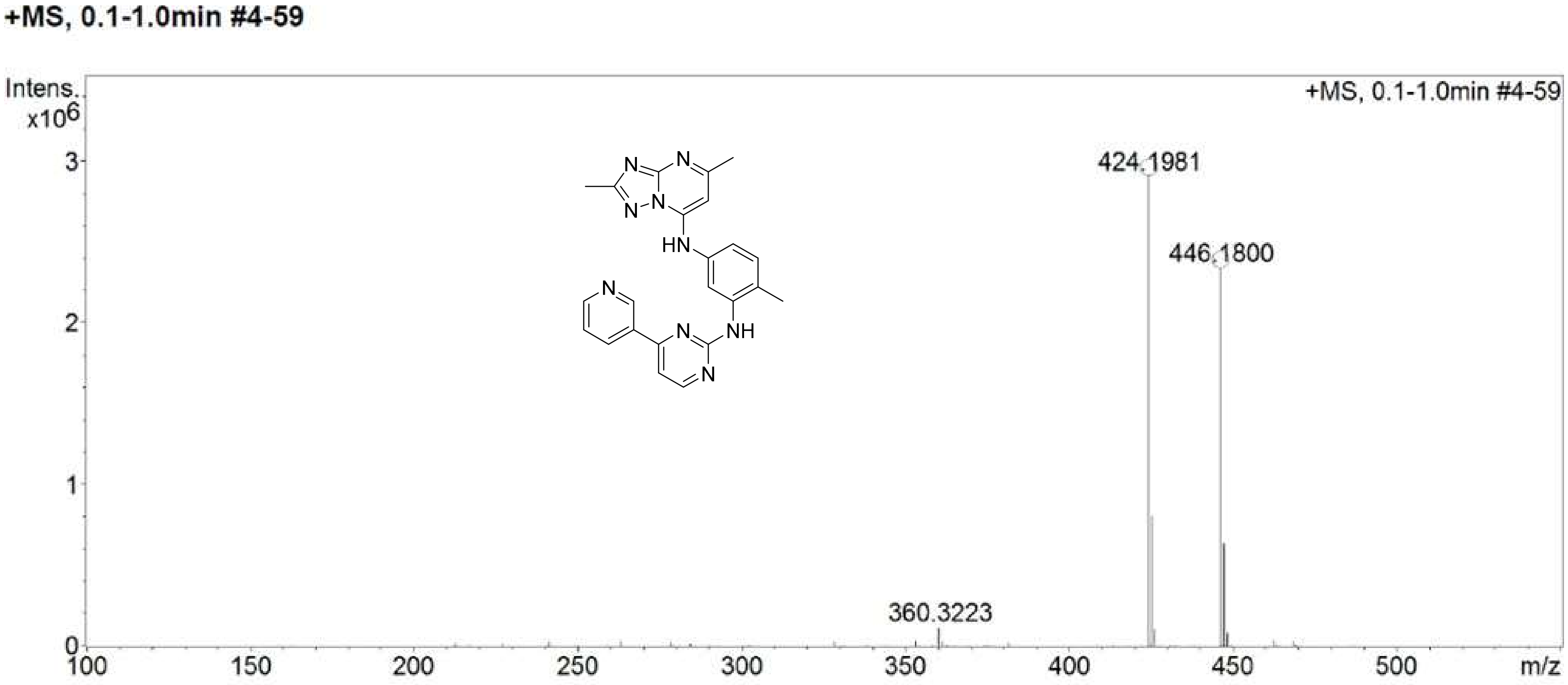

Meas. m/z	#	Ion Formula	m/z	err [ppm]	Mean err [ppm]	rdb	N-Rule	e⁻ Conf	mSigma	Std I	Std Mean m/z	Std I VarNorm	Std m/z Diff	Std Comb Dev
424.198061	1	C23H22N9	424.199268	2.8	2.5	17.5	ok	even	4.3	7.0	n.a.	n.a.	n.a.	n.a.
	2	C22H26N5O4	424.197931	-0.3	-0.3	12.5	ok	even	10.2	13.7	n.a.	n.a.	n.a.	n.a.
	3	C21H30NO8	424.196593	-3.5	-3.2	7.5	ok	even	23.7	33.2	n.a.	n.a.	n.a.	n.a.
446.179966	1	C23H21N9Na	446.181212	2.8	2.7	17.5	ok	even	5.7	9.3	n.a.	n.a.	n.a.	n.a.
	2	C22H25N5NaO4	446.179875	-0.2	-0.0	12.5	ok	even	8.9	11.8	n.a.	n.a.	n.a.	n.a.
	3	C21H29NNaO8	446.178538	-3.2	-2.7	7.5	ok	even	22.3	31.1	n.a.	n.a.	n.a.	n.a.

Fig-S.7: HRMS of compound **2b**.

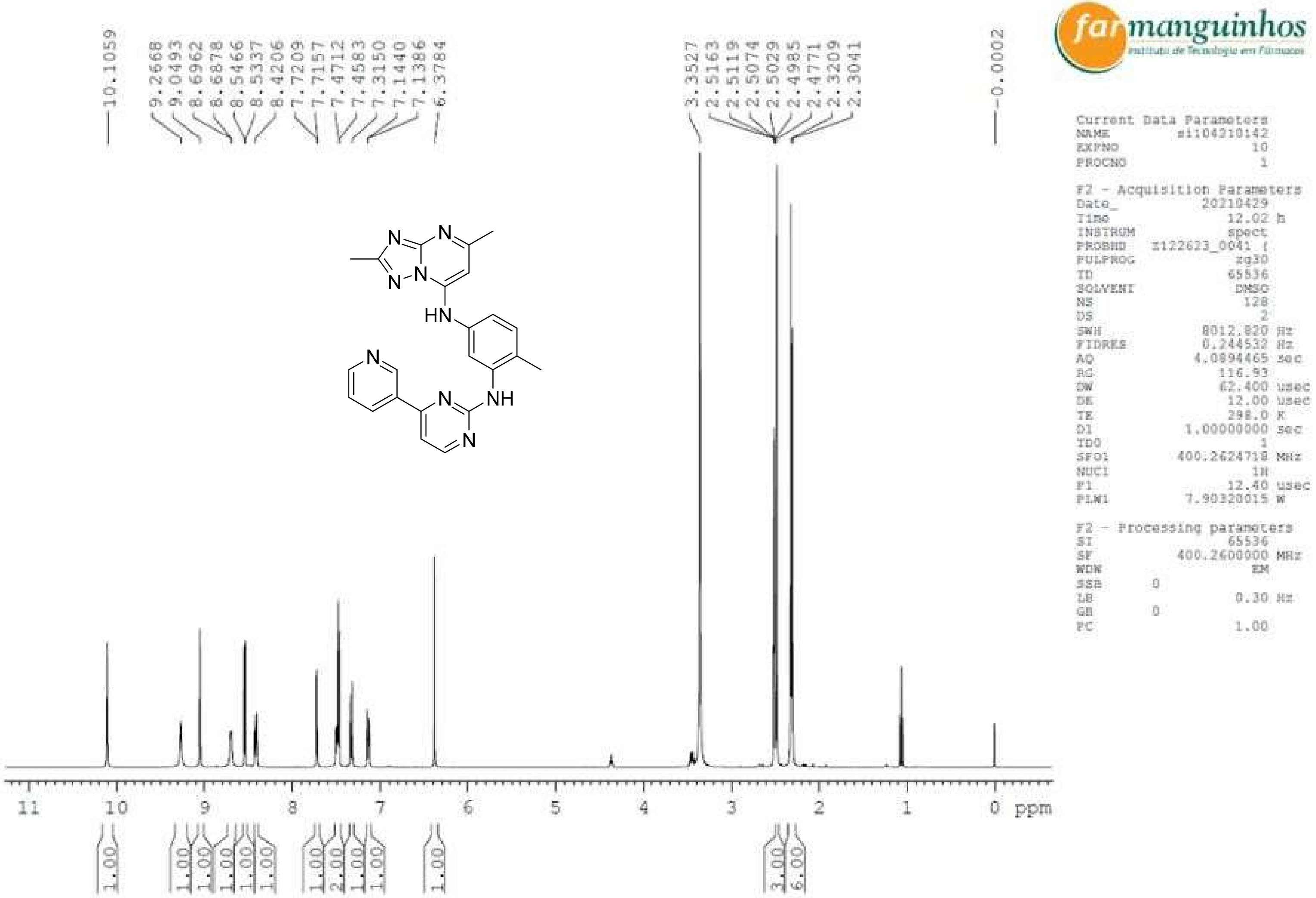

Fig-S.8: ^{1}H NMR of compound **2b**.

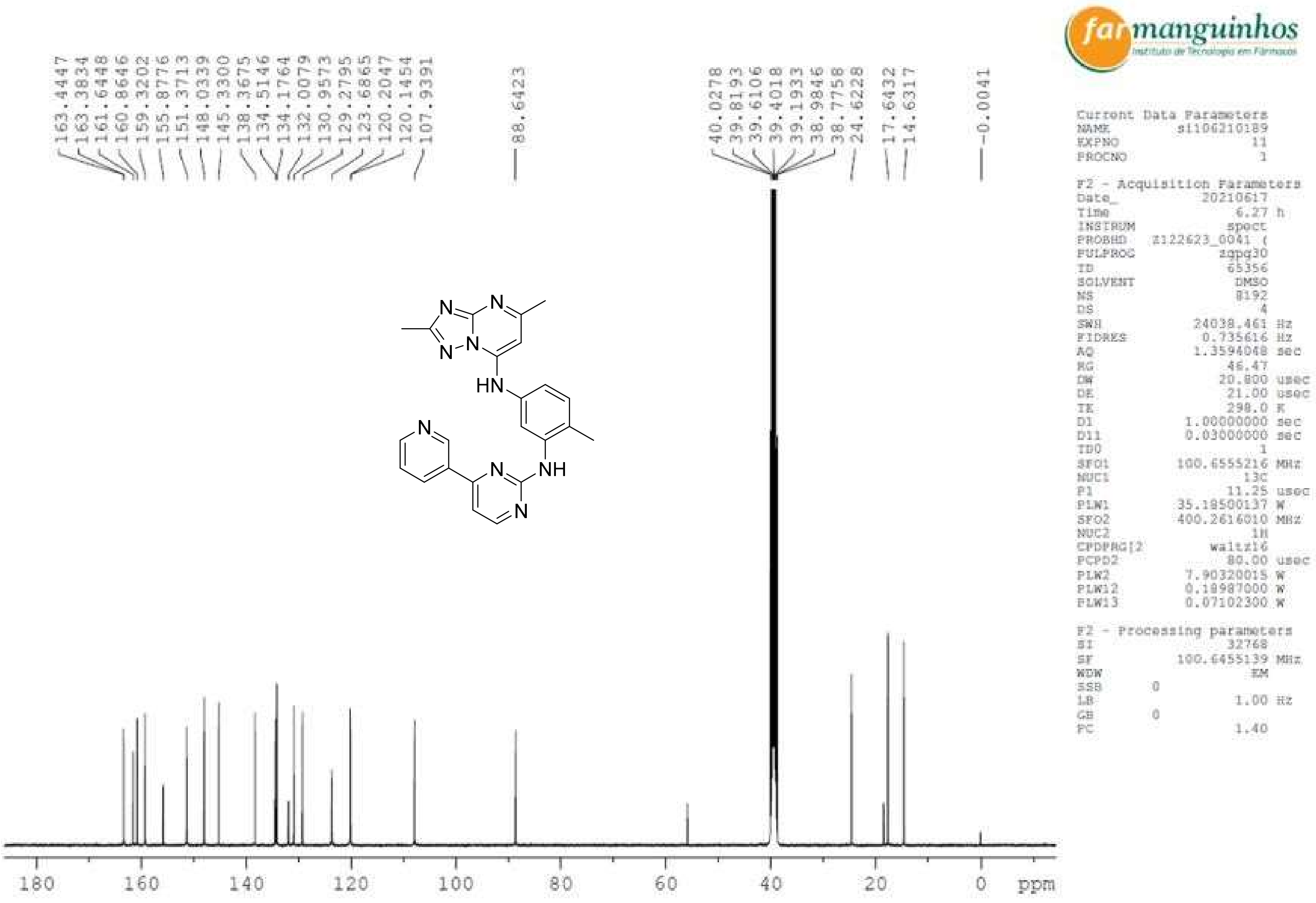

Fig-S.9: ^{13}C NMR of compound **2b**.

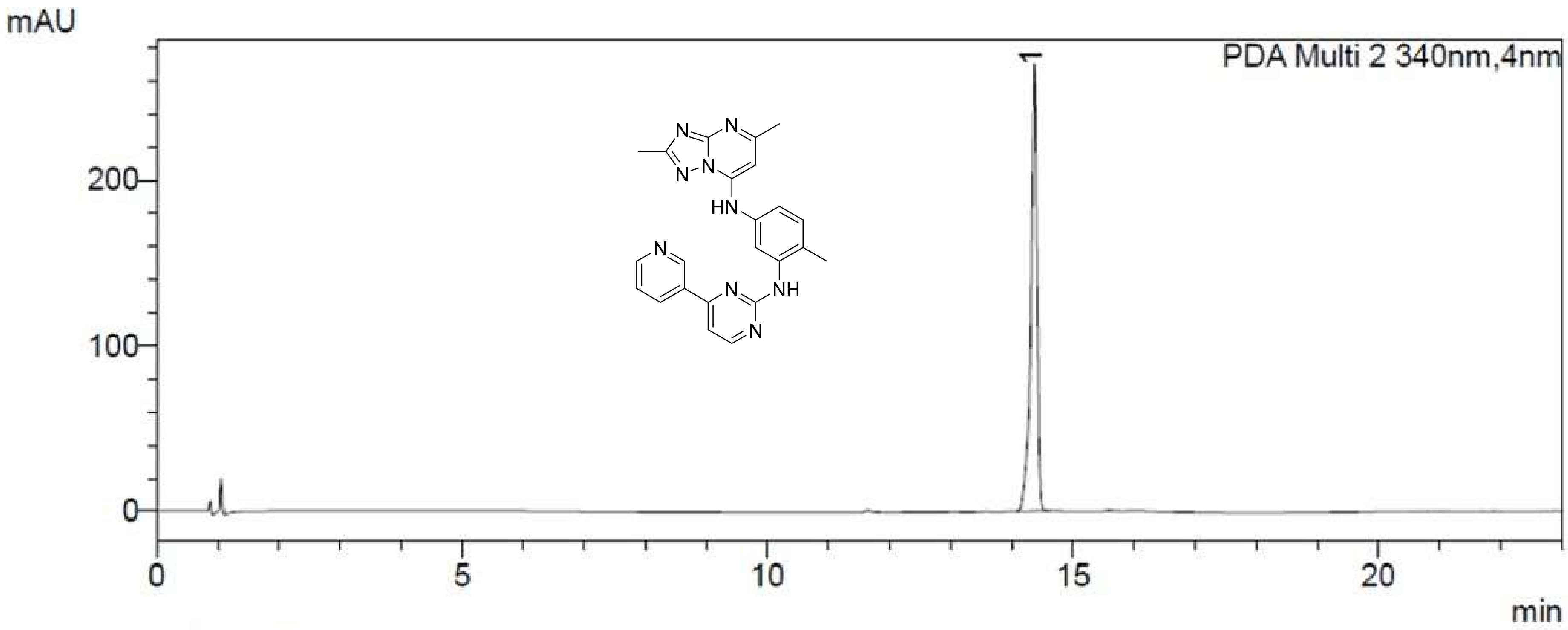

PDA Ch2 340nm

Peak#	Ret. Time	Name	Area	Area%	Theoretical Plates/meter(USP)	Tailing Factor	Resolution(USP)	Capacity Factor(k')
1	14,37		1826770	100,0	626173	0,780	--	--
Total			1826770	100,0				

Fig-S.10: HPLC-DAD of compound **2b**.

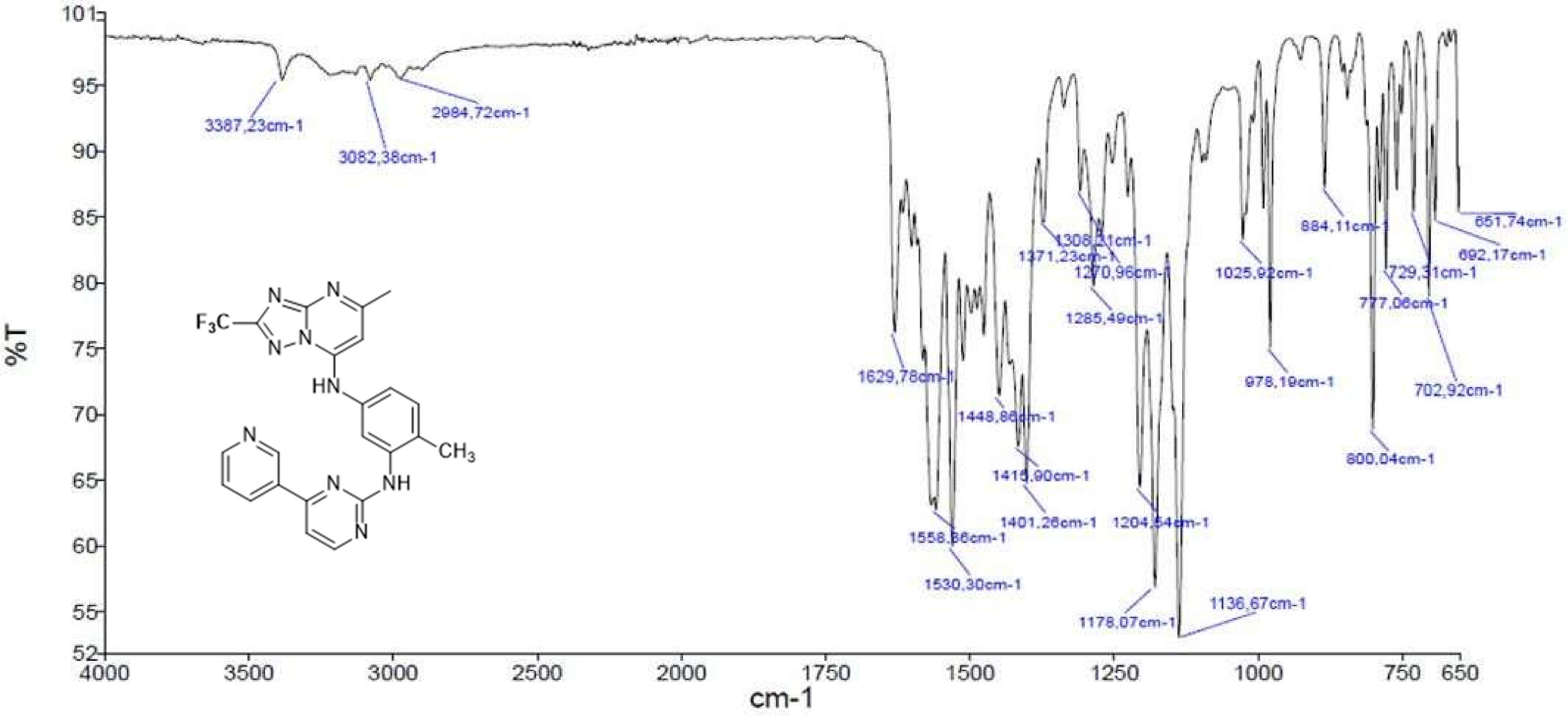

Fig- S.11: FT-IR of compound **2c**.

Acquisition Parameter

Ion Source Type	ESI	Ion Polarity	Negative	Alternating Ion Polarity	on
Mass Range Mode	UltraScan	Scan Begin	100 m/z	Scan End	1000 m/z
Accumulation Time	59 µs	RF Level	71 %	Trap Drive	58.7
SPS Target Mass	500 m/z	Averages	10 Spectra	n/a	n/a

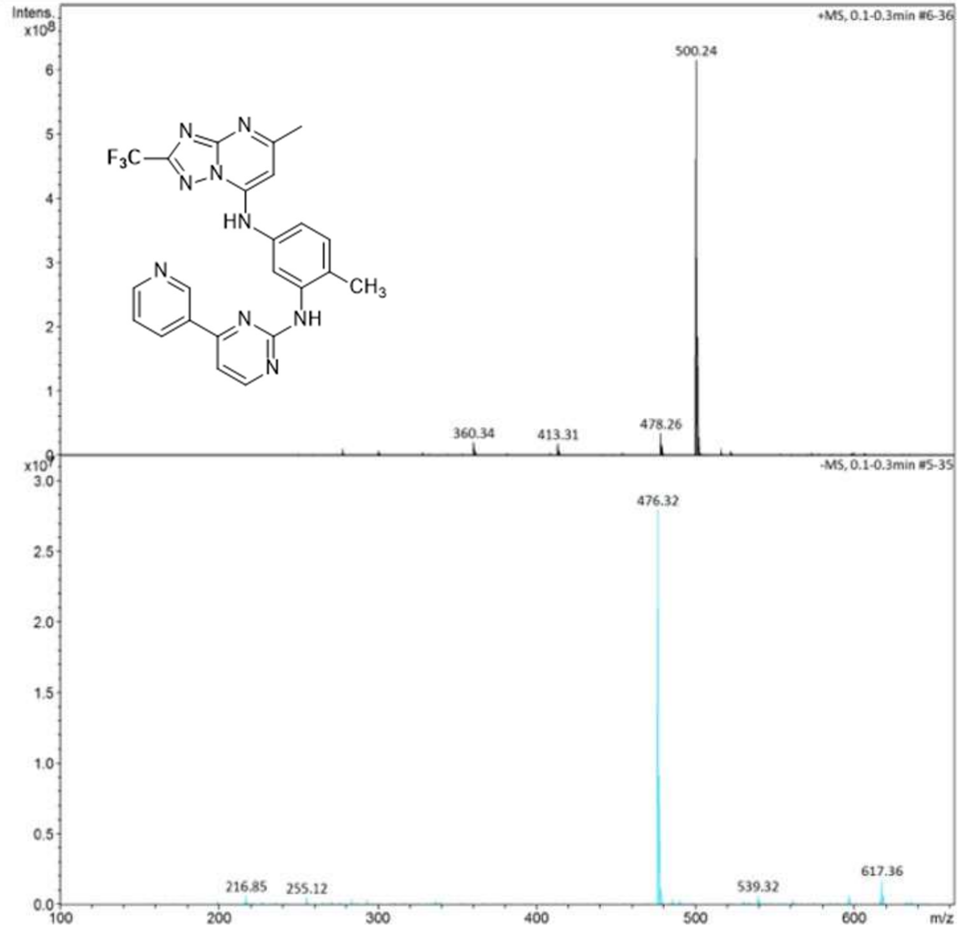

Fig-S.12: ESI-MS of compound **2c**.

+MS, 0.3-0.7min #17-38

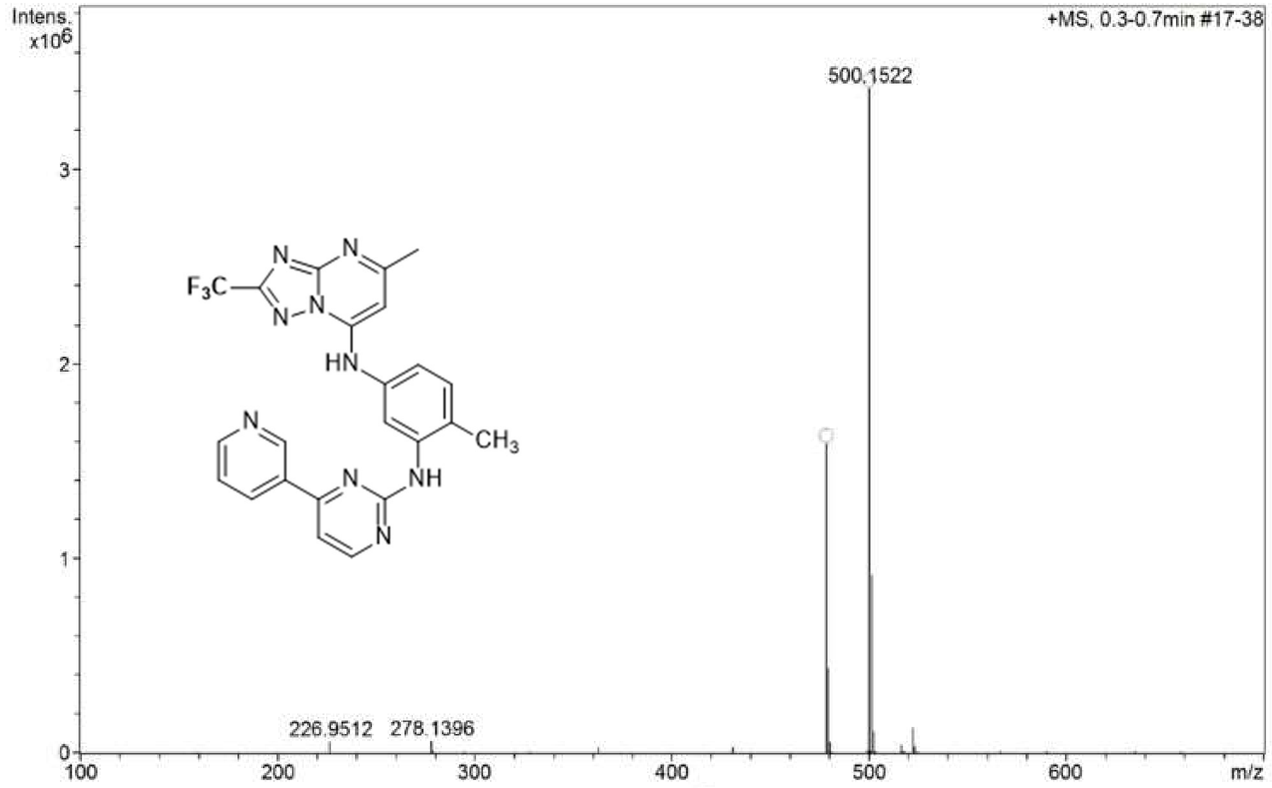

Meas. m/z	#	Ion Formula	m/z	err [ppm]	Mean err [ppm]	rdb	N-Rule	e^- Conf	mSigma	Std I	Std Mean m/z	Std I VarNorm	Std m/z Diff	Std Comb Dev
478.170269	1	C23H19F3N9	478.171003	1.5	1.5	17.5	ok	even	6.6	10.5	n.a.	n.a.	n.a.	n.a.
	2	C22H23F3N5O4	478.169665	-1.3	-1.0	12.5	ok	even	8.0	10.6	n.a.	n.a.	n.a.	n.a.
	3	C21H27F3NO8	478.168328	-4.1	-3.5	7.5	ok	even	21.4	29.8	n.a.	n.a.	n.a.	n.a.
500.152159	1	C23H18F3N9Na	500.152947	1.6	1.7	17.5	ok	even	9.3	15.3	n.a.	n.a.	n.a.	n.a.

Fig-S.13: HRMS of compound **2c**.

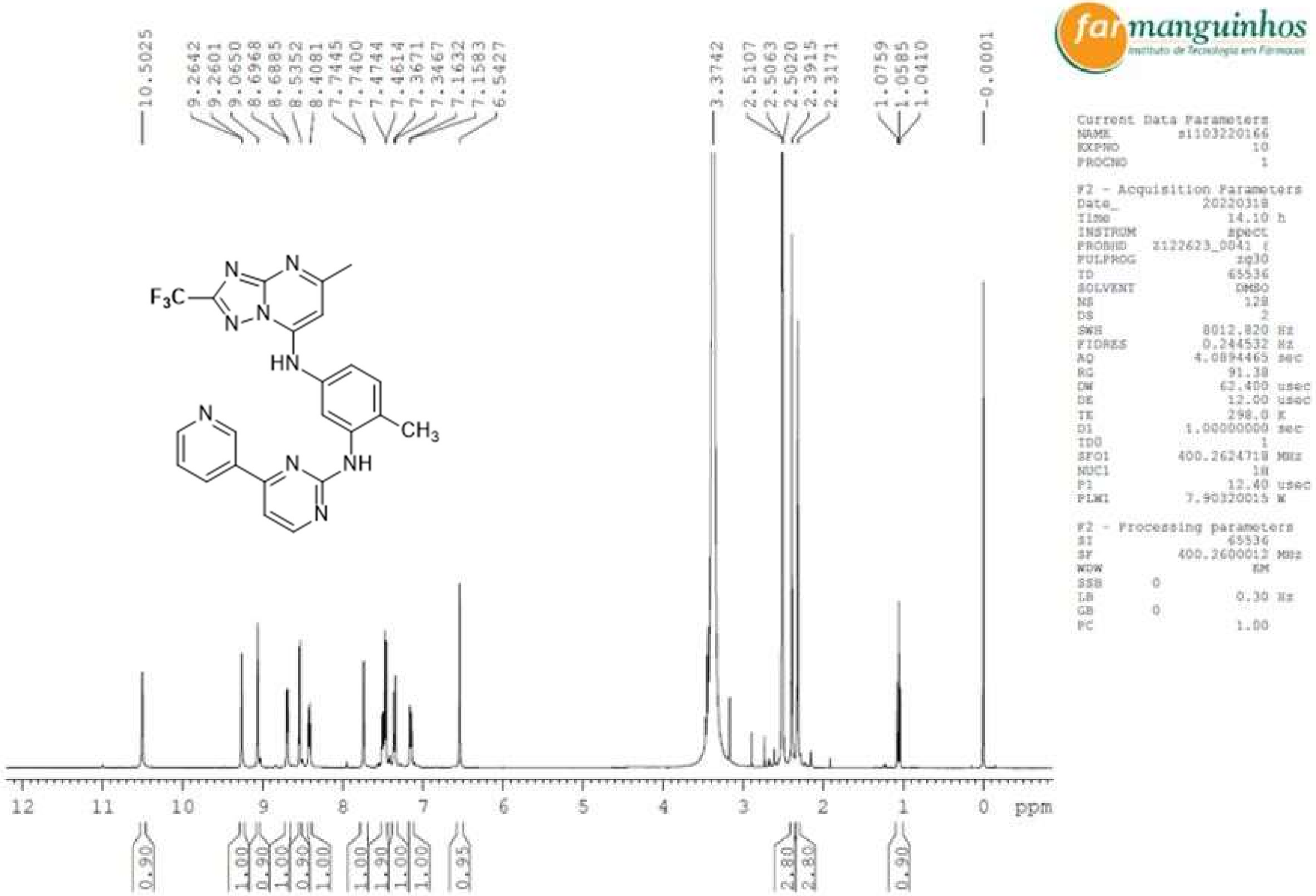

Fig-S.14: ^{1}H NMR of compound **2c**.

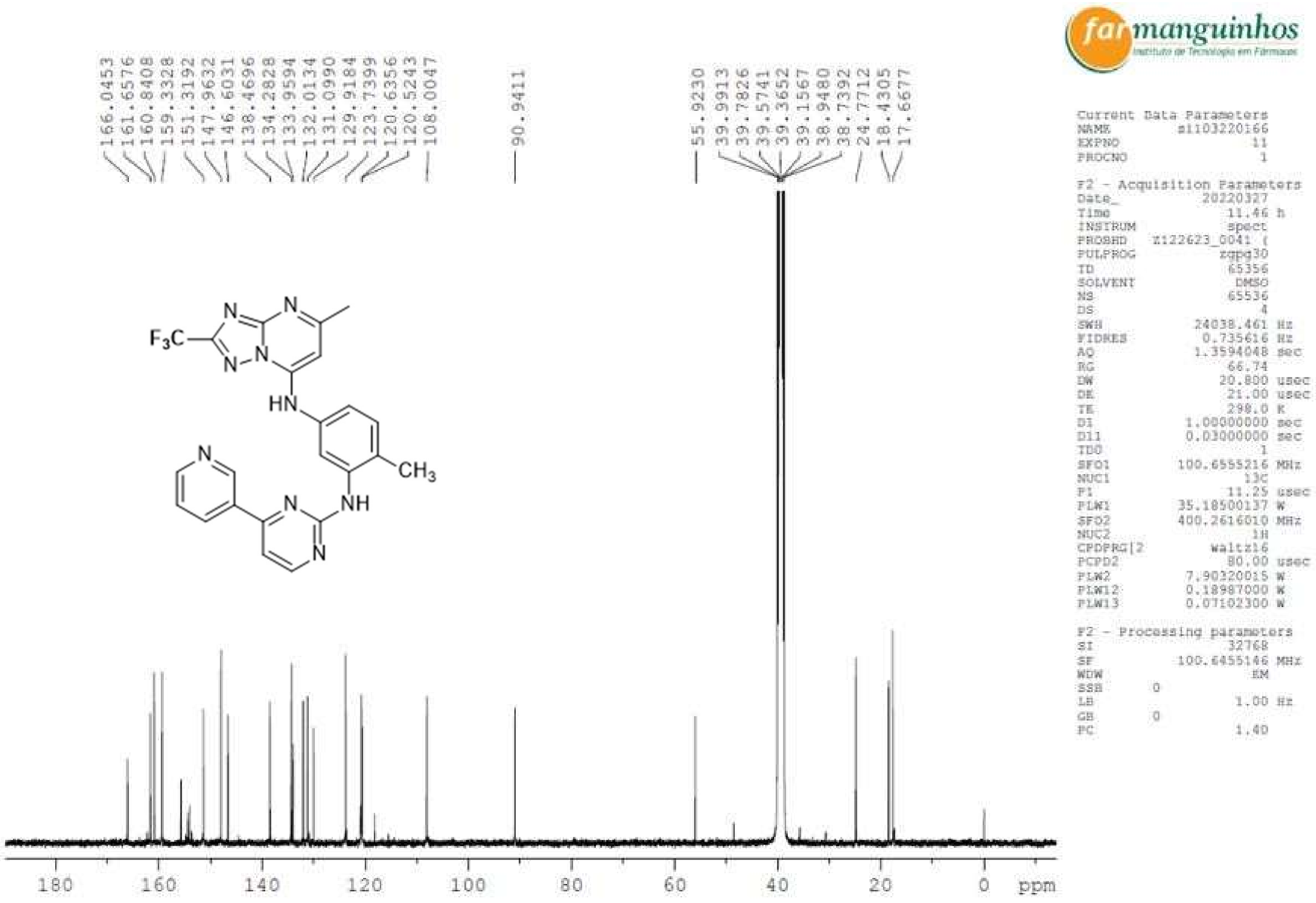

Fig-S.15: ^{13}C NMR of compound **2c**.

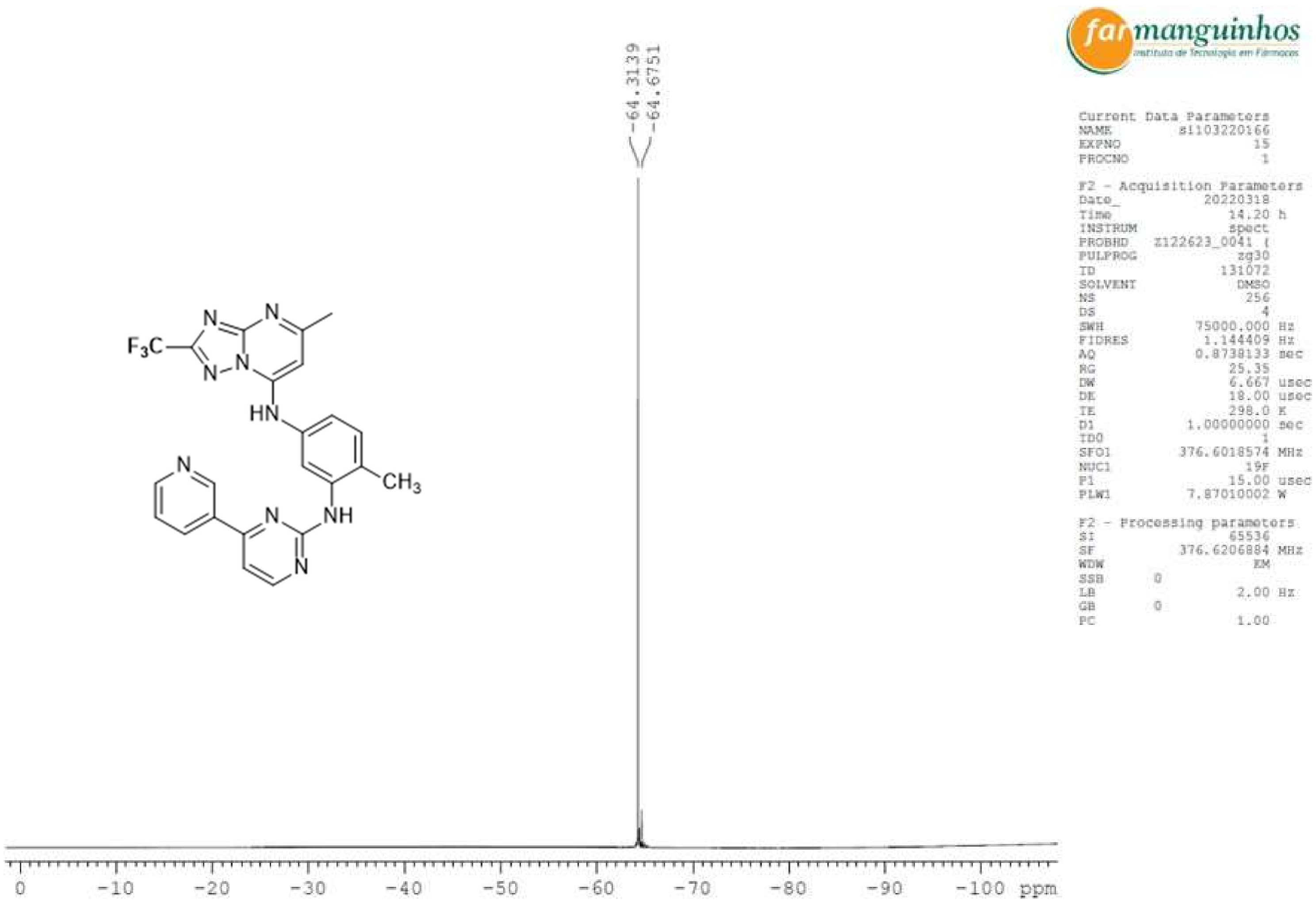

Fig-S.16: ^{19}F NMR of compound **2c**.

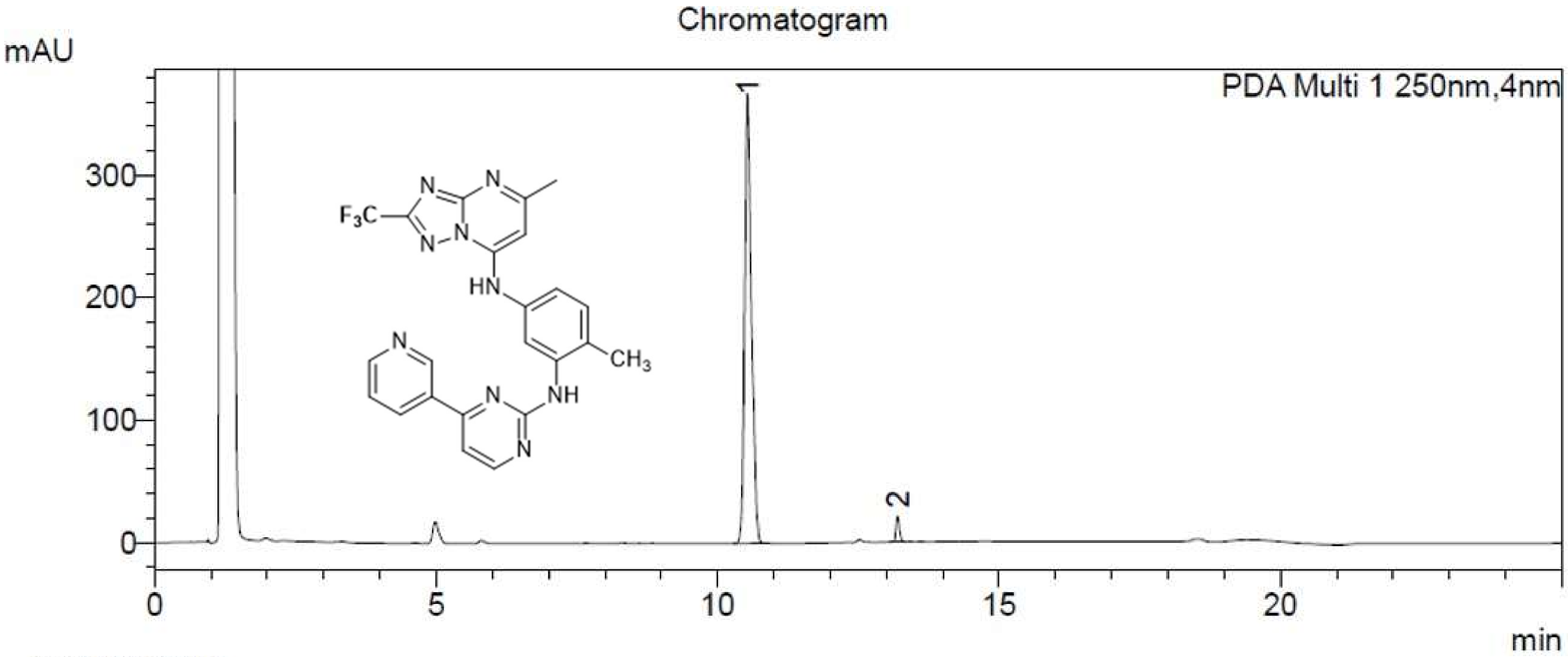

PDA Ch1 250nm

Peak#	Ret. Time	Name	Area	Area%	Theoretical Plates/meter(USP)	Tailing Factor	Resolution(USP)	Capacity Factor(k')
1	10,53		3088438	97,1	212669	1,281	--	--
2	13,20		93874	2,9	1047722	1,228	14,449	0,253
Total			3182312	100,0				

Fig-S.17: HPLC-DAD of compound **2c**.

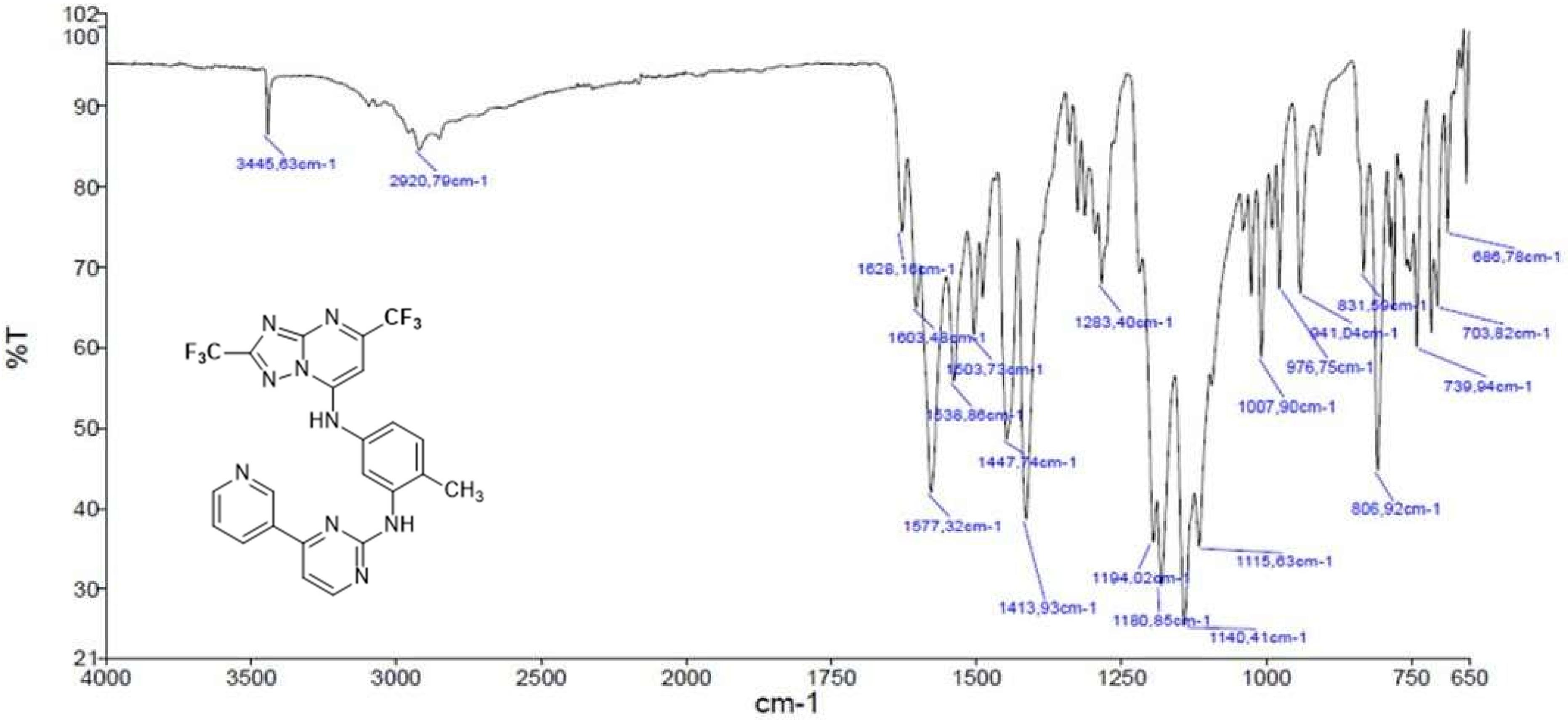

Fig-S.18: FT-IR of compound **2d**.

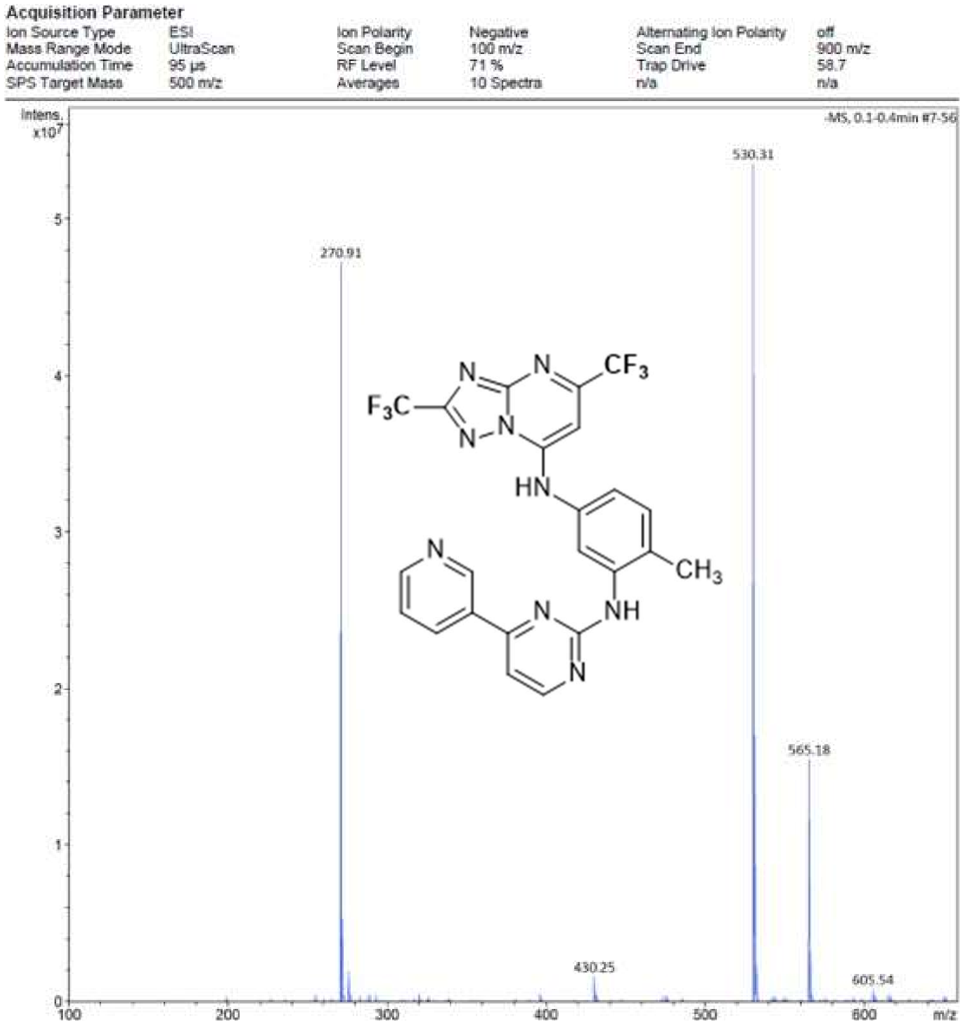

Fig-S.19: ESI-MS of compound **2d**.

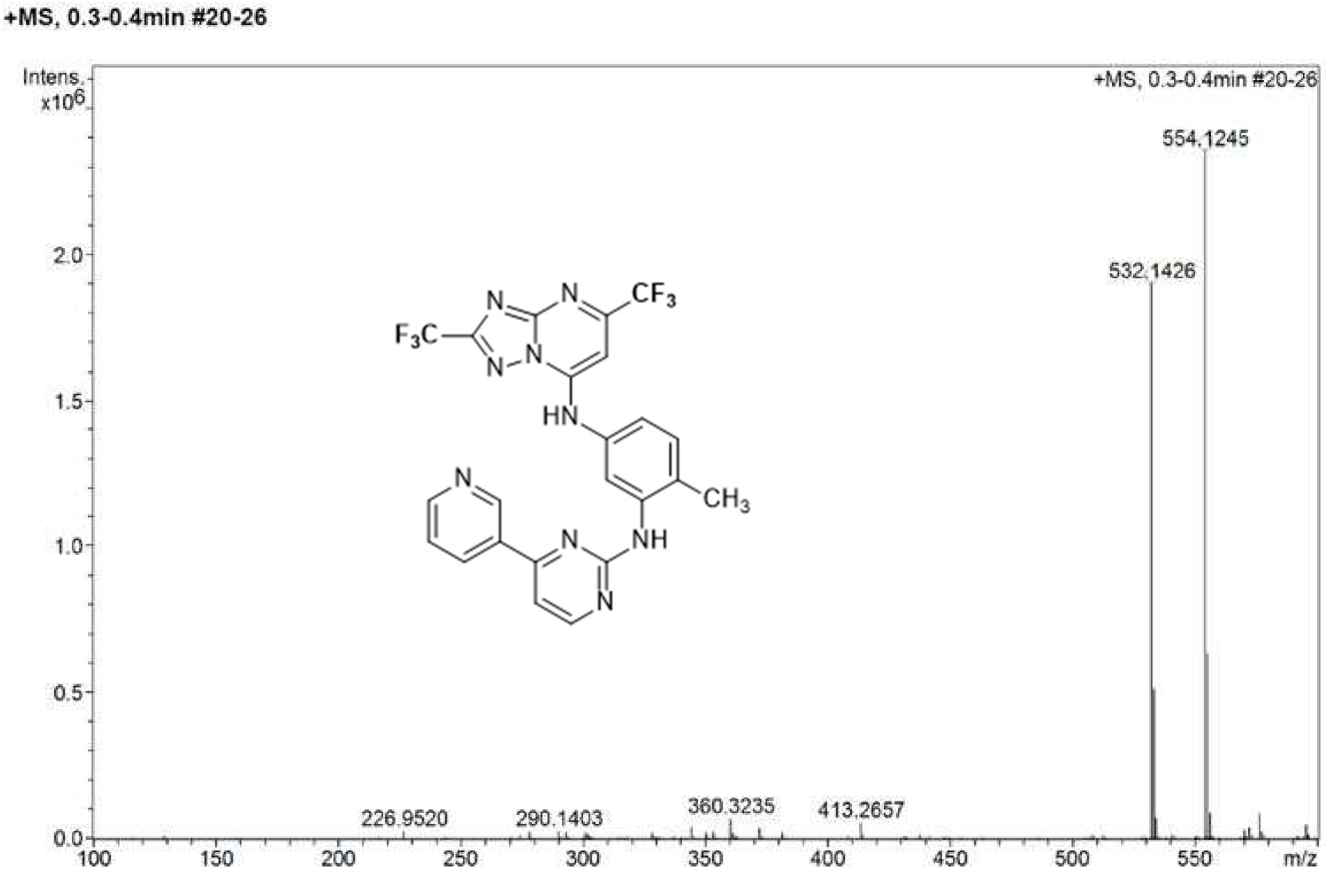

Meas. m/z	#	Ion Formula	m/z	err [ppm]	Mean err [ppm]	rdb	N-Rule	e⁻ Conf	mSigma	Std I	Std Mean m/z	Std I VarNorm	Std m/z Diff	Std Comb Dev
532.142561	1	C23H16F6N9	532.142737	0.3	0.5	17.5	ok	even	6.7	10.1	n.a.	n.a.	n.a.	n.a.
	2	C22H20F6N5O4	532.141400	-2.2	-1.8	12.5	ok	even	7.2	9.7	n.a.	n.a.	n.a.	n.a.
554.124467	1	C23H15F6N9Na	554.124682	0.4	0.8	17.5	ok	even	7.5	11.2	n.a.	n.a.	n.a.	n.a.

Fig-S.20: HRMS of compound **2d**.

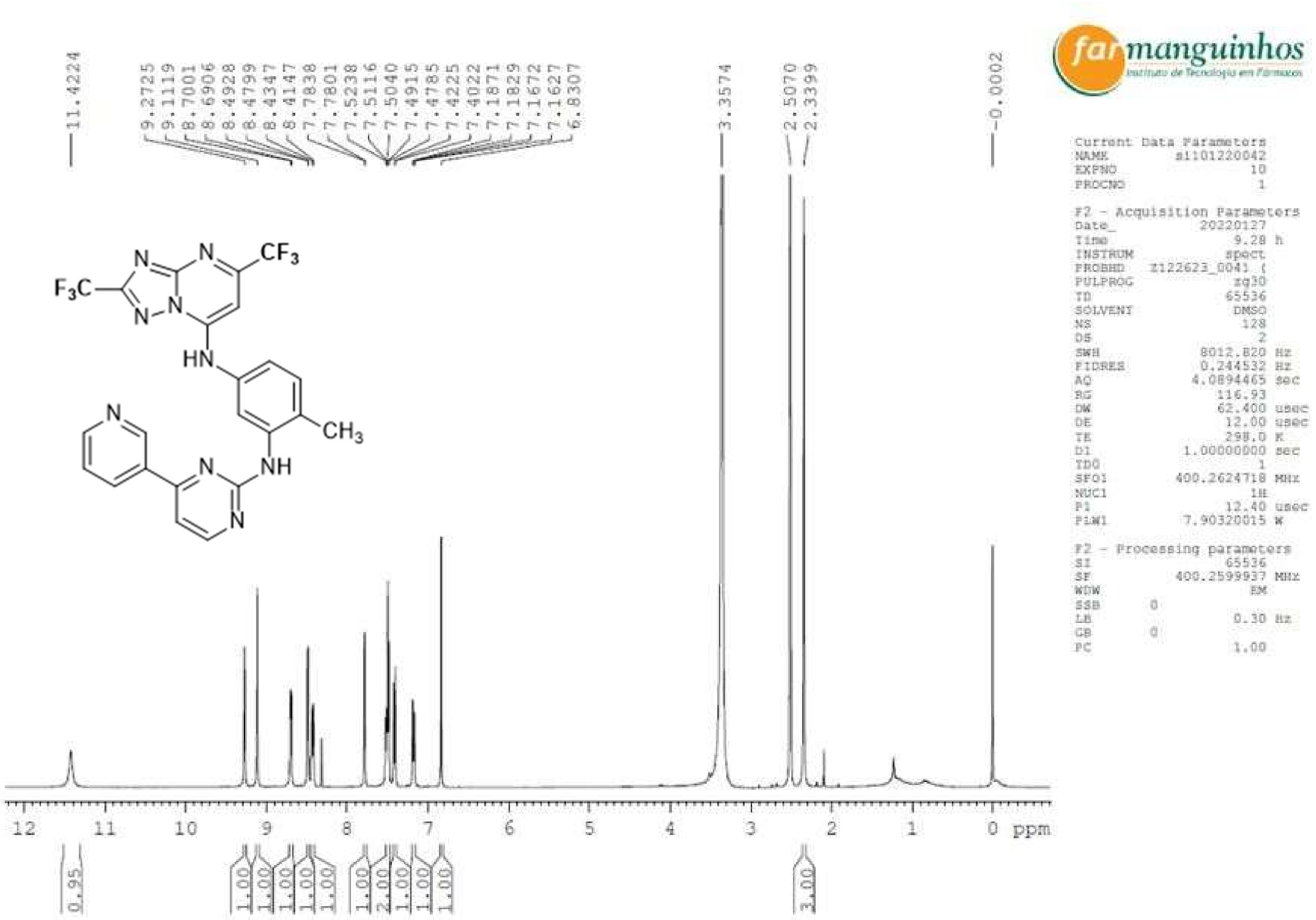

Fig-S.21: ^{1}H NMR of compound **2d**.

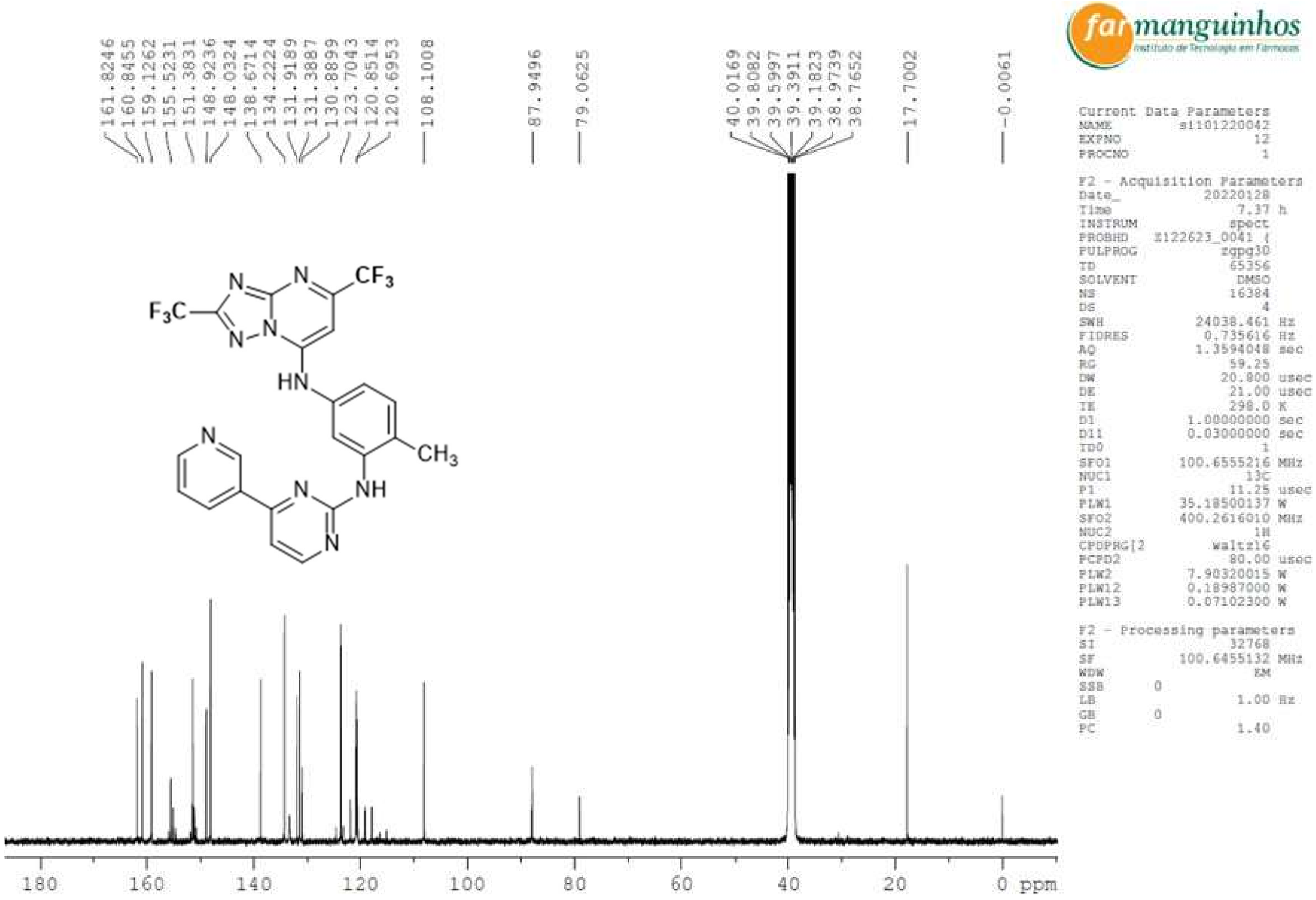

Fig-S.22: ^{13}C NMR of compound **2d**.

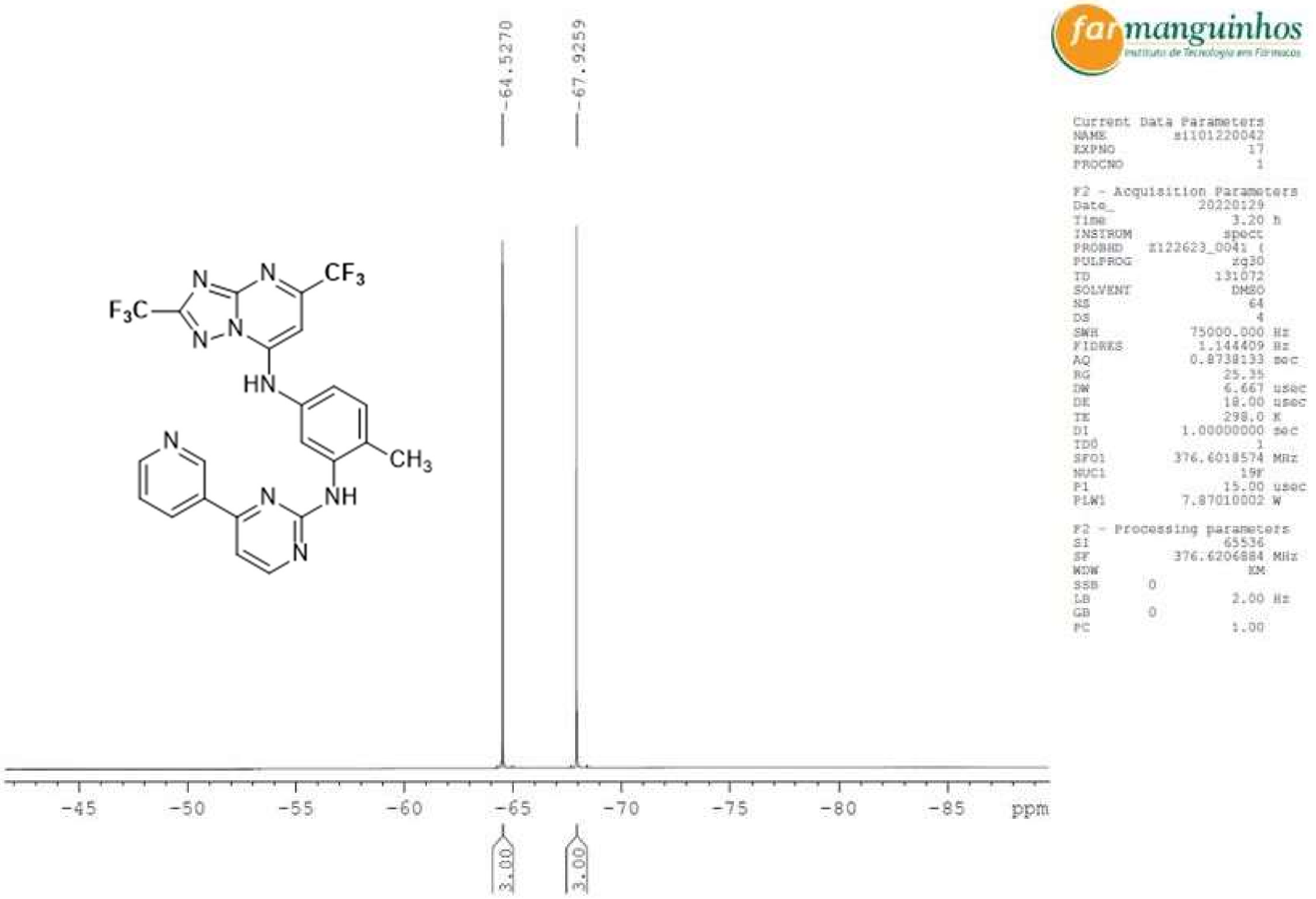

Fig-S.23: ^{19}F NMR of compound **2d**.

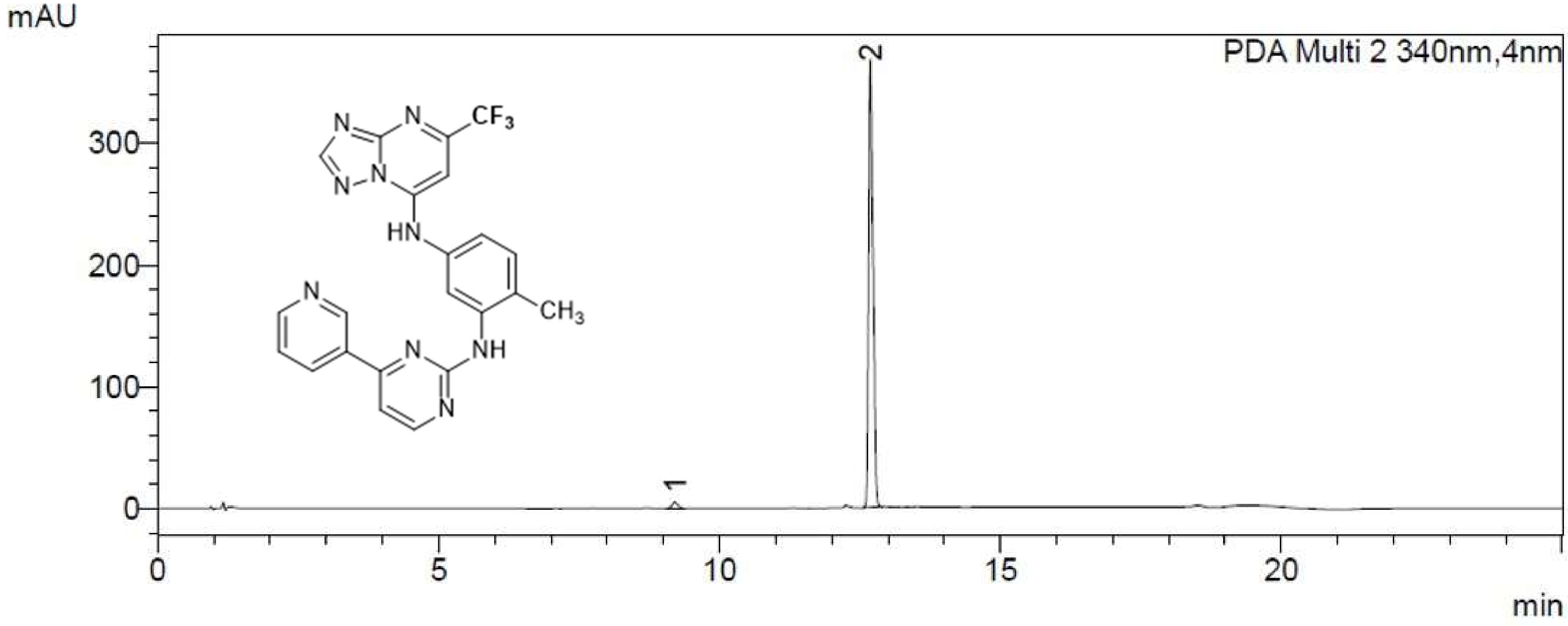

PDA Ch2 340nm

Peak#	Ret. Time	Name	Area	Area%	Theoretical Plates/meter(USP)	Tailing Factor	Resolution(USP)	Capacity Factor(k')
1	9,20		36334	1,8	208532	1,077	--	--
2	12,68		1993511	98,2	604028	1,399	18,468	0,378
Total			2029845	100,0				

Fig-S.24: HPLC-DAD of compound **2d**.

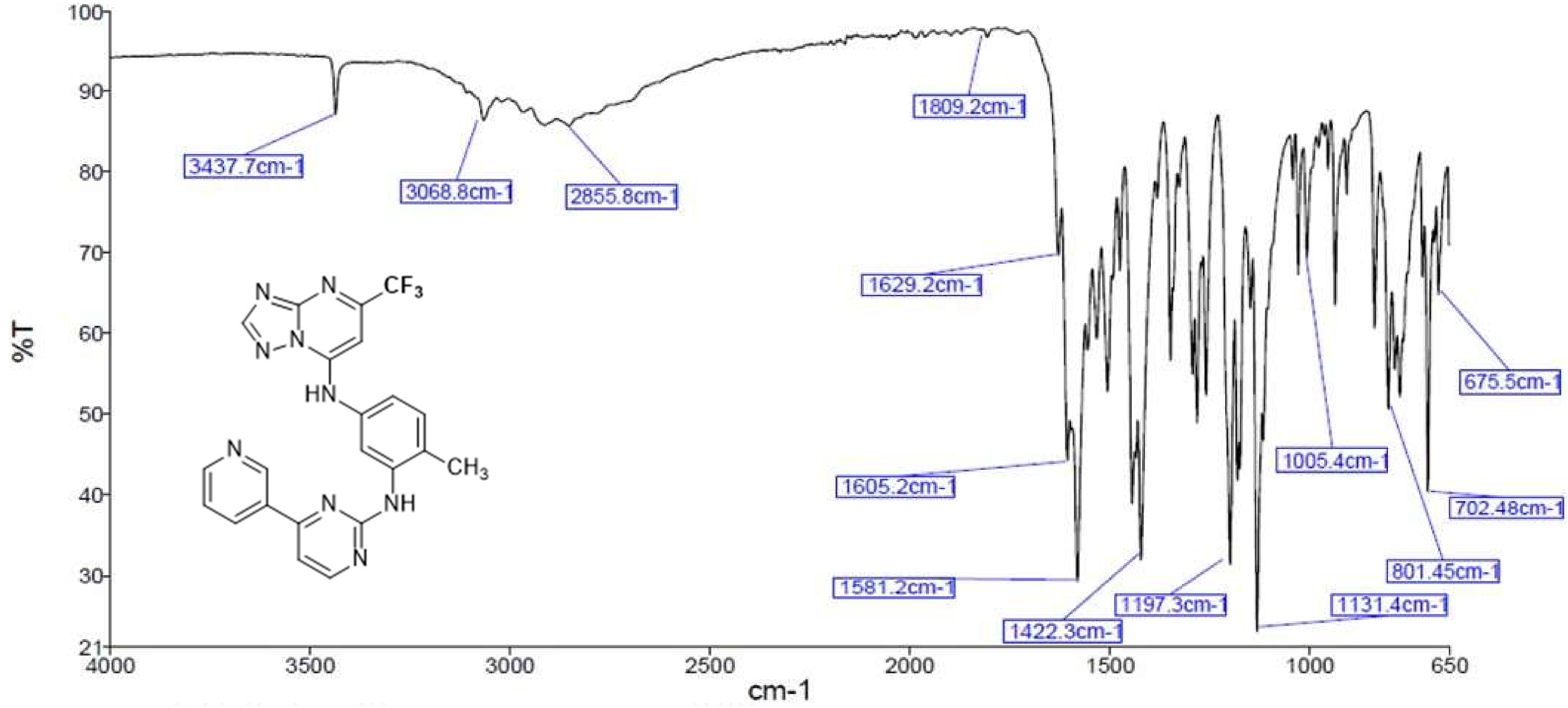

Fig-S.25: FT-IR of compound **2e**.

Acquisition Parameter

Ion Source Type	ESI	Ion Polarity	Positive	Alternating Ion Polarity	off		
Mass Range Mode	UltraScan	Scan Begin	100 m/z	Scan End	800 m/z		
Accumulation Time	20 µs	RF Level	67 %	Trap Drive	52.5		
SPS Target Mass	450 m/z	Averages	10 Spectra	n/a	n/a		

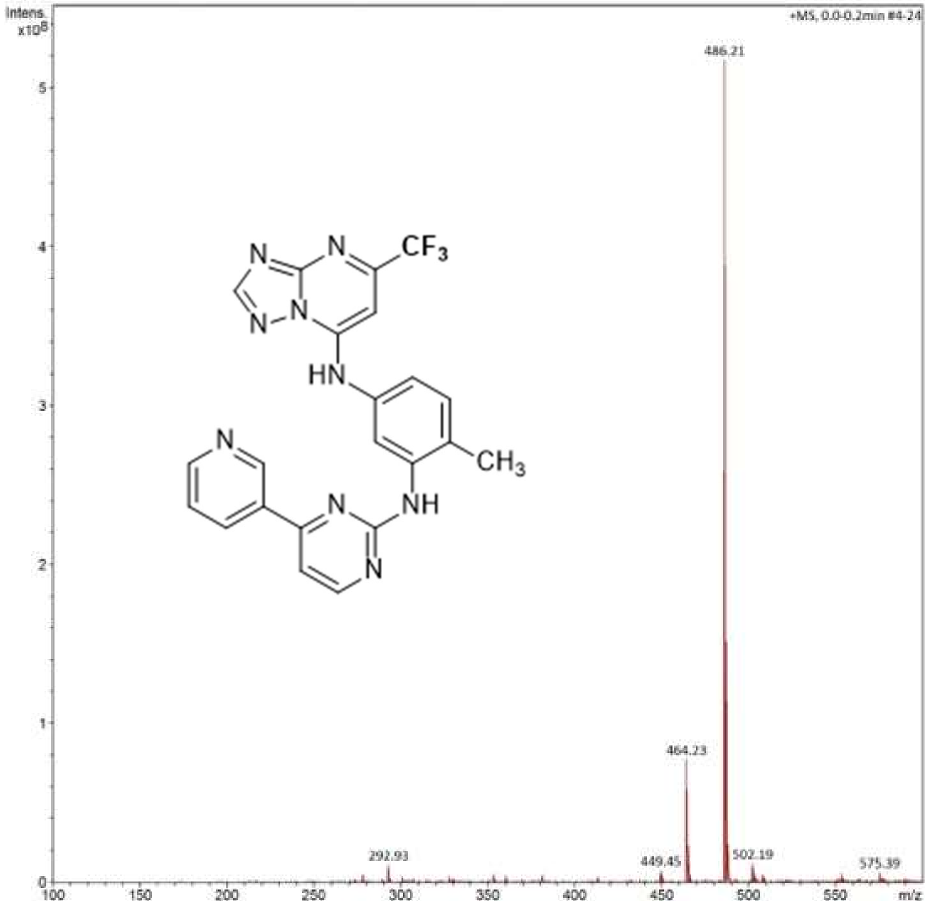

Fig-S.26: ESI-MS of compound **2e**.

+MS, 0.3min #16

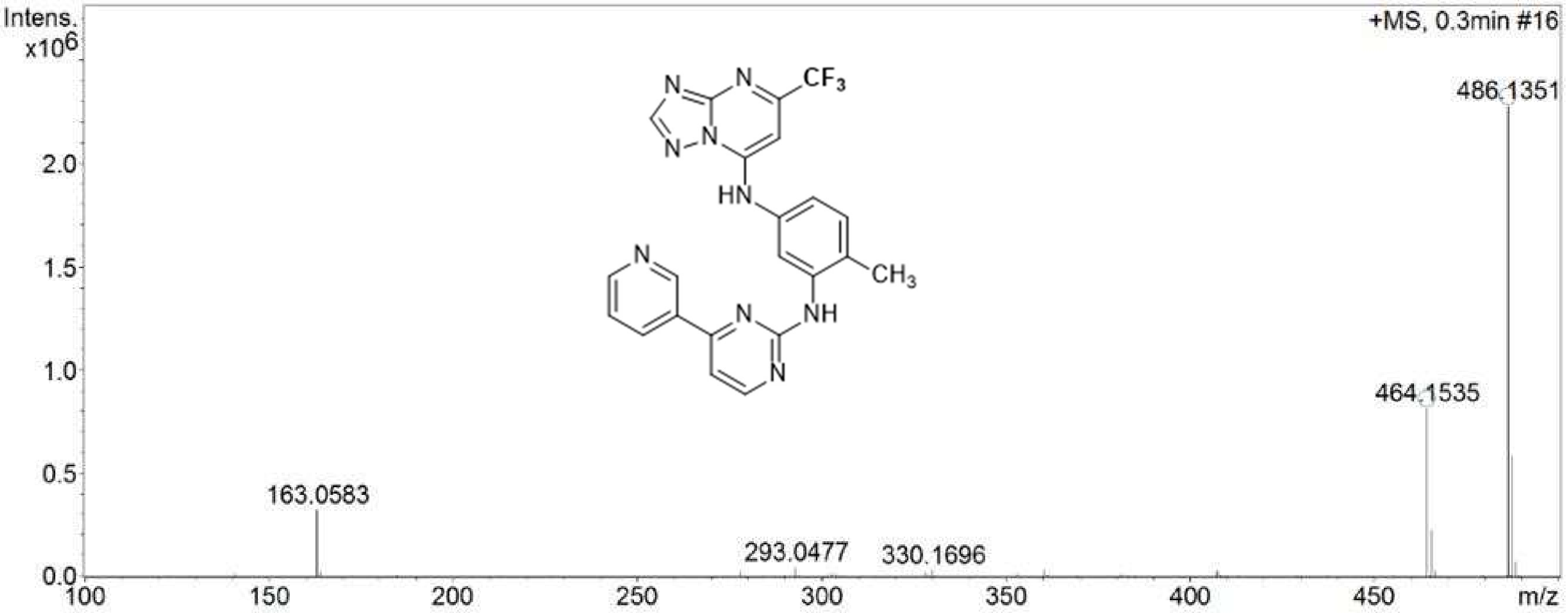

Meas. m/z	#	Ion Formula	m/z	err [ppm]	Mean err [ppm]	rdb	N-Rule	e⁻ Conf	mSigma	Std I	Std Mean m/z	Std I VarNorm	Std m/z Diff	Std Comb Dev
464.153454	1	C22H17F3N9	464.155353	4.1	4.0	17.5	ok	even	1.6	2.5	n.a.	n.a.	n.a.	n.a.
	2	C21H21F3N5O4	464.154015	1.2	1.4	12.5	ok	even	15.1	21.9	n.a.	n.a.	n.a.	n.a.
	3	C20H25F3NO8	464.152678	-1.7	-1.2	7.5	ok	even	28.7	42.0	n.a.	n.a.	n.a.	n.a.
486.135134	1	C21H20F3N5NaO4	486.135960	1.7	1.7	12.5	ok	even	6.3	8.3	n.a.	n.a.	n.a.	n.a.
	2	C22H16F3N9Na	486.137297	4.5	4.2	17.5	ok	even	8.8	14.3	n.a.	n.a.	n.a.	n.a.
	3	C17H16F3N11NaO2	486.133274	-3.8	-4.6	13.5	ok	even	18.2	28.8	n.a.	n.a.	n.a.	n.a.
	4	C20H24F3NNaO8	486.134622	-1.1	-0.8	7.5	ok	even	19.3	27.1	n.a.	n.a.	n.a.	n.a.

Fig-S.27: HRMS of compound **2e**.

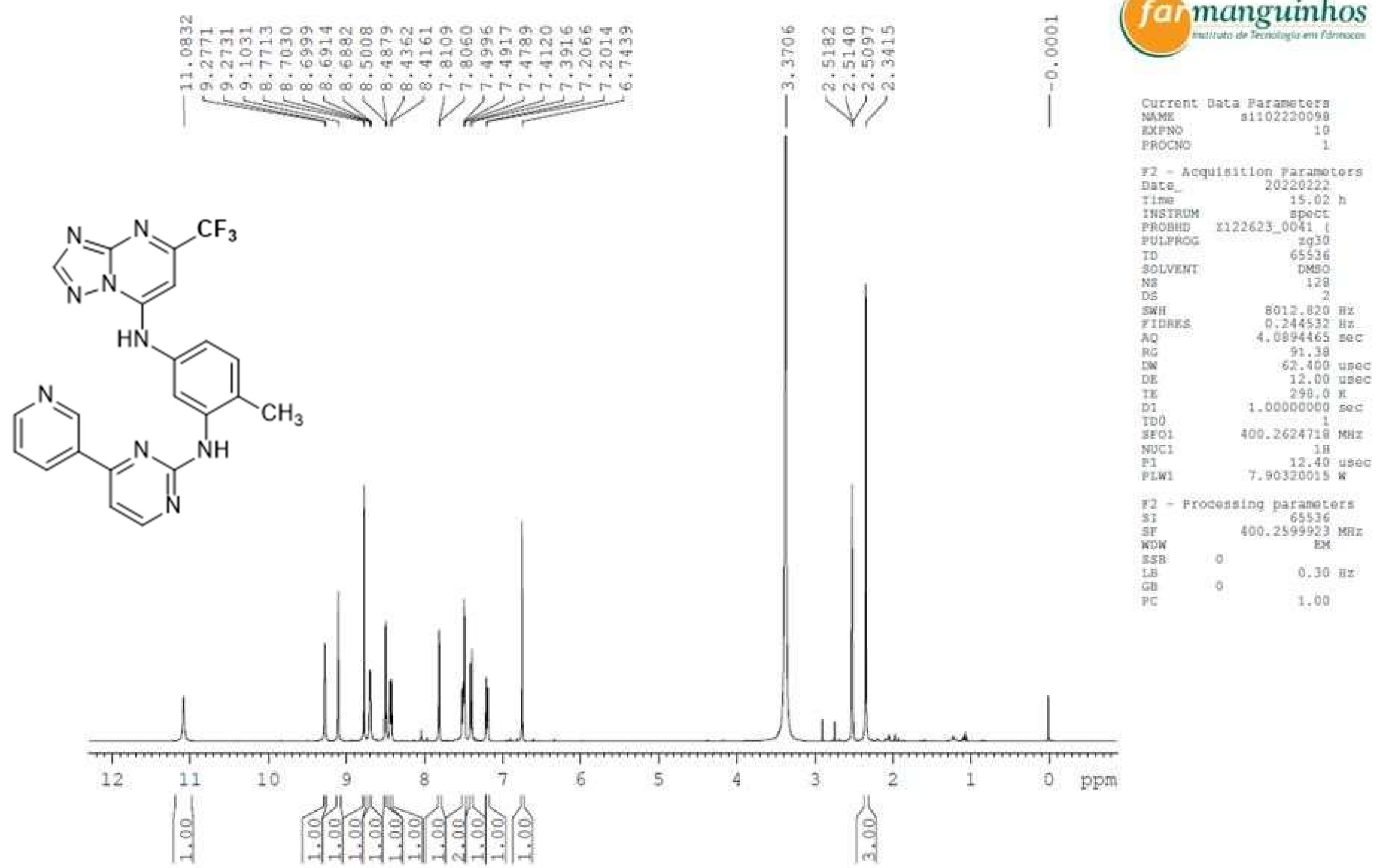

Fig-S.28: ^{1}H NMR of compound **2e**.

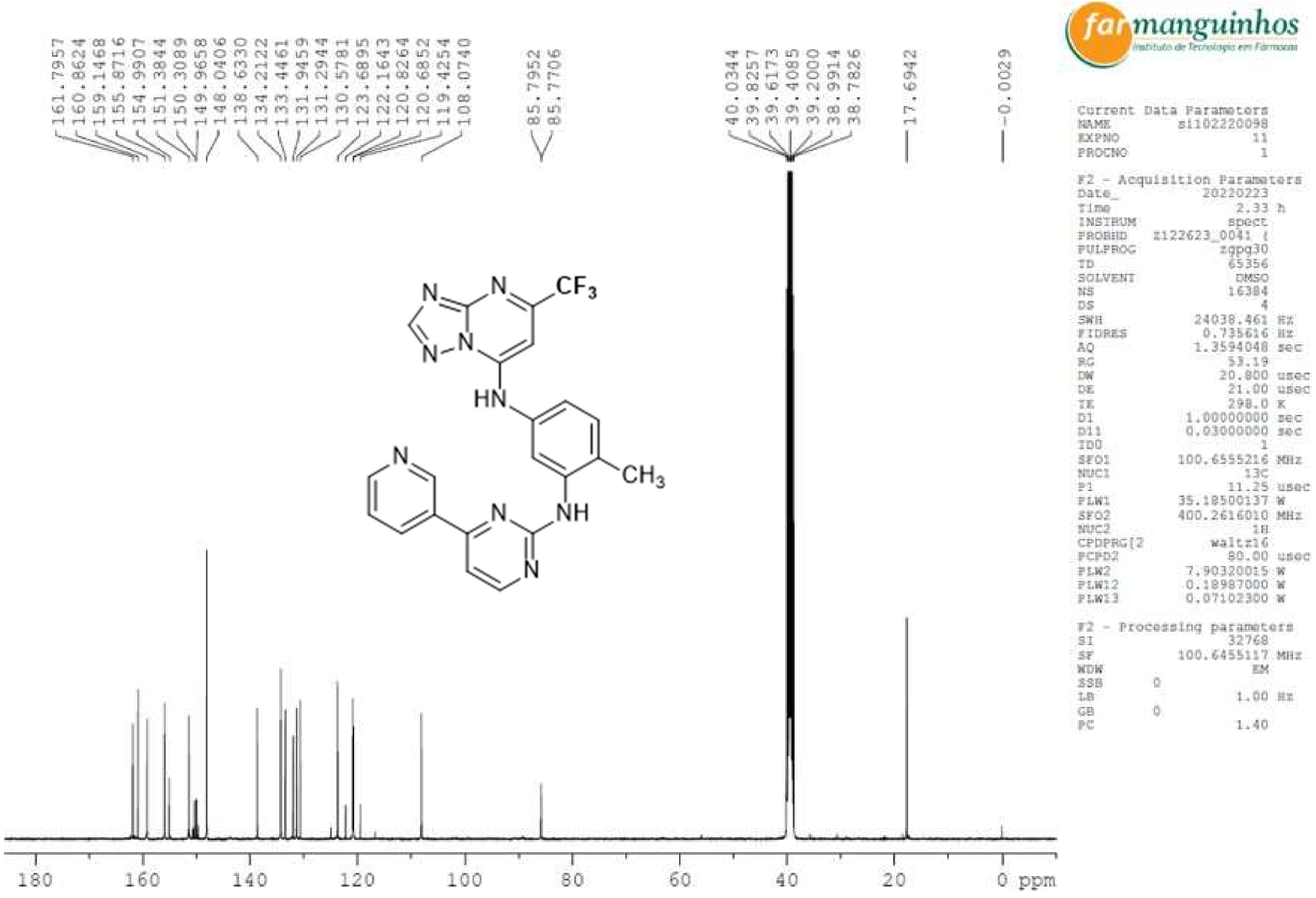

Fig-S.29: ^{13}C NMR of compound **2e**.

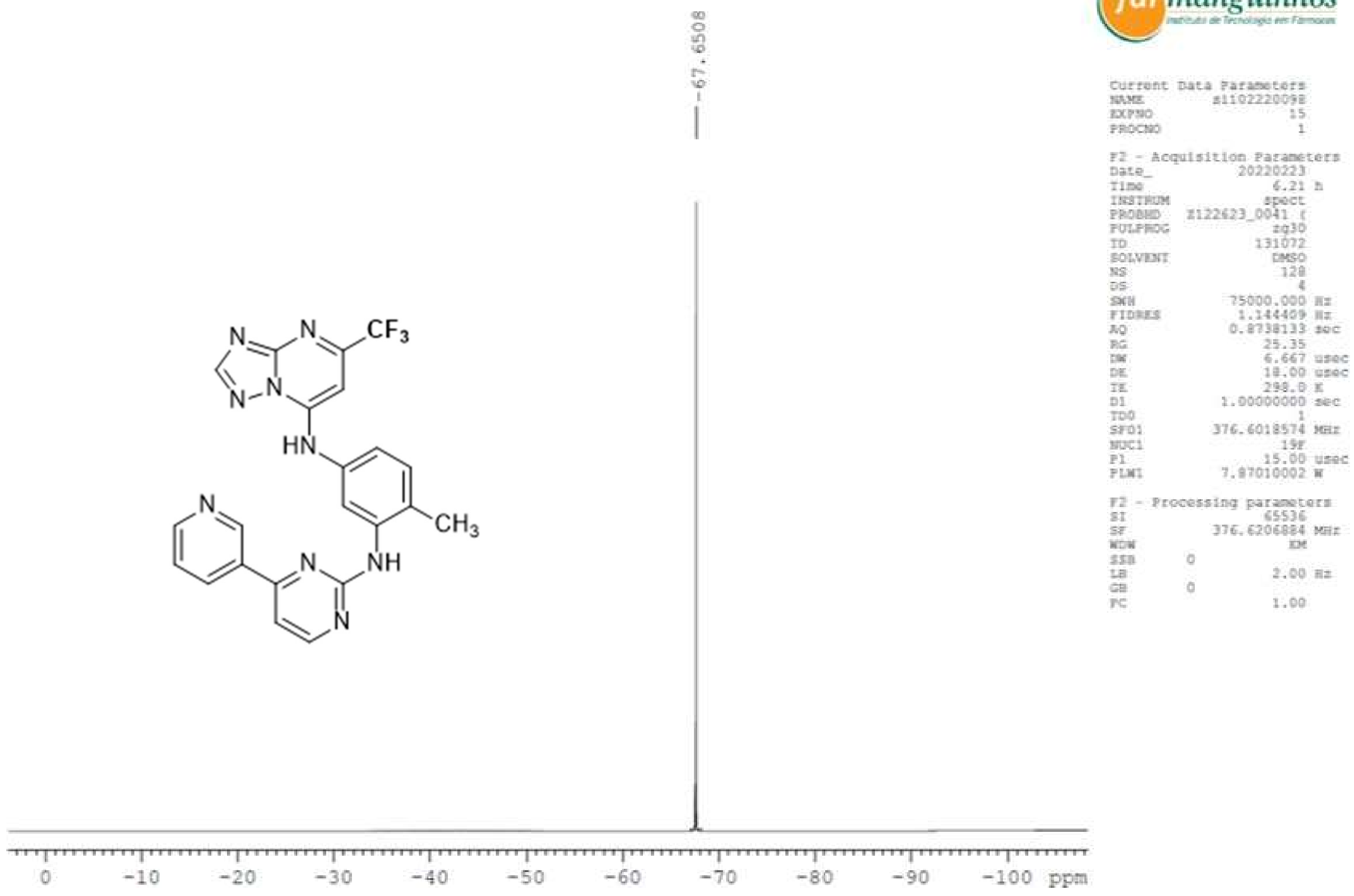

Fig-S.30: ^{19}F NMR of compound **2e**.

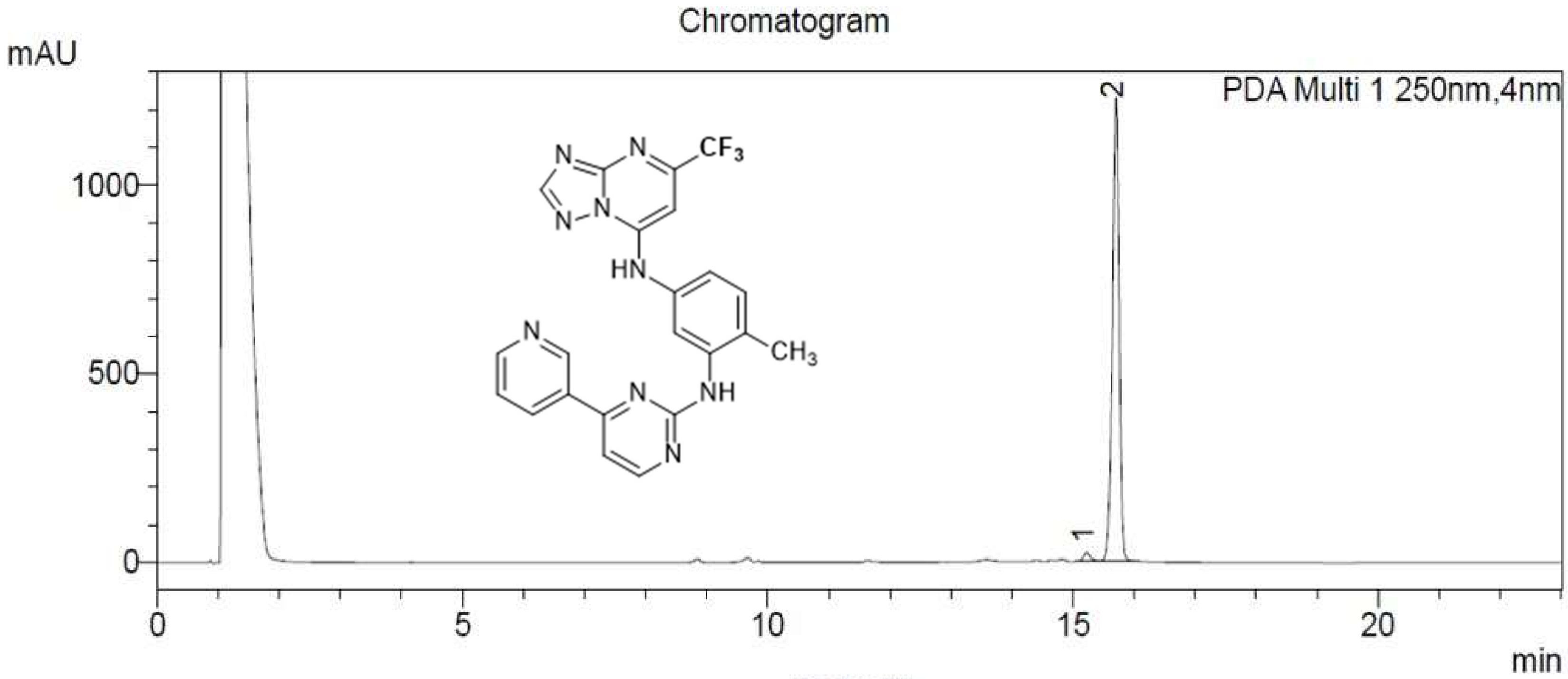

Peak Table

PDA Ch1 250nm

Peak#	Ret. Time	Name	Area	Area%	Theoretical Plates/meter(USP)	Tailing Factor	Resolution(USP)	Capacity Factor(k')
1	15,23		181323	1,9	497611	1,332	--	--
2	15,71		9298308	98,1	576216	0,914	2,193	0,031
Total			9479631	100,0				

Fig-S.31: HPLC-DAD of compound **2e**.

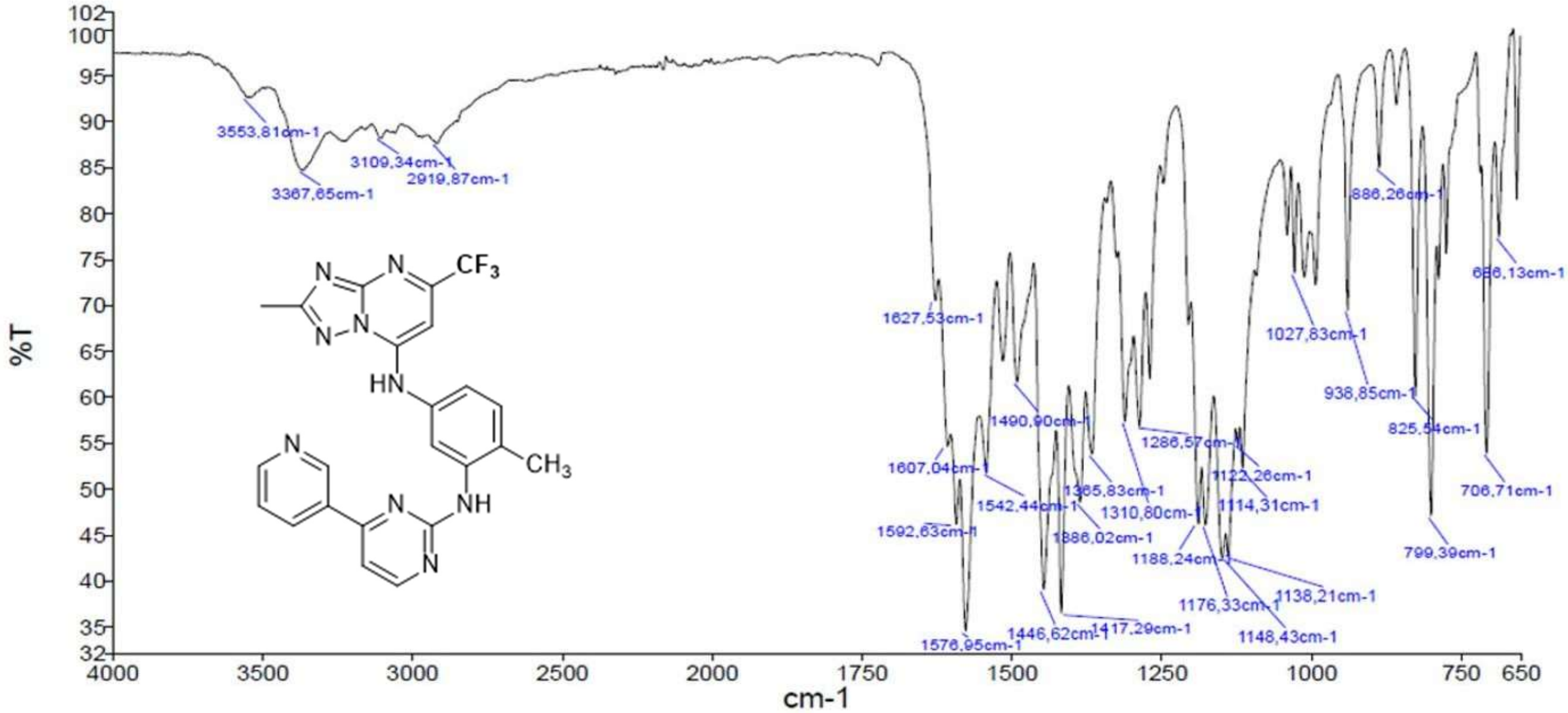

Fig-S.32: FT-IR of compound **2f**.

Acquisition Parameter

Ion Source Type	ESI	Ion Polarity	Positive	Alternating Ion Polarity	on
Mass Range Mode	UltraScan	Scan Begin	100 m/z	Scan End	800 m/z
Accumulation Time	36 µs	RF Level	63 %	Trap Drive	50.5
SPS Target Mass	400 m/z	Averages	10 Spectra	n/a	n/a

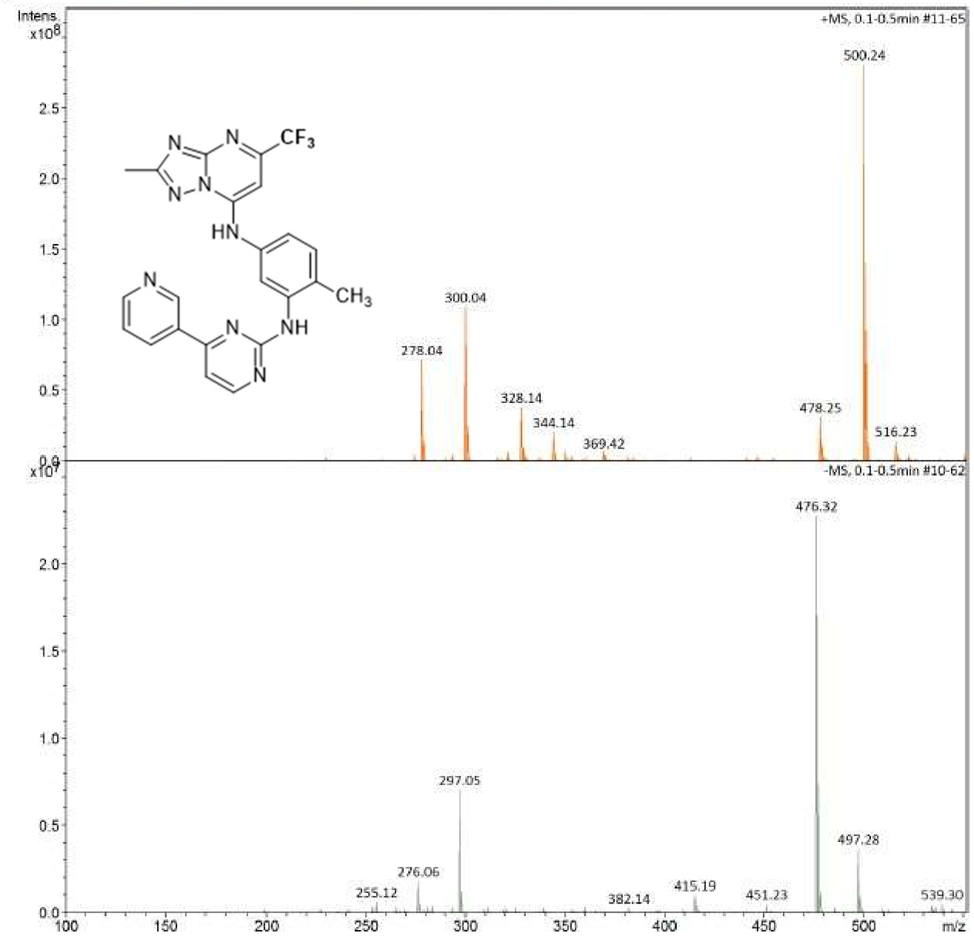

Fig-S.33: ESI-MS of compound **2f**.

+MS, 0.5min #31

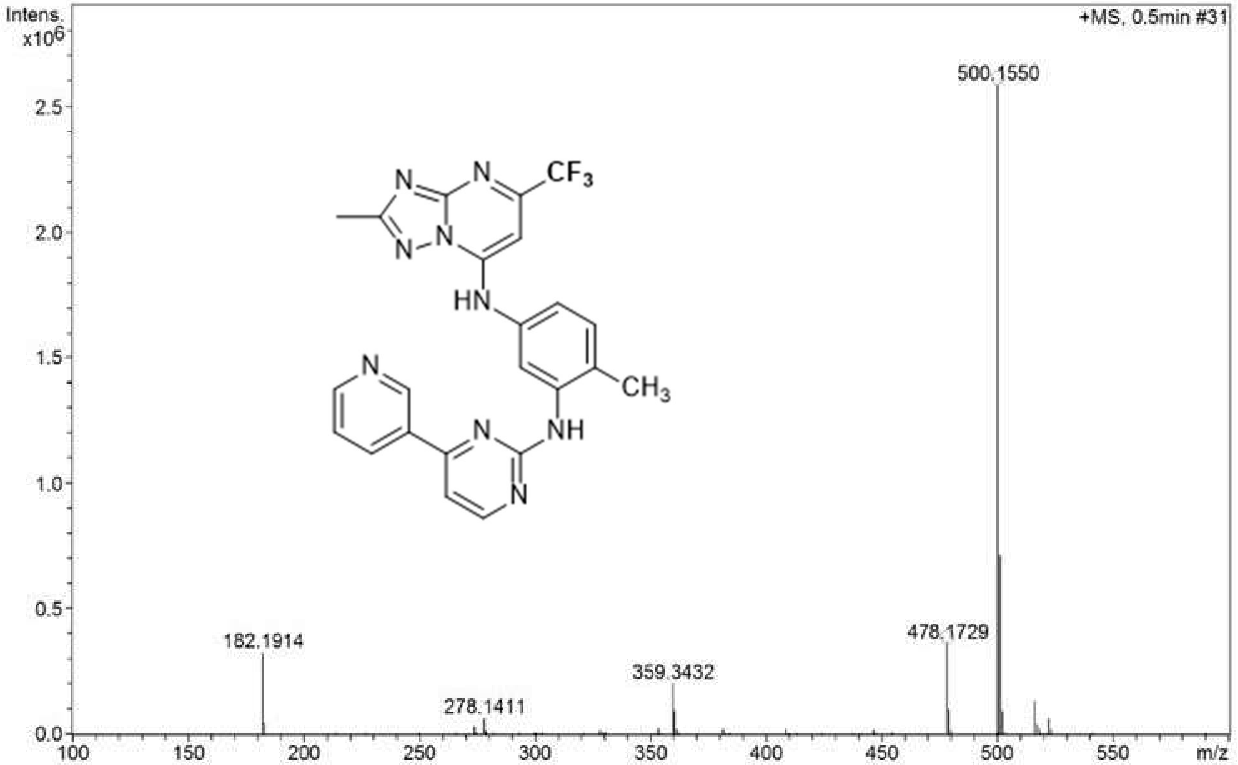

Meas. m/z	#	Ion Formula	m/z	err [ppm]	Mean err [ppm]	rdb	N-Rule	e⁻ Conf	mSigma	Std I	Std Mean m/z	Std I VarNorm	Std m/z Diff	Std Comb Dev
478.172927	1	C23H19F3N9	478.171003	-4.0	-4.5	17.5	ok	even	6.1	8.1	n.a.	n.a.	n.a.	n.a.
	2	C27H23F3N3O2	478.173688	1.6	1.8	16.5	ok	even	19.3	29.2	n.a.	n.a.	n.a.	n.a.
500.154978	1	C23H18F3N9Na	500.152947	-4.1	-4.1	17.5	ok	even	4.2	7.0	n.a.	n.a.	n.a.	n.a.
	2	C27H22F3N3NaO2	500.155632	1.3	1.9	16.5	ok	even	18.6	29.8	n.a.	n.a.	n.a.	n.a.

Fig-S.34: HRMS of compound **2f**.

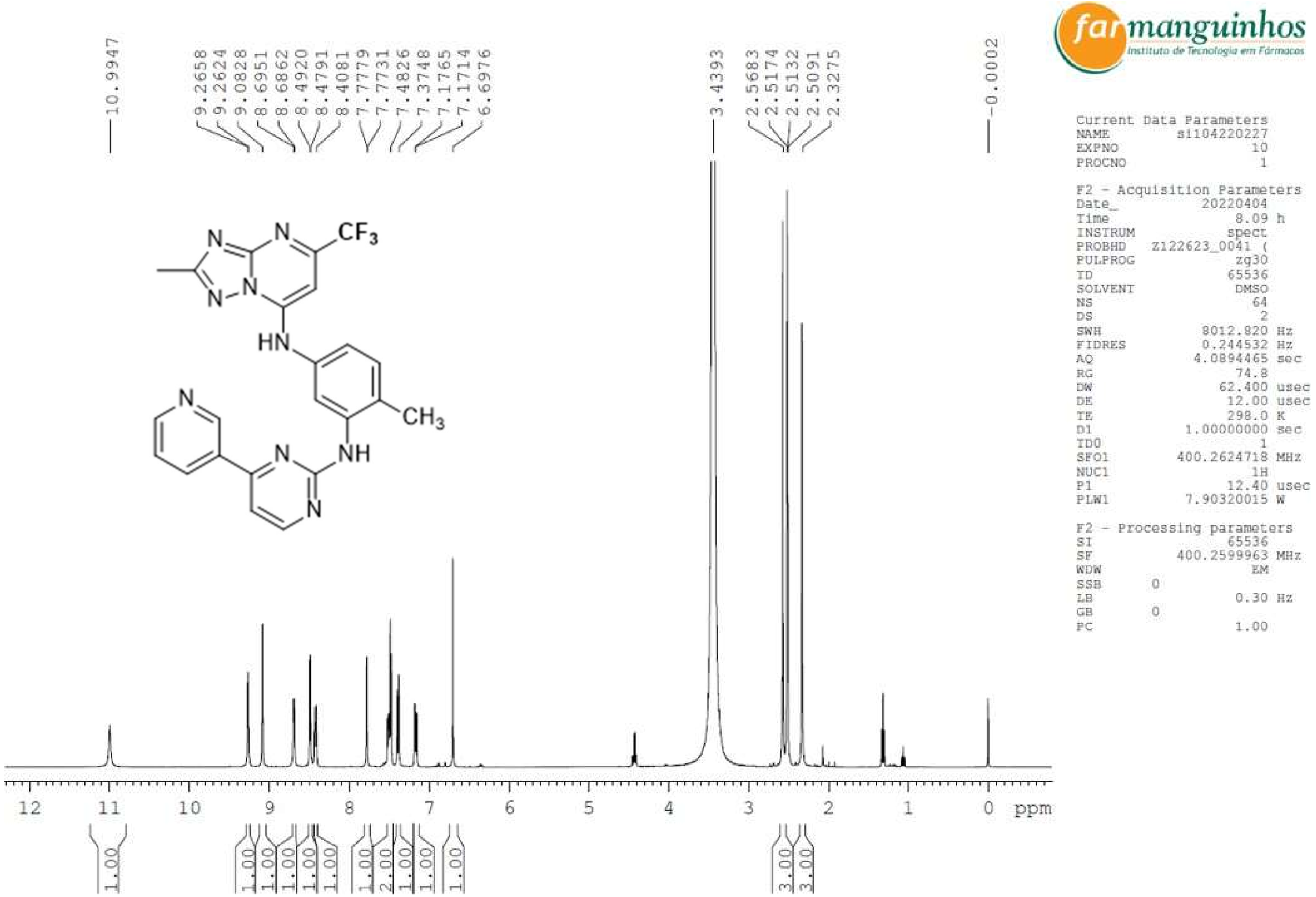

Fig-S.35: ^{1}H NMR of compound **2f**.

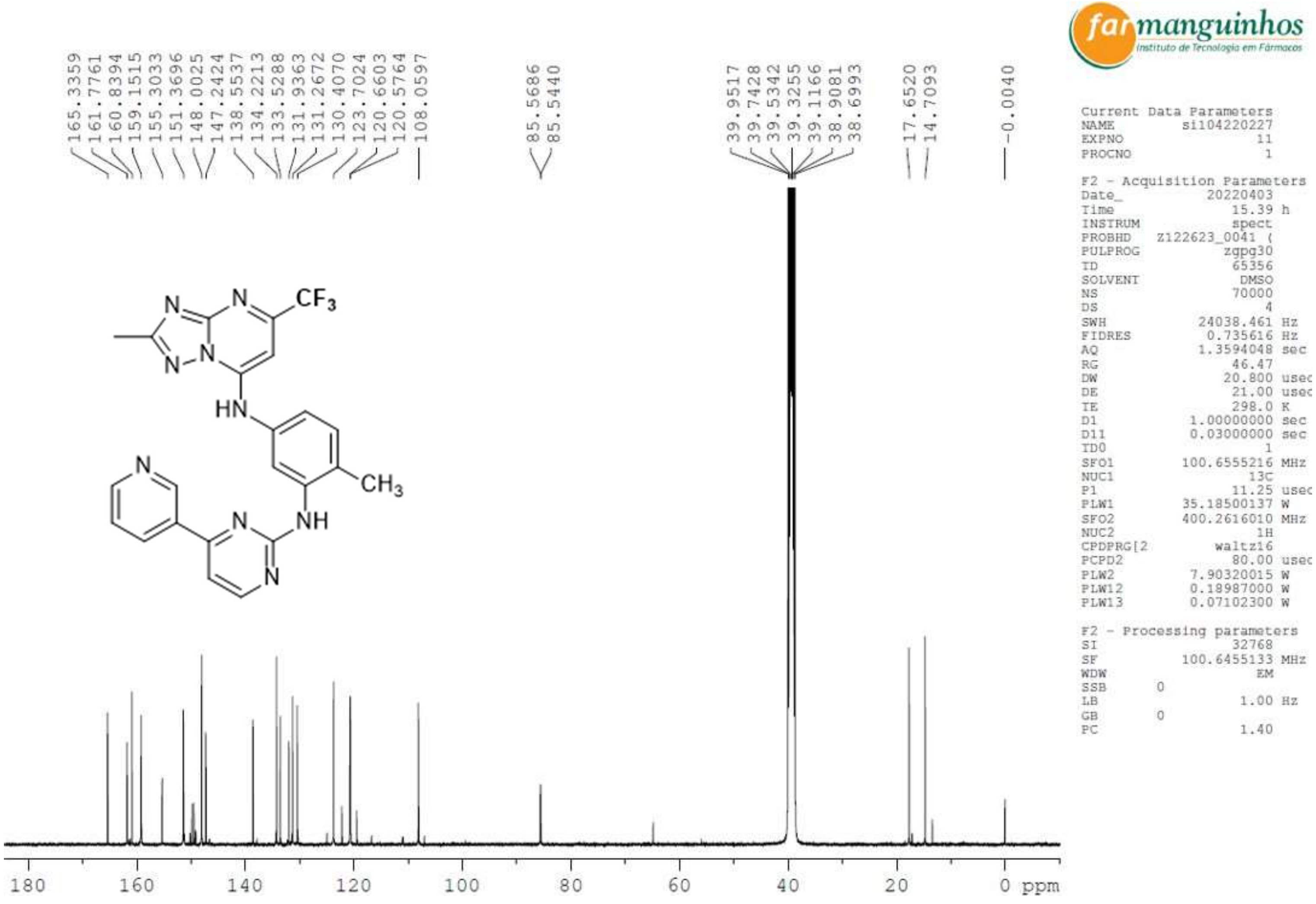

Fig-S.36: ^{13}C NMR of compound **2f**.

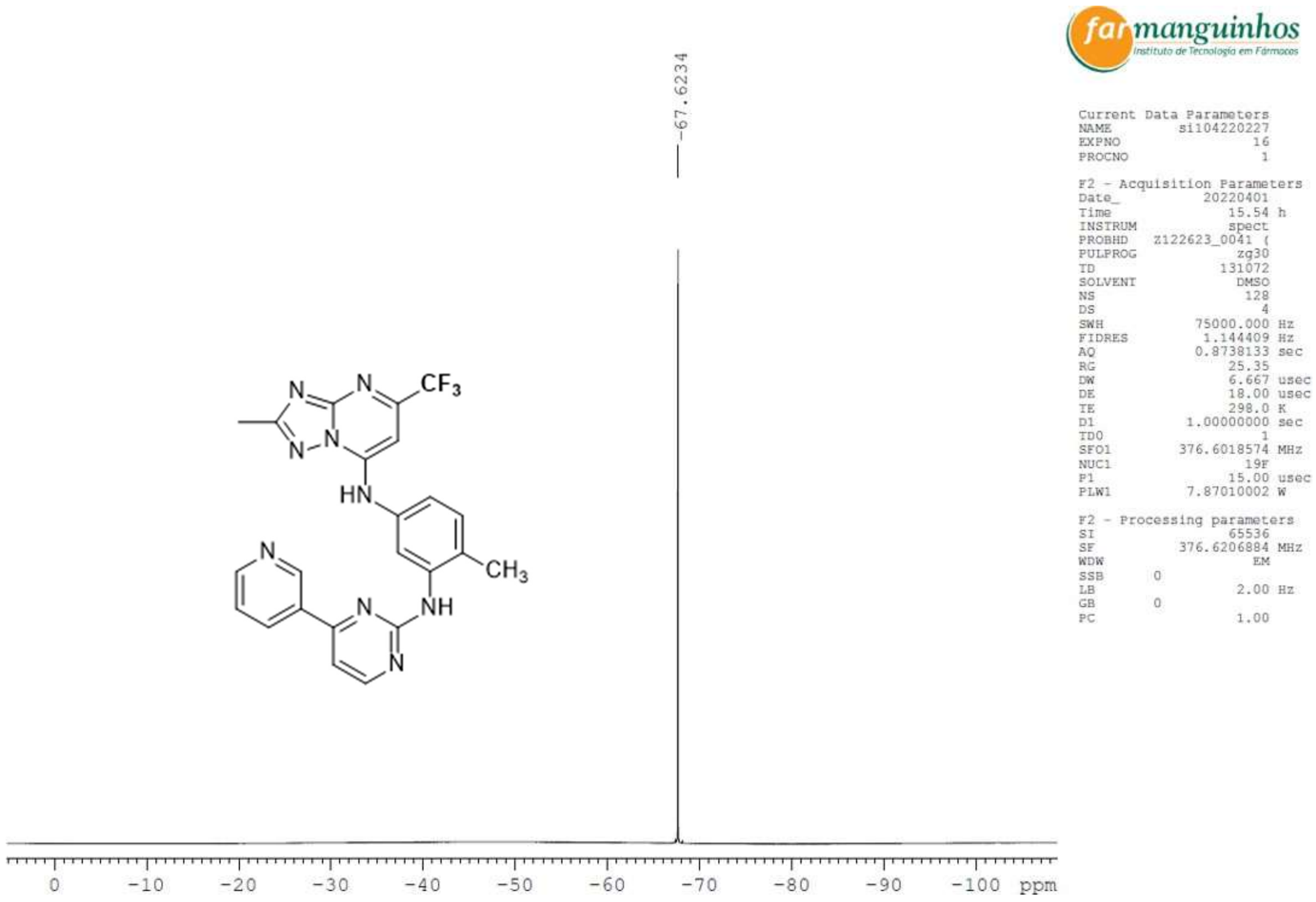

Fig-S.37: ^{19}F NMR of compound **2f**.

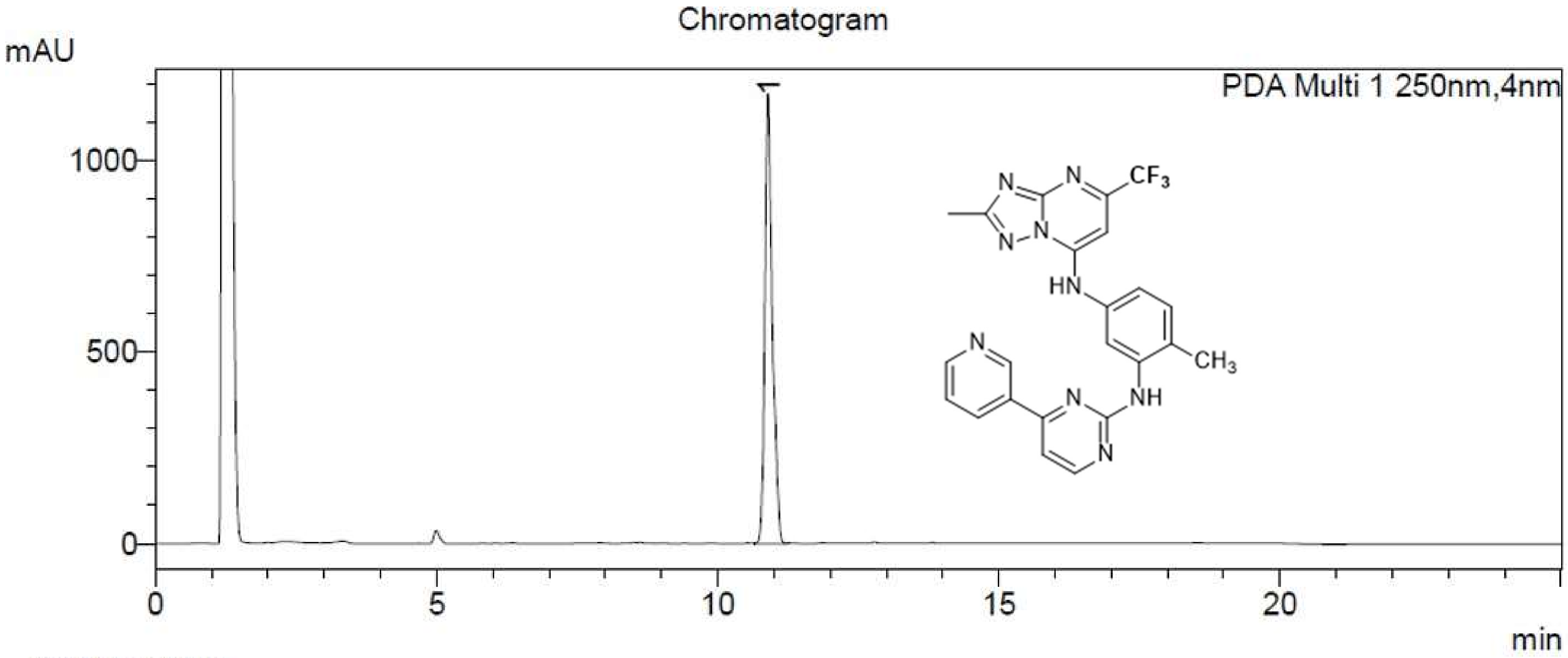

PDA Ch1 250nm

Peak#	Ret. Time	Name	Area	Area%	Theoretical Plates/meter(USP)	Tailing Factor	Resolution(USP)	Capacity Factor(k')
1	10,90		10914527	100,0	202992	1,285	--	--
Total			10914527	100,0				

Fig-S.38: HPLC-DAD of compound **2f**.
